# scIMC: a platform for benchmarking comparison and visualization analysis of scRNA-seq data imputation methods

**DOI:** 10.1093/nar/gkac317

**Published:** 2022-05-07

**Authors:** Chichi Dai, Yi Jiang, Chenglin Yin, Ran Su, Xiangxiang Zeng, Quan Zou, Kenta Nakai, Leyi Wei

**Affiliations:** College of Intelligence and Computing, Tianjin University, Tianjin, China; School of Software, Shandong University, Jinan, China; Joint SDU-NTU Centre for Artificial Intelligence Research (C-FAIR), Shandong University, Jinan, China; School of Software, Shandong University, Jinan, China; Joint SDU-NTU Centre for Artificial Intelligence Research (C-FAIR), Shandong University, Jinan, China; College of Intelligence and Computing, Tianjin University, Tianjin, China; College of Computer Science and Electronic Engineering, Hunan University, Changsha, China; Institute of Fundamental and Frontier Sciences, University of Electronic Science and Technology of China, China; Human Genome Center, Institute of Medical Science, University of Tokyo, Tokyo, Japan; School of Software, Shandong University, Jinan, China; Joint SDU-NTU Centre for Artificial Intelligence Research (C-FAIR), Shandong University, Jinan, China

## Abstract

With the advent of single-cell RNA sequencing (scRNA-seq), one major challenging is the so-called ‘dropout’ events that distort gene expression and remarkably influence downstream analysis in single-cell transcriptome. To address this issue, much effort has been done and several scRNA-seq imputation methods were developed with two categories: model-based and deep learning-based. However, comprehensively and systematically comparing existing methods are still lacking. In this work, we use six simulated and two real scRNA-seq datasets to comprehensively evaluate and compare a total of 12 available imputation methods from the following four aspects: (i) gene expression recovering, (ii) cell clustering, (iii) gene differential expression, and (iv) cellular trajectory reconstruction. We demonstrate that deep learning-based approaches generally exhibit better overall performance than model-based approaches under major benchmarking comparison, indicating the power of deep learning for imputation. Importantly, we built ***scIMC*** (**s**ingle-**c**ell **I**mputation **M**ethods **C**omparison platform), the first online platform that integrates all available state-of-the-art imputation methods for benchmarking comparison and visualization analysis, which is expected to be a convenient and useful tool for researchers of interest. It is now freely accessible via https://server.wei-group.net/scIMC/.

## INTRODUCTION

It is estimated that there is a total of 4 × 10^13^ cells in human body, which exhibit different forms and functions (1). The analysis of cell transcriptome plays an important role in characterizing the state of cell biology, which represents the complete cellular RNA transcriptome and its specificity to specific physiological conditions or specific developmental stages (2). Traditional bulk RNA sequencing (bulk RNA-seq) technique can detect the average gene expression level of a cell population. However, genes show differential expression levels in different cells. As a result, this technique is unable to quantify cell-to-cell heterogeneity. The recent development of scRNA-seq (single-cell RNA sequencing) technology enables researchers to study cell-to-cell heterogeneity of gene expression between different cells, discover novel cell types, and further improve understanding of human diseases at single-cell resolution level (3–8).

More insights into cell heterogeneity and transcriptional stochasticity can now be obtained. However, it also brings new computational challenges. The major challenge is that current technical defects, such as the low RNA capturing and sequencing efficiency, lead to failure of detection of an expressed gene, resulting in a large proportion of expressed genes with zeros or low read counts. The observed zero values do not reflect real gene expression, which is defined as ‘dropout’ events (9–11). The ‘dropout’ events introduce technical variability and high noise, making it difficult to analyze scRNA-seq data (3,12). However, not all zeros in scRNA-seq data can be considered as ‘dropout’ events. There exist true zero events, representing low-level gene expression in a specific cell type. Accordingly, it is quite challenging to distinguish ‘false’ (dropout) from ‘true’ (biological gene silencing) zero counts in scRNA-seq data. Therefore, it is an urgent need to handle the ‘dropout’ events, since it severely influences the downstream analysis, particularly with the increasing amount of scRNA-seq data.

Imputation is a common approach to recover ‘dropout’ events. Several scRNA-seq data imputation methods have been proposed in recent studies. In this study, we roughly divide existing imputation approaches into two categories: (i) model-based approaches and (ii) deep learning-based approaches. Model-based imputation methods that borrow prior knowledge and information across cells to predict the missing values, including cell-cell interaction network, gene-gene interaction network, and the integration of both of them for imputation. These methods restore the expression of the target gene in terms of similar cell information, similar gene information, and mathematical computation using prior knowledge. For example, SAVER (Single-cell Analysis Via Expression Recovery) is a method that optimizes the whole gene expression counts, using information across genes and cells to impute zeros as well as to improve the expression values for all genes (13). Similarly, MAGIC (Markov Affinity-based Graph Imputation of Cells) applies data diffusion to share information between similar cells to optimize the gene expression matrix as well as impute the missing values (14). Both MAGIC and SAVER optimize all gene expressions including those unaffected by ‘dropout’ events, meanwhile increasing the probability of introducing new noise into the rest data. To address this problem, Li *et al.* proposed a novel method named scImpute which automatically identifies possible ‘dropout’ events first, and then perform imputation to avoid introducing new noise to the rest data (15). Gong *et al.* (16) proposed DrImpute, a method using clustering results to identify multiple groups of similar cells, and perform imputation by averaging the expression values of similar cells. In addition, VIPER (Variability-Preserving Imputation for Expression Recovery) uses a sparse non-generative regression model to select a subset of the neighborhood that is most effective in predicting missing values, and then borrows information from cells in this subset that have similar expression patterns to the target cell for imputation (17). Different from the above methods using the information from scRNA-seq data only, SCRABBLE leverages bulk data as a constraint together with scRNA-seq data to impute the ‘dropout’ events (18). Combining the advantages of the popular methods (SAVER, scImpute, and MAGIC), scRecover estimates the dropout probability of each gene in each cell, and the number of expressed genes in each cell (Miao, Z., *et al.*, BioRxiv, 2019, https://doi.org/10.1101/665323). With the growing complexity of interaction network, using a priori network appropriately has become a key element of the imputation methods. scNPF shares information between similar cells, and uses the prior knowledge of the interaction network to determine gene expressions for a given cell (19). The advantage of scNPF is that it cannot only use the rich structure stored in the biological network, but also capture context-specific information to enhance the relationship between genes (19). In addition to scNPF, netNMF-sc decomposes the count matrix into two low-dimensional matrices: gene matrix and cell matrix, using network regularized non-negative matrix factorization (NMF) (20). Network regularization makes the two genes connected in the network have similar representations in the low-dimensional gene matrix, thereby restoring the data structure. Another recent imputation method, namely scTSSR (scRNA-seq using a Two-side Sparse Self-Representation), imputes the missing values using a two-side sparse self-representation model to capture the cell-to-cell and gene-to-gene similarities (21). The major difference between scTSSR and other model-based methods is that it employs both information from similar cells and similar genes for imputation (21). A recent statistical method, SDImpute (ScRNA-seq Dropout Imputation), proposed by Qi et al., uses existing gene expression data not affected by ‘dropout’ events to impute the missing values, and achieves good performance (22).

Recently, deep learning has been widely used in RNA-seq field, such as Cox-nnet (23) for prognosis prediction, and DeepMAPS (Ma, A., *et al.*, BioRxiv, 2021, https://doi.org/10.1101/2021.10.31.466658) for biological network inference, etc (24–27). Deep learning-based methods are designed to capture the hidden distribution of gene expression and learn the parameters of gene expression distribution model to impute the missing values. For example, AutoImpute is an imputation method based on autoencoder and sparse gene expression matrix. It can learn the inherent distribution of input data, and estimate the missing values with the minimal impact on biologically low-expressed genes (28). Lopea *et al.* developed single-cell Variational Inference (scVI), a method that utilizes stochastic optimization and deep neural networks to aggregate the information between similar cells and genes, and estimates the basic distribution of the count matrix (29). However, the methods mentioned above cannot intentionally preserve biological zeros and be scale to large datasets where thousands of cells are analyzed. To address this problem, an approach namely Adaptively-thresholder Low-rank Approximation (ALRA) was proposed. It is capable of selectively imputing the missing values through non-negativity and correlation structure, and effectively maintaining biological zeroes while imputing the ‘dropout’ events (Linderman, G.C., *et al.*, BioRxiv, 2018, https://doi.org/10.1101/397588). The Deep Count Autoencoder network (DCA) captures the non-linear gene-gene correlation by introducing the negative binomial noise model with zero-inflation, while considering the count distribution, over-dispersion and sparsity of the data to impute the missing values (30). Due to the low quality of scRNA-seq data and the increasing number of the measurable cell counts, more scalable imputation methods are developed (31,32). DeepImpute (Deep neural network Imputation) divides genes into target genes (genes to be imputed) and training genes (highly related to target genes, used to train neural networks to determine data distribution) for model training (33). Zhou et al. utilized transfer learning to impute the missing expression values from DNA methylation data, and developed a method, so-called TDimpute (34). Another method called DISC (Deep learning Imputation model with semi-supervised learning (SSL) for Single Cell transcriptomes) integrates an autoencoder and a recurrent neural network (RNN), and trains a semi-supervised learning model to learn the structure of genes and cells from a sparse matrix (35). Moreover, Xu *et al.* recently developed an algorithm, namely scIGANs (generative adversarial networks (GANs) for scRNA-seq Imputation), based on generative adversarial networks (36). In scIGANs, the gene expression matrix is divided into small images, and the imputation process is regarded as the process of image restoration. To utilize the similarity information between cell-to-cell as well as gene-to-gene relationships, Rao *et al.* (37) proposed a graph convolution network called GraphSCI that uses the relationship information between genes to construct a graph neural network, and learns the data distribution for imputation. Most recently, Wang *et al.* developed scGNN (single-cell Graph Neural Network), also using graph neural network to learn cell-cell relationships and combine three autoencoders to impute ‘dropout’ events and cell clustering (38).

Although a large number of imputation approaches have been proposed and most of them achieved good performance in different scenarios, comprehensively comparing the performance of state-of-the-art imputation methods are still lacking (21,34–37). In addition, systematically comparison needs to be improved with more comprehensive experiments. In this study, we evaluate and compare a total of 12 available imputation methods on six simulated datasets and two real scRNA-seq dataset in the following aspects. First, we investigated the ability of existing methods to recover true gene expression distribution. Second, we assessed the performance of cell clustering in terms of evaluating the performance of distinguishing different cell types. Third, we tested the ability of existing methods in detecting the differential expression genes by the overlap of the differential expression genes predicted by bulk RNA-seq data and scRNA-seq data, respectively. Finally, we evaluated the capability of these methods in reconstructing cellular trajectories by constructing a dynamic process. Most importantly, we established ***scIMC*** (**s**ingle-**c**ell **I**mputation **M**ethods **C**omparison platform), the first computational platform that allows researchers of interest to do data imputation and downstream comparative analysis of the state-of-the-art imputation methods on their customized datasets, and provides visualization result analysis to find out which method is most appropriate for their datasets in specific downstream tasks. We expect this platform can be convenient and useful especially for the researchers without any computer science or programing skill background in this field.

## MATERIALS AND METHODS

### Benchmarking workflow

**Figure 1.**
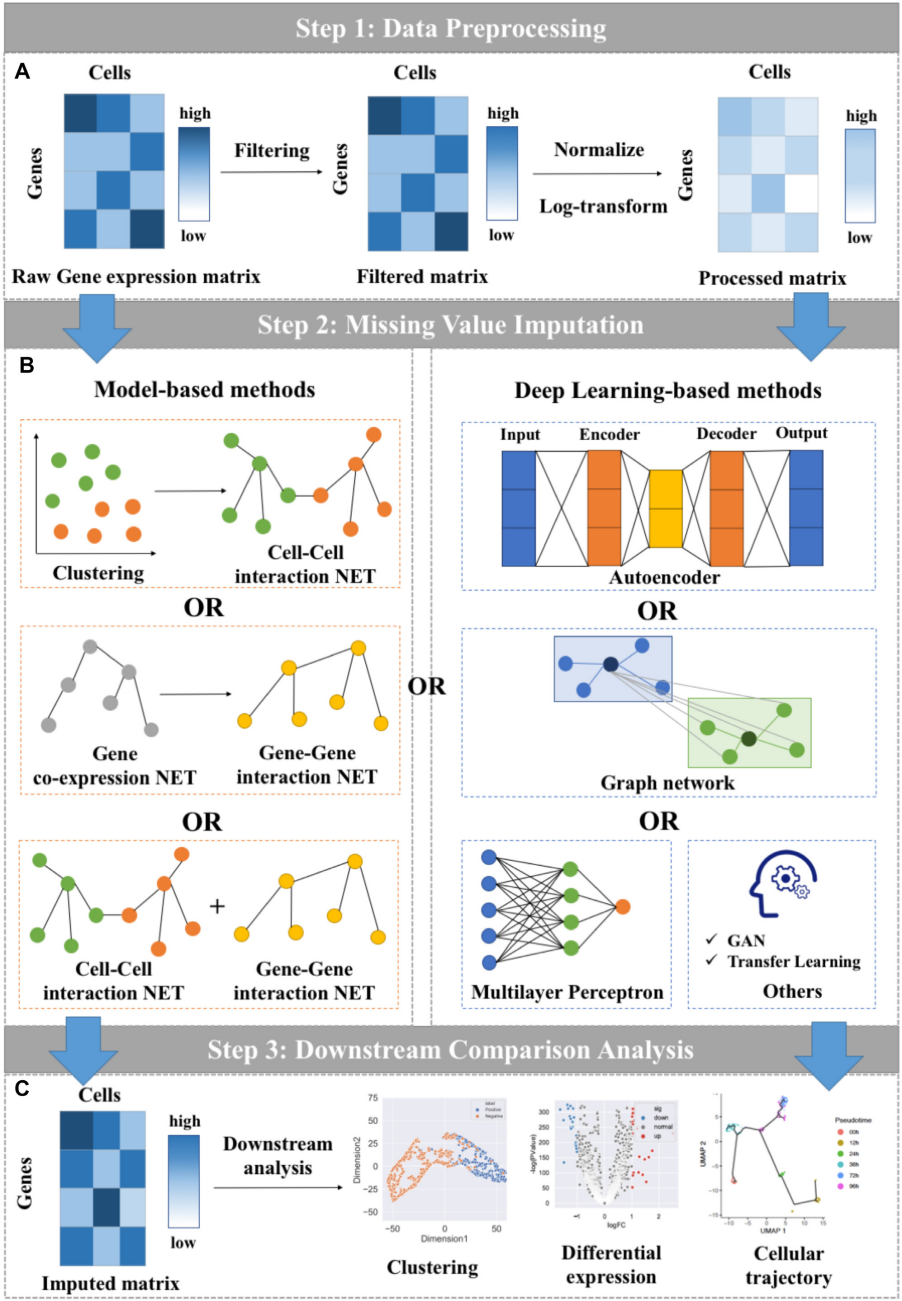
The benchmarking workflow of imputation methods. (**A**) Data Preprocessing. All datasets are filtered out by removing genes that are expressed in less than two cells, which are called low-expressed genes. We normalize the dataset by a normalization method ‘scanpy. pp. normalize_total’ from Scanpy (1.4.4) with all parameters are default. Next the normalized matrix is log-transformed. (**B**) Missing value Imputation. The methods for imputation are mainly divided into two categories: (i) model-based methods; (ii) deep learning-based methods. (**C**) Downstream Comparison Analysis. The imputed matrix is used for downstream analysis, such as clustering, differential expression analysis, etc.

In this work, we constructed an unbiased framework to quantitatively evaluate and compare the ability of available state-of-the-art imputation methods for scRNA-seq data. Based on this framework, we surveyed the performance of the imputation methods in terms of multiple widely used metrics on six simulated and two real datasets. The general overview of our benchmarking framework is illustrated in Figure 1. It can be seen that our framework is generally involved with three main steps: (A) Data Preprocessing, (B) Missing Value Imputation and (C) Downstream Comparison Analysis, which are described in details below.

**(1)** ***1st step—Data Preprocessing***. We performed the benchmarking on six simulated and two real datasets (refer to ‘Benchmark datasets for details’). For each dataset, a raw gene expression matrix (before imputation) is generally preprocessed in two sub-steps (see Figure 1A). First of all, we normalized the matrix in order to limit the data that needs to be processed (through a certain algorithm) to a certain range. Normalization is for the convenience of subsequent data processing, as well as to ensure faster convergence during program operation. Accordingly, the normalized matrix was log-transformed. The log-transformation aims to find the relationship between the data more conveniently (it can be understood as better data visualization), so that the presentation of the data is close to the assumptions we want, so as to better perform the statistical inference.
**(2)** ***2nd step—Missing Value Imputation***. Table 1 summarizes a total of 21 state-of-the-art imputation methods designed specifically for scRNA-seq data imputation, out of which there are 11 model-based approaches and 10 deep learning-based approaches. As can be seen in Table 1, the model-based approaches can be further classified into three sub-categories with regard to the information they use, such as information across cells, information across genes, and information across cells and genes. The deep learning-based approaches can be further divided based on their deep network types, including Autoencoder, MLP, Graph neural network, and Other networks. We attempted to implement all the methods, but only 12 out of them were performed successfully, which are respectively, SAVER, scTSSR, MAGIC, scImpute, DrImpute, scNPF, AutoImpute, ALRA, DCA, DeepImpute, scGNN and scIGANs. They were chosen for our comparative analysis. The algorithmic details of the 12 compared imputation methods are briefly introduced in Supplemental Materials.
**(3)** ***3rd step—Downstream Comparison Analysis***. To quantitively compare how good the imputed matrixes generated by the compared methods are, we evaluated the performance in recovering actual gene expression. As seen in Figure 1C, downstream comparative analysis is to measure their performance in real application scenarios. We further compared the methods in the following three downstream analysis tasks, including clustering analysis, differential expression analysis, and cellular trajectory analysis, etc.

**Table 1.** Brief description of state-of-the-art imputation methods

No.	Category		Methods	Language	Input	Output	Year	Reference
**1**	**Model-based approaches**	Cell and Gene-based	**SAVER**	R	G * C	G * C	2018	(13)
**2**			**SCRABBLE**	R	C * G	C * G	2019	(18)
**3**			**scRecover**	R	G * C	G * C	2019	(Miao, Z., *et al.*, BioRxiv, 2019, https://doi.org/10.1101/665323)
**4**			**netNMF-sc**	Python	G * C and G - G	G * C	2019	(20)
**5**			**scTSSR**	R	G * C	G * C	2020	(21)
**6**			**SDImpute**	R	G * C	G * C	2021	(22)
**7**		Cell-based	**MAGIC**	Python	C * G	C * G	2018	(14)
**8**			**scImpute**	R	G * C	G * C	2018	(15)
**9**			**DrImpute**	R	G * C	G * C	2018	(16)
**10**			**VIPER**	R	G * C / C * G	G * C / C * G	2018	(17)
**11**		Gene-based	**scNPF**	R	C * G	C * G	2019	(19)
**12**	**Deep learning-based approaches**	Auto-based	**AutoImpute**	Python	G * C	G * C	2018	(28)
**13**			**ALRA**	R	C * G	C * G	2018	(Linderman, G.C., *et al.*, BioRxiv, 2018, https://doi.org/10.1101/397588)
**14**			**DCA**	Python	C * G	C * G	2019	(30)
**15**			**DISC**	Python	G * C	G * C	2020	(35)
**16**		MLP-based	**scVI**	Python	C * G	C * G	2018	(29)
**17**			**DeepImpute**	Python	C * G	C * G	2019	(33)
**18**		Graph-based	**GraphSCI**	Python	G * C and G - G	G * C	2021	(37)
**19**			**scGNN**	R	G * C	G * C	2021	(38)
**20**		Other-based	**TDimpute**	Python	G * C	G * C	2020	(34)
**21**			**scIGANs**	Python	G * C	G * C	2020	(36)

* In this table, Cell and Gene-based means a method using information across cells and genes, Cell-based is a method using information across cells, and Gene-based is a method using information across genes. Moreover, Auto-based is a method based on autoencoder, MLP-based represents a method based on Multilayer Perceptron (MLP), Graph-based means a method based on graph network, and Other-based shows a method based on other networks. Besides, G * C means a gene*cell gene expression matrix, C * G means a cell*gene expression matrix, G–G means a gene–gene interaction network. The first 11 methods in this table are model-based approaches, and the other methods are deep learning-based approaches.

### Benchmark datasets

In this study, we used six simulated datasets and two real datasets to evaluate the performance of different imputation methods. The six simulated datasets were generated with different zero expression ratios. Two real datasets are Human Embryonic Stem Cells (ESCs) dataset (39) and Time-course scRNA-seq dataset (39). The details are described below.

#### Dataset 1: six simulated datasets

Splatter (40) is a R Bioconductor package proposed for simulating scRNA-seq data. We employed Splatter to generate a true counts matrix (matrix without dropouts) with 500 cells and 1000 genes. Afterwards, we set *group.prob = c(0.25, 0.25, 0.25, 0.25), mean.shape = 0.3, mean.rate = 0.02, de.prob = 0.5, de.facLoc = 0.5, dropout.shape = 0.5*, *dropout.type* = “*experiment*”, and *dropout.mid* parameter ranging from 1 to 6 in step of 1, in order to simulate six datasets with zero expression rate of 0.78, 0.71, 0.63, 0.55, 0.48 and 0.42, respectively. It is worth noting that scNPF failed to run on the simulated datasets, we only compared the other 11 methods.

#### Dataset 2: human embryonic stem cells (ESCs) dataset

We utilized a dataset with both bulk and scRNA-seq on human ESCs and differentiated definitive endoderm cells (DECs), including six samples of bulk RNA-seq (four for H1 ESC and two for DEC) and 350 cells of scRNA-seq (212 cells for H1 ESC and 138 cells for DEC) (39). The percentage of zero in gene expression matrix of bulk RNA-seq data and scRNA-seq data are 14.8% and 49.1%, respectively. We used this dataset to evaluate the ability of imputation methods in capturing differentially expressed genes (DEGs). If a gene with the *P*-value (*P*-value is significant index) is <0.05, we consider it to be differentially expressed, so-called DEG. We performed edgeR (41) on both bulk RNA-seq data and scRNA-seq data to detect DEGs, respectively. Considering the DEGs of bulk RNA-seq data as a golden standard, the performance of different methods in capturing DEGs is defined as the overlapping between DEGs detected by bulk RNA-seq data and those detected by scRNA-seq data (21). It is worth noting that amongst the 12 compared imputation methods, DCA failed to perform on this dataset due to its intrinsic limitation.

#### Dataset 3: time-course scRNA-seq dataset

We employed the time-course scRNA-seq data derived from the differentiation from H1 ESC to DEC (39). This dataset consists of 758 cells, including 92 cells at 0 h, 102 cells at 12 h, 66 cells at 24 h, 172 cells at 36 h, 138 cells at 72 h and 188 cells at 96 h after the differentiation from H1 ESCs to DECs. In order to evaluate the performance of imputation methods for reconstructing the trajectories, we performed existing imputation methods on this dataset, and used Monocle3 (42) to reconstruct the trajectories. Notably, amongst the compared methods, DCA and scGNN failed to perform on this dataset due to their intrinsic limitation.

### Data Preprocessing

For the scRNA-seq data imputation, the standard data preprocessing procedure contains three steps: filtering, normalization, and log-transformation. For all datasets used in this study, we firstly filtered out those genes that are expressed in less than two cells, which are called low-expressed genes. Next, we normalized the dataset by a normalization method ‘scanpy. pp. normalize_total’ from Scanpy (1.4.4) with being divided by library size and multiplied by the median of library size across cells. Finally, the normalized matrix is log-transformed. It's worth to point out that five out of the 12 compared methods, including scImpute, DrImpute, scTSSR, AutoImpute, and scGNN, have the data preprocessing module in their source codes. Besides, DCA and DeepImpute only accept the dataset without normalization and log-transformation. Therefore, we input the raw gene expression matrix to DrImpute, AutoImpute, and scGNN and did only the filtering step to the raw gene expression matrix for the four methods (scImpute, DCA, DeepImpute and scTSSR). To keep data consistence, we followed the above data preprocessing steps to process the data before feeding to the rest methods (SAVER, ALRA, MAGIC, scNPF and scIGANs) for imputation. The shape of imputed matrix from different imputation methods are shown in Table 2.

**Table 2.** Shape of imputed matrix of different imputation methods

Methods	D1	D2	D3	D4	D5	D6	D7	D8
scImpute	500*961	500*961	500*962	500*961	500*964	500*964	350*16383	758*16383
SAVER	500*961	500*961	500*962	500*961	500*964	500*964	350*16383	758*16383
MAGIC	500*961	500*961	500*962	500*961	500*964	500*964	350*16383	758*16383
DCA	500*961	500*961	500*962	500*961	500*964	500*964	–	–
ALRA	500*961	500*961	500*962	500*961	500*964	500*964	350*16383	758*16383
DrImpute	500*933	500*939	500*939	500*939	500*941	500*941	350*16604	758*16842
DeepImpute	500*961	500*961	500*962	500*961	500*964	500*964	350*16383	758*16383
scTSSR	500*888	500*903	500*907	500*906	500*909	500*911	350*13711	758*13037
scNPF	–	–	–	–	–	–	350*16383	758*16383
AutoImpute	500*883	500*891	500*893	500*898	500*899	500*900	350*1000	758*1000
scIGANs	500*961	500*961	500*962	500*961	500*964	500*964	350*16383	758*16383
scGNN	500*938	500*945	500*943	500*945	500*945	500*946	350*2000	–

* In this table, the shape is shown as gene*cell. For example, 500*961 means a matrix with 500 cells and 961 genes. – means this imputation method did not run on this dataset. **D1–6** are simulated datasets in Dataset 1, **D7** represents Dataset 2, and **D8** is corresponding to Dataset 3.

### Performance evaluation

In this section, the gene expression matrix is denoted as *X* (true gene expression matrix in RMSE and PCC), and $\hat{X}$ is the imputed matrix. To quantitatively evaluate the performance of different imputation methods in recovering gene expression, we use two metrics, root mean square error (RMSE) and Pearson correlation coefficient (PCC). For evaluation and comparison of clustering results and gene differential expression results, we use five common metrics: normalized mutual information (NMI), adjusted Rand index (ARI), silhouette coefficient (Si score), Jaccard similarity coefficient (Jaccard), and Purity. As for the comparison of cellular trajectories, we deploy the other two metrics: pseudo-temporal ordering score (POS) and Kendall's rank correlation score (KOR). The above metrics are described in details as follows.

#### Root mean square error (RMSE)

It is to measure the difference between the imputed matrix and the raw matrix, which calculates the deviation between the observed values and true values. RMSE is defined as:

(1) $$\begin{equation*}{\rm{RMSE}}\left( {X,\hat{X}} \right) = \sqrt {\frac{1}{n}\mathop \sum \limits_{i = 1}^n {{\left( {\widehat {{X_i}} - {X_i}} \right)}^2}} \end{equation*}$$

#### Pearson correlation coefficient (PCC)

It is to examine the degree of correlation between the imputed matrix and the raw matrix, which is defined as:

(2) $$\begin{equation*}{\rho _{{\rm{X}},\hat{X}}} = \frac{{E\left( {{\rm{X}}\hat{X}} \right) - E\left( {\rm{X}} \right)E\left( {\hat{X}} \right)}}{{\sqrt {E\left( {{X^2}} \right){E^2}\left( X \right)} \sqrt {E\left( {{{\hat{X}}^2}} \right){E^2}\left( {\hat{X}} \right)} }}\end{equation*}$$

where $E( X )$ represents the mean of *X*, ${E^2}( X )$ is the square of $E( X )$.

#### Normalized mutual information (NMI)

It refers to the degree of correlation between two random variables. We denote $label$ as the original cluster label set, and $\widehat {label}$ as the label set obtained by clustering. The calculation formula of NMI is as follows:

(3) $$\begin{equation*}NMI = {\rm{ }}2\frac{{I{\rm{ }}\left( {label,\widehat {label}} \right)}}{{H\left( {label} \right) + H{\rm{ }}\left( {\widehat {label}} \right)}}\end{equation*}$$

where $I\ ( {label,\widehat {label}} )$ is:

(4) $$\begin{eqnarray*} && I\left( {label,\widehat {label}} \right) \nonumber \\ && \quad = \mathop \sum \limits_{a \in label,b \in \widehat {label}} p{\rm{\ }}\left( {a,b} \right)\log \frac{{p{\rm{\ }}\left( {a,b} \right)}}{{p{\rm{\ }}\left( a \right)p{\rm{\ }}\left( b \right)}} \end{eqnarray*}$$

${\rm{H}}( {label} )$ is:

(5) $$\begin{equation*}H\left( {label} \right) = \mathop \sum \limits_{a \in label} p\left( a \right)\log p{\rm{\ }}\left( a \right)\end{equation*}$$

where $p\ ( a )$,$p\ ( b )$, and $p\ ( {a,\ b} )$ represent the probability that the sample belongs to the cluster *a*, the probability that the sample belongs to the cluster *b*, and the probability that the sample belongs to both *a* and *b*, respectively.

#### Adjusted Rand index (ARI)

It measures the degree of agreement between the two data distributions (43). We assume that there are *m* cells which are cluster into *k* clusters. $\{ {{u_i}} \}_i^m$ represents the predicted cluster label, as well as $\{ {{v_j}} \}_j^m$ denotes the true cluster label. The calculation formula of ARI is as follows:

(6) $$\begin{equation*}{\rm{ARI}} = \frac{{\mathop \sum \nolimits_{i{\rm{j}}} \left( {\begin{array}{@{}*{1}{c}@{}} {{n_{ij}}}\\ 2 \end{array}} \right) - \left[ {\mathop \sum \nolimits_i \left( {\begin{array}{@{}*{1}{c}@{}} {{a_i}}\\ 2 \end{array}} \right)\mathop \sum \nolimits_{\rm{j}} \left( {\begin{array}{@{}*{1}{c}@{}} {{b_j}}\\ 2 \end{array}} \right)} \right]\Big/\left( {\begin{array}{@{}*{1}{c}@{}} n\\ 2 \end{array}} \right)}}{{\frac{1}{2}\left[ {\mathop \sum \nolimits_i \left( {\begin{array}{@{}*{1}{c}@{}} {{a_i}}\\ 2 \end{array}} \right) + \mathop \sum \nolimits_{\rm{j}} \left( {\begin{array}{@{}*{1}{c}@{}} {{b_j}}\\ 2 \end{array}} \right)} \right] - \left[ {\mathop \sum \nolimits_i \left( {\begin{array}{@{}*{1}{c}@{}} {{a_i}}\\ 2 \end{array}} \right)\mathop \sum \nolimits_{\rm{j}} \left( {\begin{array}{@{}*{1}{c}@{}} {{b_j}}\\ 2 \end{array}} \right)} \right]\Big/\left( {\begin{array}{@{}*{1}{c}@{}} n\\ 2 \end{array}} \right)}}\end{equation*}$$

where *i* and *j* enumerate the *k* clusters, and ${n_{ij}} = \mathop \sum \limits_{k,g} I( {{u_k} = i} )I( {{v_g} = j} )$, ${a_i} = \mathop \sum \limits_k I( {{u_k} = i} )$, and ${b_j} = \mathop \sum \limits_g I( {{v_g} = j} )$. The indicator function $I( {x{\rm{ }} = {\rm{ }}y} )$ is defined as follows:

(7) $$\begin{equation*}I\left( {x = {\rm{ }}y} \right) = \left\{ \begin{array}{@{}*{1}{l}@{}} {1,\ \ x = y}\\ {0,\ \ otherwise} \end{array} \right. \end{equation*}$$

#### Silhouette coefficient (Si score) (44)

It is used to evaluate the cell clustering performance of imputation methods. It combines cohesion and separation, which can evaluate the clustering results on the same data. The closer the Si score is to 1, the more accurate the clustering is; the closer it is to –1, the worse the result is. The Si score is defined as:

(8) $$\begin{equation*}Si = \frac{{{b_i} - {a_i}}}{{max\left( {{b_i},{a_i}} \right)}}\end{equation*}$$

where ${a_i}$ represents the average distance between the *i*-th sample and all other samples in the same cluster, ${b_i}$ represents the average distance between the *i*th sample and all samples in a given cluster (clusters that does not contain the *i*th sample).

#### Jaccard similarity coefficient (Jaccard)

Moreover, we utilize Jaccard similarity coefficient (Jaccard) (45) to evaluate the gene differential expression performance of imputation methods. Jaccard is used to compare the similarities between samples. The larger the Jaccard coefficient value, the higher the similarity of samples. Jaccard is defined as:

(9) $$\begin{equation*}{\rm{J}}\left( {A,B} \right) = \frac{{\left| {A\mathop \cap \nolimits B} \right|}}{{\left| {A\mathop \cup \nolimits B} \right|}} = \frac{{\left| {A\mathop \cap \nolimits B} \right|}}{{\left| A \right| + \left| B \right| - \left| {A\mathop \cap \nolimits B} \right|}}\end{equation*}$$

where *A* and *B* are two sets. Jaccard is the ratio of the size of the intersection of *A* and *B* to the size of the union of *A* and *B*.

#### Purity

It is a commonly used evaluation metric for clustering. We assume that there are *m* samples belonging to *K* clusters, respectively. Purity is defined as:

(10) $$\begin{equation*}purity = {\rm{ }}\mathop \sum \limits_{i = 1}^K \frac{{{m_i}}}{m}{P_i}\end{equation*}$$

where ${m_i}$ represents the number of samples in cluster *i*; ${P_i} = {\rm{max}}( {{P_{ij}}} )$, where ${P_{ij}}$ is the probability that the sample in cluster *i*, but belongs to cluster *j*, which is calculated as:

(11) $$\begin{equation*}{P_{ij}} = \frac{{{m_{ij}}}}{{{m_i}}}\end{equation*}$$

where ${m_{ij}}$ is the number of samples in cluster *i*, but belongs to cluster *j*.

#### Pseudo-temporal ordering score (POS)

It can be used to evaluate cell order performance. The formula of POS is:

(12) $$\begin{equation*}POS = \mathop \sum \limits_{i = 1}^{n - 1} \mathop \sum \limits_{j >i} g\left( {i,j} \right)\end{equation*}$$

where *n* is the number of samples, $g( {i,j} )$ is a score that characterizes how well the order of the *i*th and *j*th cells in the ordered path matches their expected order based on the external information (46).

#### Kendall's rank correlation score (KOR)

It is often used to measure the degree of correspondence between two rankings. It is defined as:

(13) $$\begin{equation*}{\rm{\tau }} = \frac{{4P}}{{n\left( {n - 1} \right)}} - 1\end{equation*}$$

where *n* is the number of samples, and *P* is the sum of the number of samples ranked after the given sample by both rankings.

### Downstream analysis tools

#### Differential expression genes (DEG) analysis

ScRNA-seq data can provide insights into the randomness of gene expressions, which determines different types of cells. To perform DEG analysis, we ran edgeR (41) on scRNA-seq data, with all parameters are default. The results include *fold change* and *P-value*. *Fold change* represents the multiple of difference, and *P-value* is significant index. When the *P-value* of a gene is <0.05, we consider it to be differentially expressed.

#### Principal component analysis (PCA)

It is used to preprocess and visualize our scRNA-seq data (47). We implemented PCA with the default parameters using the sklearn package in the Python environment to preprocess and visualize the raw data and the imputed output.

#### T-distributed stochastic neighbor embedding (t-SNE)

T-SNE (48), a common dimension reduction and visualization tool, is also used to preprocess and visualize our scRNA-seq data. We implemented t-SNE with the default parameters using the sklearn package in the Python environment to preprocess and visualize the raw data and the imputed output.

#### Uniform manifold approximation and projection (UMAP)

UMAP is the latest dimension reduction algorithm, which was proposed in 2018 (49). In this paper, UMAP was implemented by the sklearn package in the Python environment with default parameters. The raw data and imputed data in this study were processed and visualized by UMAP.

#### Monocle3

It is utilized to reconstruct cellular trajectories of scRNA-seq data (42). We implemented Monocle3 with the default parameters in the R (3.6.3) environment. Notably, UMAP (49) is the default visualization method of Monocle3, and it is used to visualize the cellular trajectories of data.

#### Tools for single cell analysis (TSCAN)

It is a tool developed to reconstruct pseudo-time trajectories in scRNA-seq analysis (46). It orders cells via a cluster-based minimum spanning tree approach. In this paper, we utilized TSCAN with default parameters.

### Method implementing details

In the process of implementing the 12 imputation methods, there are four methods that set non-default parameters: (1) in MAGIC, we set the parameter “*genes = all_genes*”, which means that the result will return the whole smooth matrix; (2) in ALRA, we set “*k = choose_k*”, *k* represents the rank of low-rank approximation, and *choose_k* is the method of selecting rank *k* of low-rank approximation based on continuous singular value spacing statistics designed in advance in ALRA; (3) Set the network type to “*network = context*” in scNPF. This is because the default value read in this parameter is null, so we selected the default type in this method. (4) Set “*LTMG = TRUE*” in scGNN, which means that the Left Truncated Mixture Gaussian (LTMG) model (38) is used to model the scRNA-seq data, and establish cell maps of cell type specific regulatory signals. In addition, the number of clusters filled in all methods corresponds to the clusters number of the dataset used. The other methods are implemented for comparison using default parameters given in their source codes.

## RESULTS

### Comparison of imputation methods for recovering gene expression

**Figure 2.**
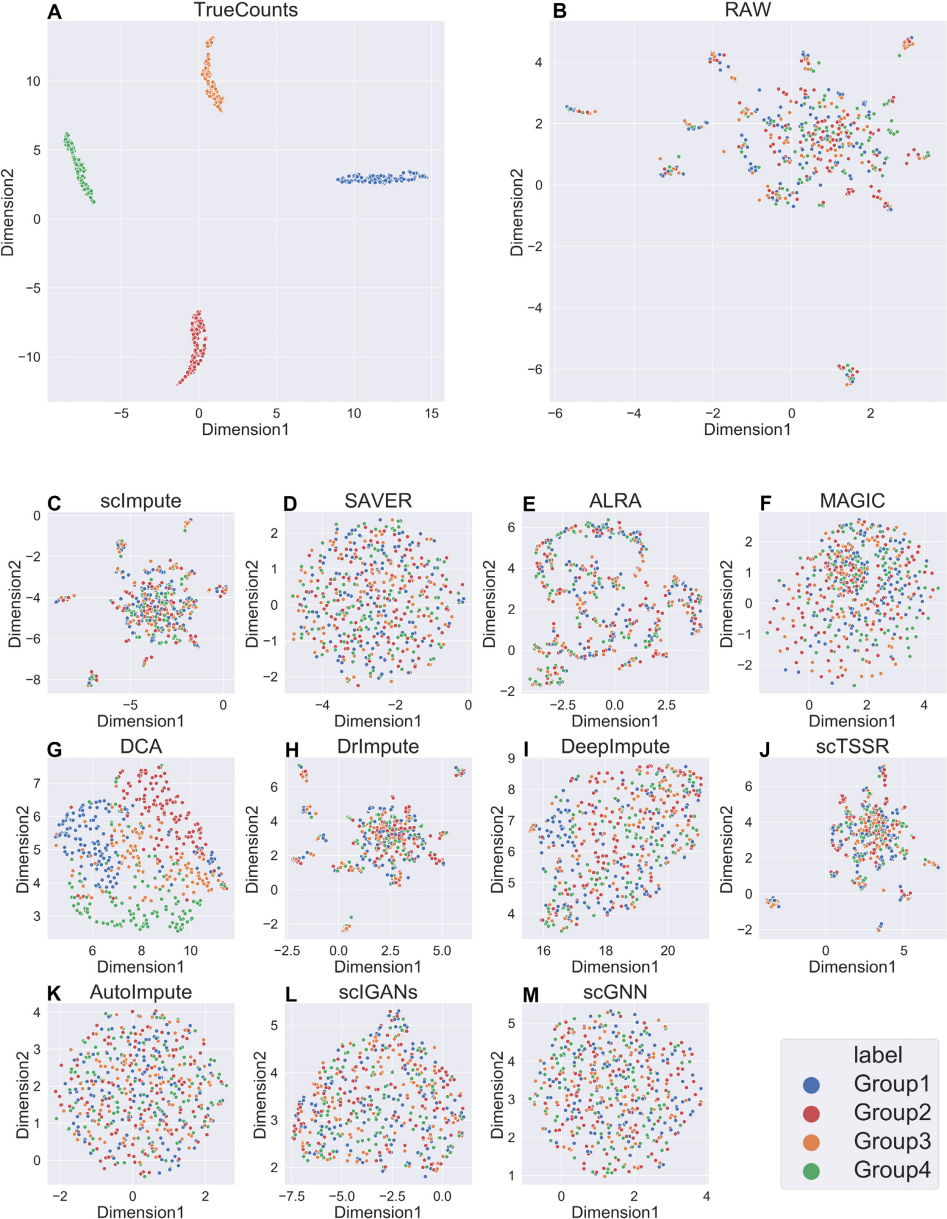
UMAP plots of gene expression distribution of 11 methods on simulated dataset with zero expression rate of 0.78. (**A–M**) UMAP plots on true counts matrix (without dropouts), raw gene expression matrix (with dropouts), and imputed matrices by scImpute, SAVER, ALRA, MAGIC, DCA, DrImpute, DeepImpute, scTSSR, AutoImpute, scIGANs and scGNN, respectively.

**Figure 3.**
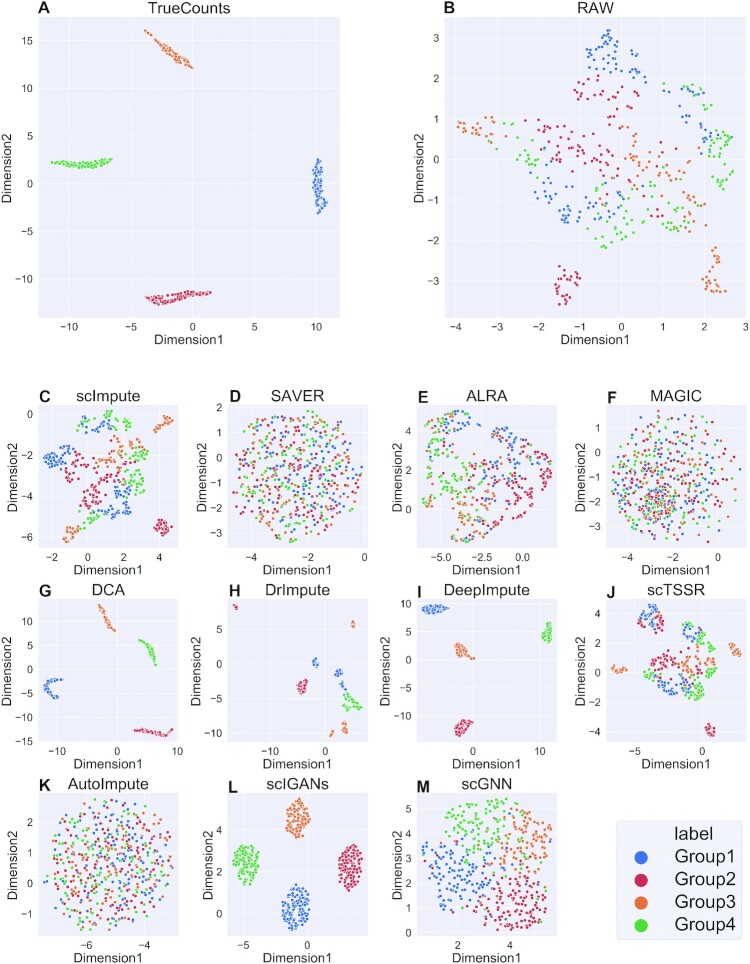
UMAP plots of gene expression distribution of 11 methods on simulated dataset with zero expression rate of 0.42. (**A–M**) UMAP plots on true counts matrix (without dropouts), raw gene expression matrix (with dropouts), and imputed matrices by scImpute, SAVER, ALRA, MAGIC, DCA, DrImpute, DeepImpute, scTSSR, AutoImpute, scIGANs and scGNN, respectively.

**Figure 4.**
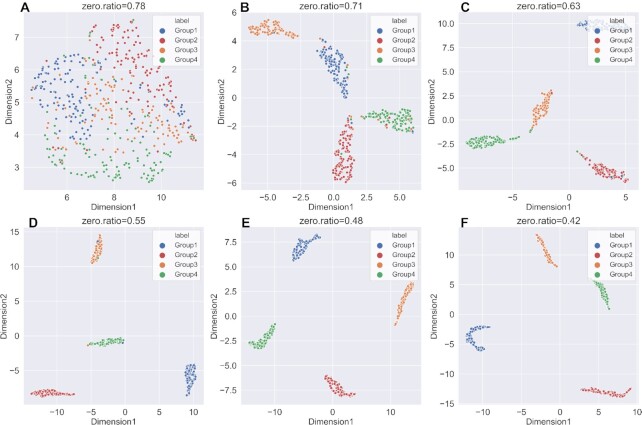
UMAP plots of imputed data for DCA in six simulated datasets with different zero expression rates. (**A–F**) UMAP plots on simulated dataset with zero expression rate of 0.78, 0.71, 0.63, 0.55, 0.48 and 0.42.

**Figure 5.**
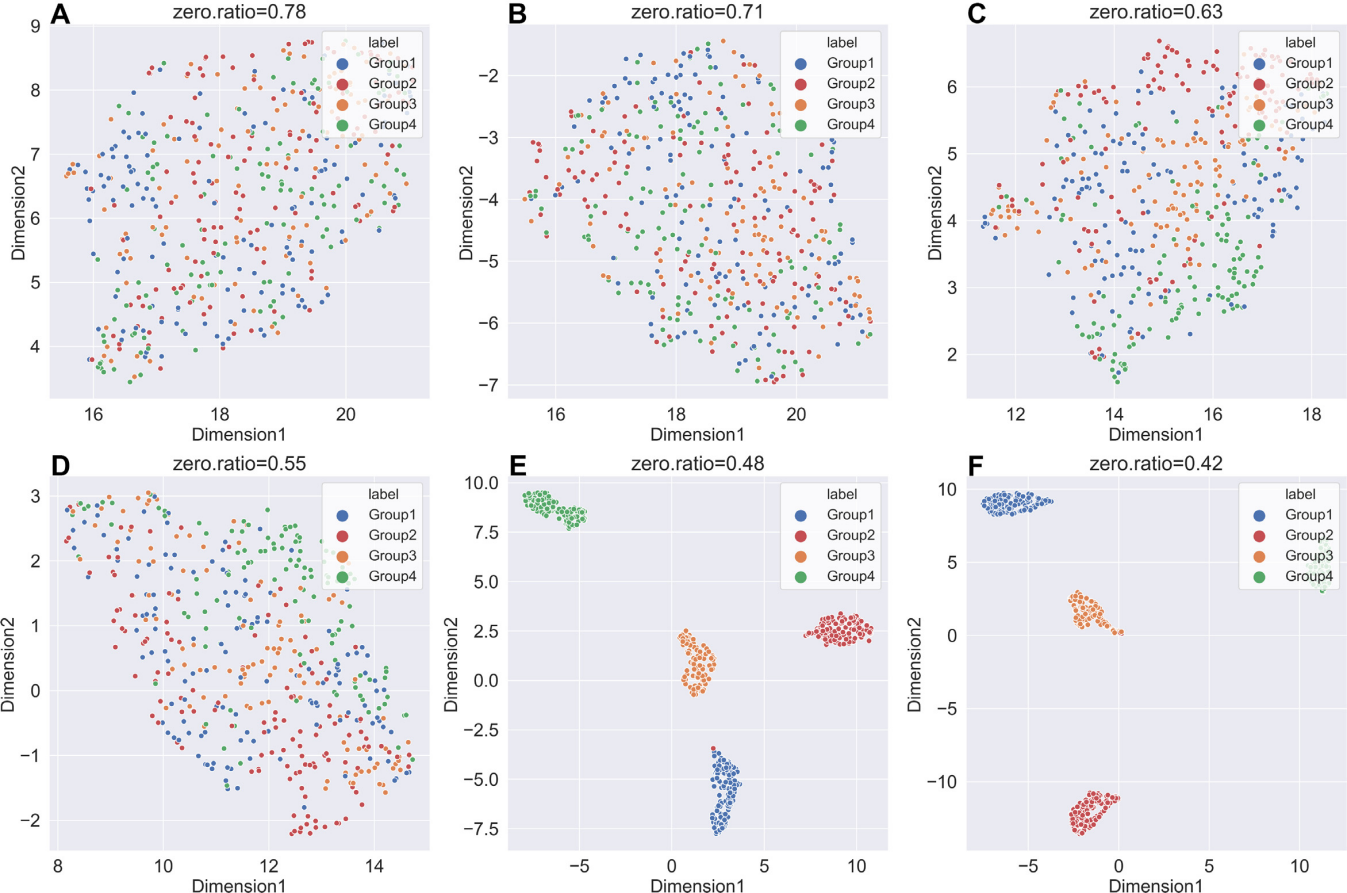
UMAP plots of imputed data for DeepImpute in six simulated datasets with different zero expression rates. (**A–F**) UMAP plots on simulated dataset with zero expression rate of 0.78, 0.71, 0.63, 0.55, 0.48 and 0.42.

**Figure 6.**
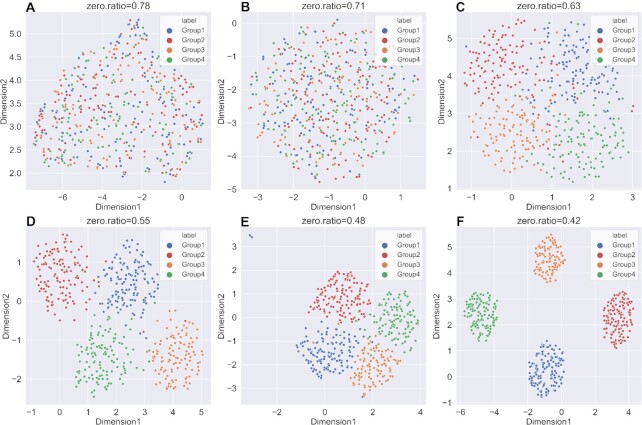
UMAP plots of imputed data for scIGANs in six simulated datasets with different zero expression rates. (**A–F**) UMAP plots on simulated dataset with zero expression rate of 0.78, 0.71, 0.63, 0.55, 0.48 and 0.42.

A good imputation method should recover the true gene expression of scRNA-seq data. Due to the lack of ground truth of expression values in the real datasets, we generated six simulated datasets with different parameters using Splatter. It was worth noting that scNPF failed to run on the simulated datasets, we only compared the other 11 methods. We firstly visualized true counts data (data without dropouts), raw data (data with dropouts but not imputed) and imputed data from 11 imputation methods, using UMAP (49), a commonly used dimension reduction and visualization tool (Figures 2 and 3 and Supplemental Materials Figures S1–S4). The results of dataset with zero expression rate of 0.78 and 0.42 were illustrated in Figures 2 and 3, respectively. We can observe from Figures 2 and 3 that the result of true counts matrix (matrix without dropouts) had four cell subpopulations with clear border, and other results were affected by dropout noise. No matter how high zero expression rate was, DCA outperformed among other methods, distinguishing four different clusters. Besides, it can be seen from Figure 3 that DeepImpute and scIGANs recovered the gene expression accurately when the zero expression rate decreased. To investigate if different zero expression rates can affect the comparison results, we instead used UMAP (49) to visualize the results of DCA, DeepImpute and scIGANs on six simulated datasets (the results of other methods are shown in Supplemental Materials, Figures S5–S12). In Figures 4–6, we can observe the following results: (i) When the zero expression rate of the dataset was 0.78, data were actually distributed in four clusters without clear boundaries; (ii) in dataset with zero expression rate of 0.42, the margins between different clusters were more clearly separated; (iii) we can see four distinct clusters clearly in dataset with lowest zero expression rate, which accounted for the best performance of DCA, DeepImpute and scIGANs. We also visualized the above results by PCA and t-SNE, which can be found in Supplemental Materials (Supplementary Figures S13–S35). We found that the results by PCA and t-SNE were similar to those by UMAP. However, the visualization of PCA were obviously worse than those of UMAP and t-SNE, which cannot show clear boundaries between four different cell clusters.

**Figure 7.**
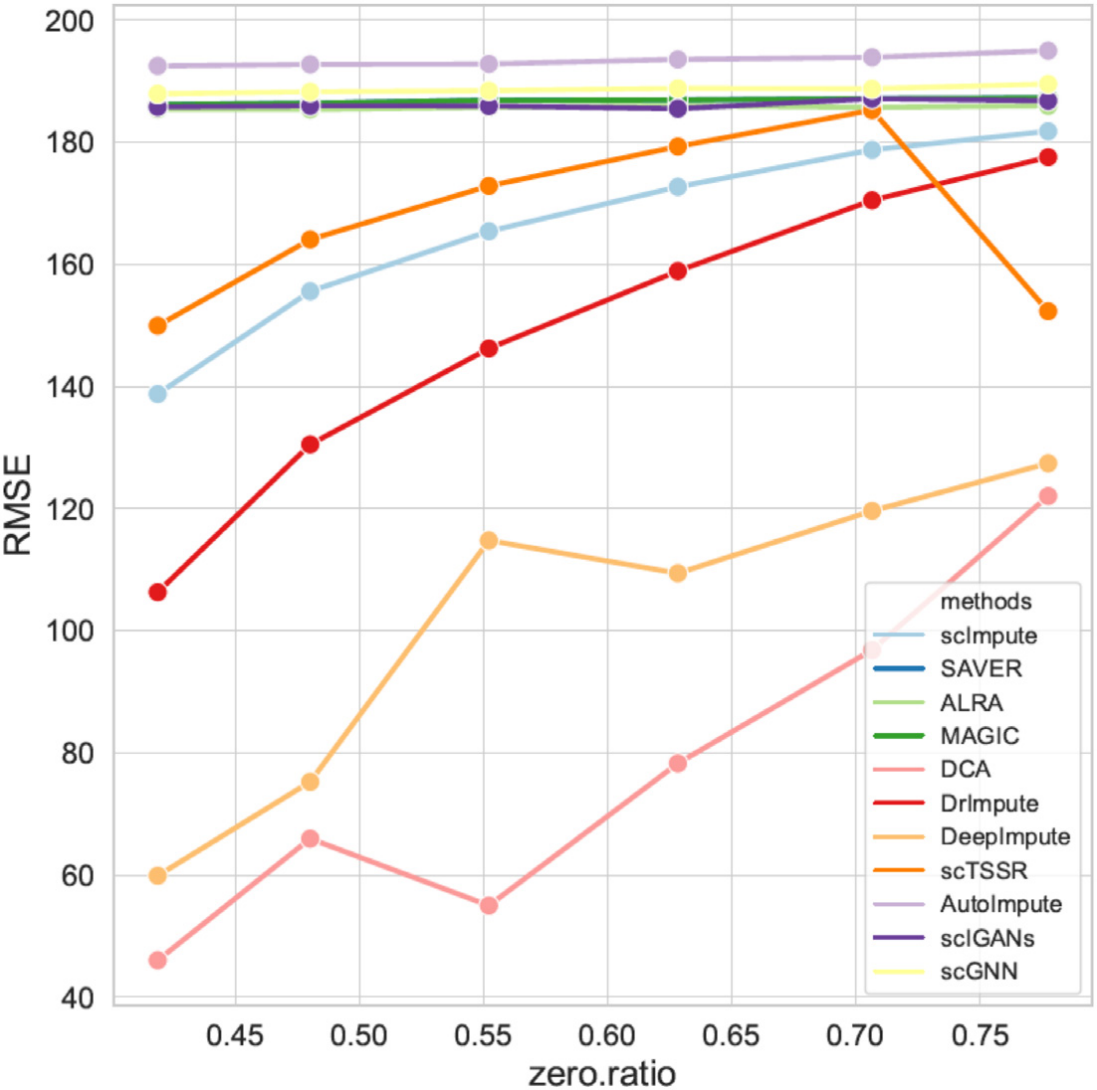
RMSEs of imputed data for recovering gene expression with different zero expression rates. RMSE between true counts data and imputed data on six simulated datasets.

**Figure 8.**
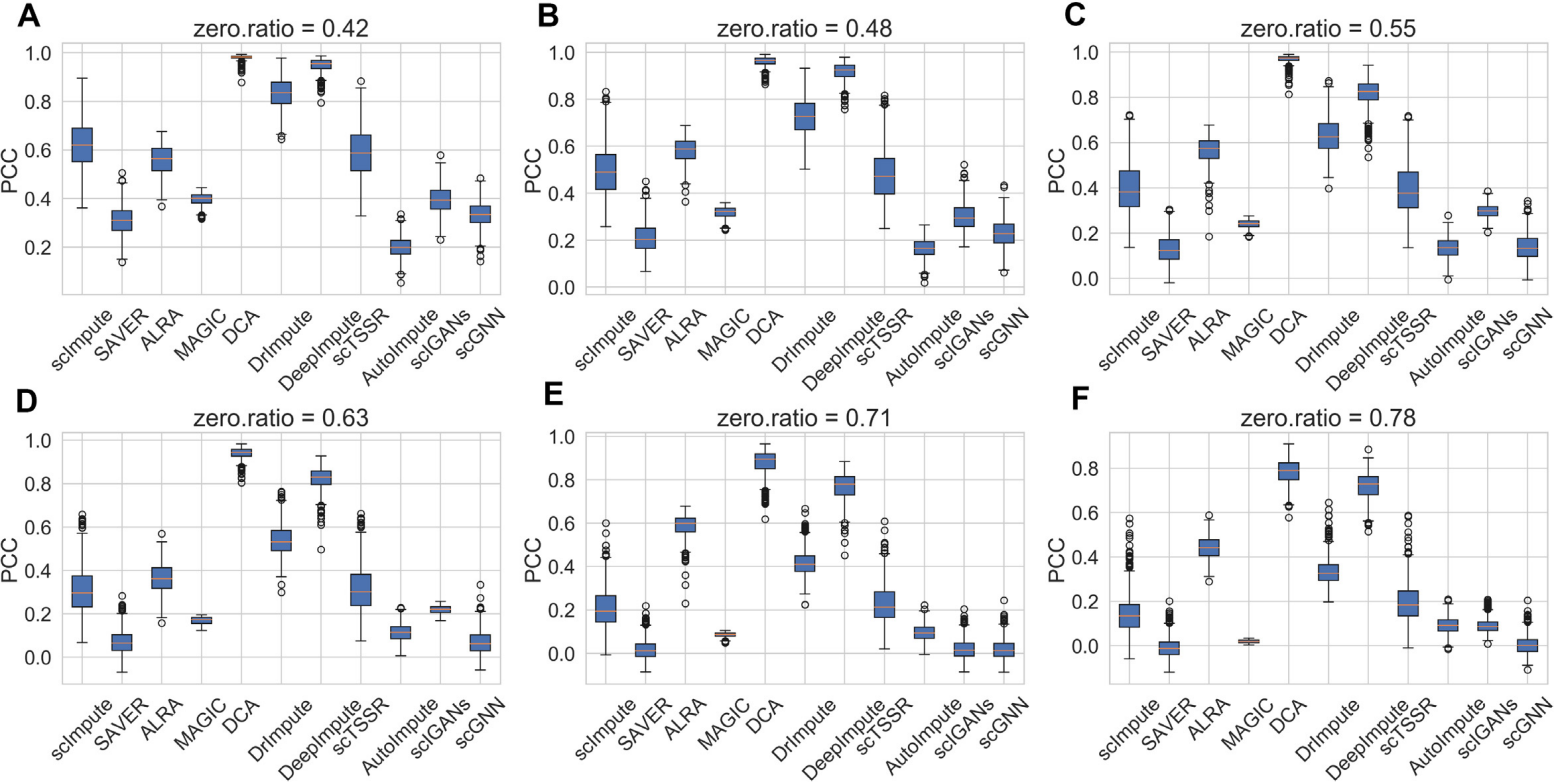
PCC of imputed data for recovering gene expression on six simulated datasets with different zero expression rates. (**A–F**) PCC on simulated dataset with zero expression rate of 0.42, 0.48, 0.55, 0.63, 0.71 and 0.78.

To quantitively compare the ability of different imputation methods for recovering true gene expression, we further used two metrics, RMSE and PCC, to evaluate the performance of the methods. In order to explore the performance of different methods on datasets with different zero expression rates, we simulated six datasets with different zero expression rates. We performed the imputation methods on the six simulated datasets and calculated the RMSEs and PCCs between true count matrix (matrix without dropouts) and the imputed matrix, respectively. The results are illustrated in Figures 7 and 8, where we can observe that as zero expression rate increased, the RMSEs of all imputation methods increased, while their PCCs decreased. DCA and DeepImpute showed better performance than the other methods with lower RMSEs and higher PCCs. The RMSE of DCA increased from 46.00 to 122.06, and the RMSE of DeepImpute increased from 59.87 to 127.41 (Supplemental Materials Table S1). From Figures 7 and 8, we can clearly see that AutoImpute achieved the highest RMSE ranging from 191.08 to 193.53 and lowest PCC, showing the worst performance. Note that the detailed RMSEs and PCCs of different methods can be found in Supplemental Materials (Supplementary Table S1). Moreover, we visualized the scatter plot for the true gene expression values and imputed gene expression values for six simulated datasets, which were shown in Supplemental Materials (Supplementary Figure S44–S49). In general, DCA and DeepImpute were outstanding among all imputation methods because the data distributions were closer to *y = x* curve.

Via the visualization and quantitively comparison results, it can be concluded that DCA and DeepImpute significantly outperform other methods for maintaining true expression distribution. Notably, both of them are deep learning-based methods, demonstrating the power of deep learning algorithms for recovering true gene expression.

### Comparison of imputation methods for cell clustering

**Figure 9.**
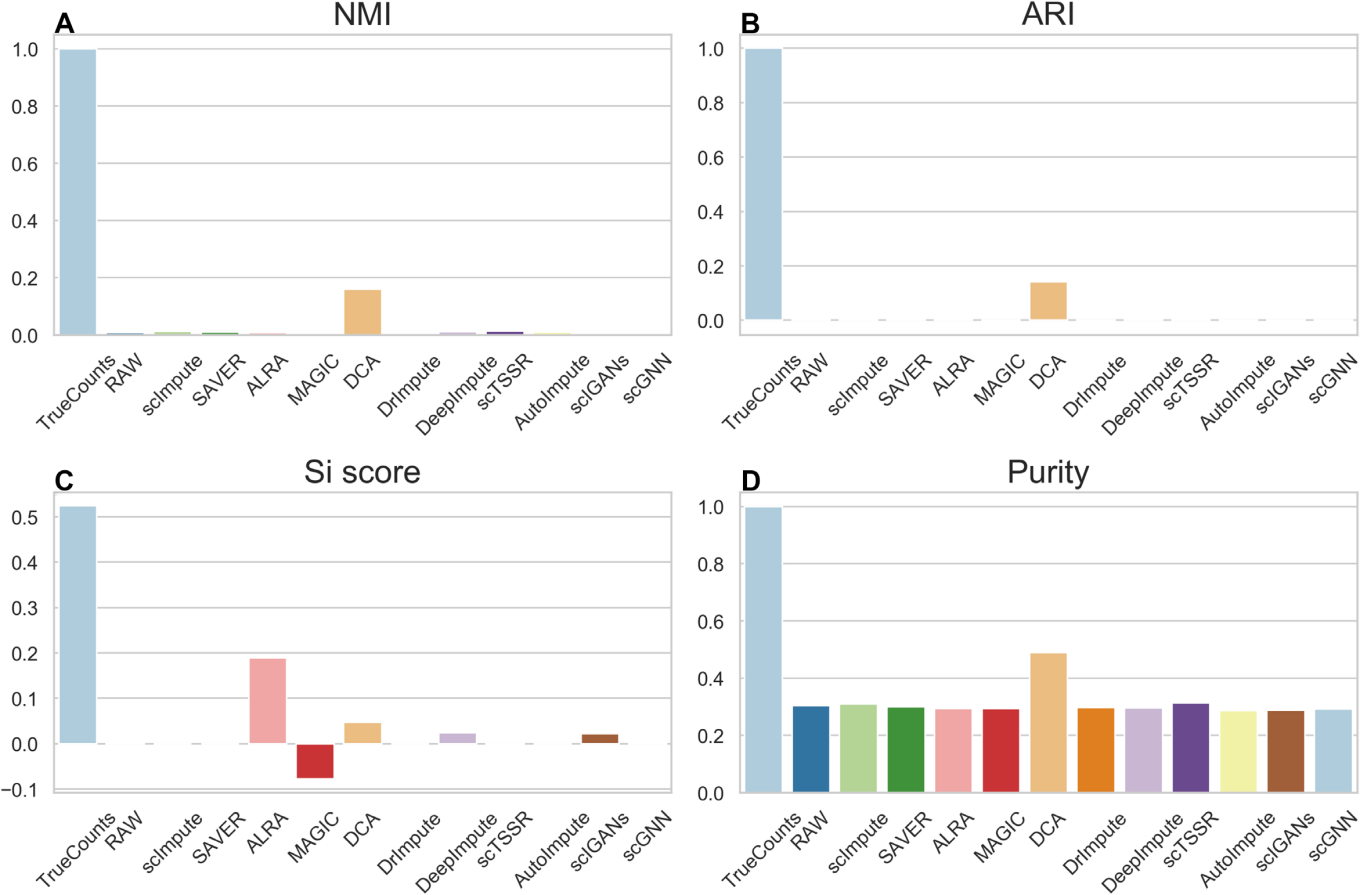
Performance evaluation of 11 imputation methods for cell clustering at dataset with zero expression rate of 0.78. (**A–D**) NMI, ARI, Si and Purity scores of the clustering results of 11 imputation methods obtained by t-SNE and *k*-means on simulated dataset.

**Figure 10.**
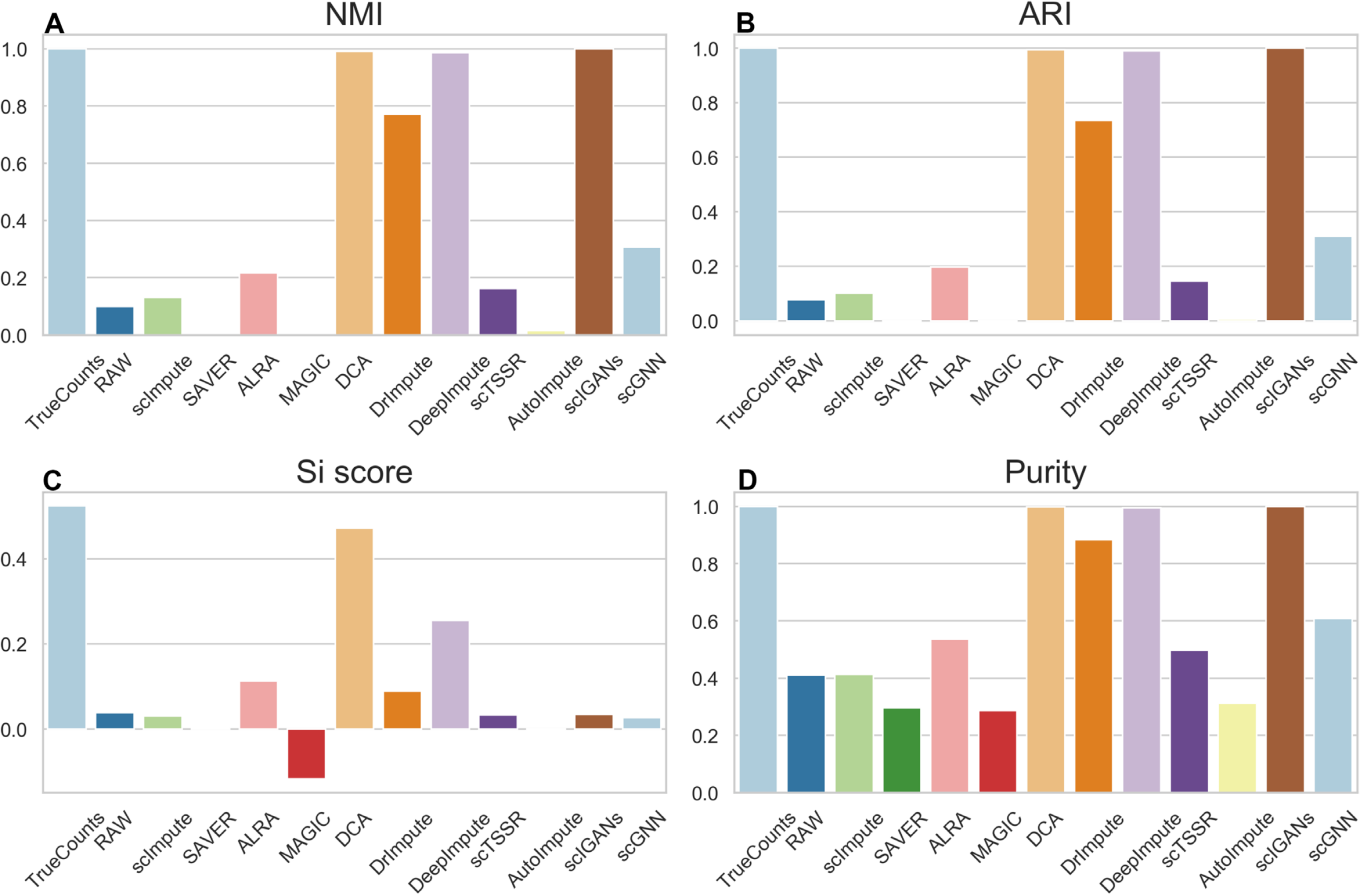
Performance evaluation of 11 imputation methods for cell clustering at dataset with zero expression rate of 0.42. (**A–D**) NMI, ARI, Si, and Purity scores of the clustering results of 11 imputation methods obtained by t-SNE and *k*-means on simulated dataset.

Recently, many clustering methods have been developed to deal with cell clustering in single-cell sequencing data (50,51). It proves that in downstream analysis, identification of cell subpopulations is a key application of scRNA-seq. However, due to the existence of ‘dropout’ events in scRNA-seq data, clustering methods like k-means cannot accurately identify cell subpopulations. Studies demonstrate that a good imputation method should make a positive contribution to cell clustering (12,52). We firstly used t-SNE to perform dimension reduction on raw count matrix and the imputed matrices derived from imputation methods, and utilized k-means algorithm for cell clustering. It was worth noting that scNPF failed to run on the simulated datasets, we only compared the other 11 methods. In this study, four metrics (NMI, ARI, Si score and Purity) were used to access and compare the clustering performance of imputation methods. Figures 9 and 10 illustrated the clustering results of dataset with different zero expression rates (0.78 and 0.42). From Figure 9, amongst the compared methods, DCA achieved parentally the highest clustering performance, giving 0.1593, 0.1409, 0.0474 and 0.4900 in terms of NMI, ARI, Si score, and Purity, respectively (Supplemental Materials Table S2). It surpassed runner-up methods (scTSSR) by 0.1454 (NMI), 0.1359 (ARI), 0.0495 (Si score) and 0.1760 (Purity), respectively. ALRA showed the highest Si score than other methods, which also improved the performance of cell clustering. In addition, DrImpute, DeepImpute, and scIGANs achieved outstanding performance when zero expression rate was 0.42 (Figure 10 and Supplemental Materials Table S3). Note that the results of different datasets were listed in Supplemental Materials (Supplementary Figure S36–S39).

**Figure 11.**
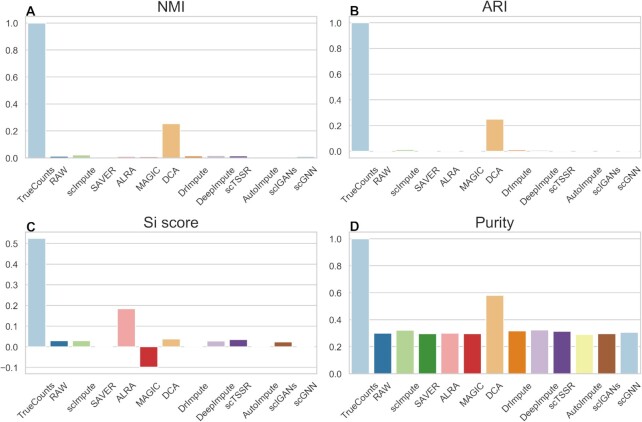
Performance evaluation of 11 imputation methods for cell clustering at dataset with zero expression rate of 0.78. (**A–D**) NMI, ARI, Si and Purity scores of the clustering results of 11 imputation methods obtained by UMAP and *k*-means on simulated dataset.

**Figure 12.**
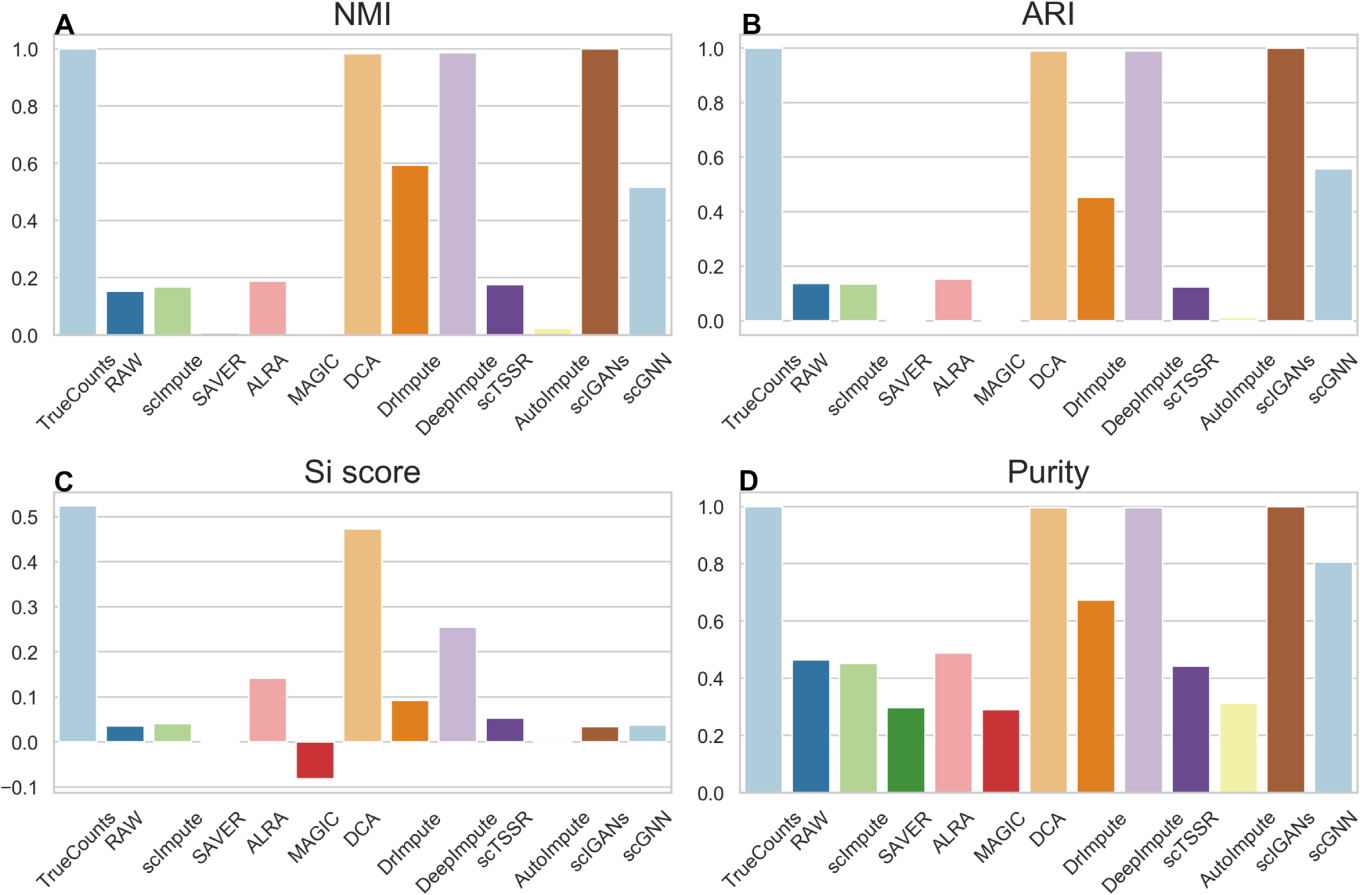
Performance evaluation of 11 imputation methods for cell clustering at dataset with zero expression rate of 0.42. (**A–D**) NMI, ARI, Si and Purity scores of the clustering results of 11 imputation methods obtained by UMAP and *k*-means on simulated dataset.

Referring to the study from Wang *et al.* (50), we found that different data preprocessing methods may affect the clustering results of single-cell RNA-seq data. To further investigate the performance of different imputation methods for cell clustering, we used UMAP for dimension reduction to carry out clustering analysis (Figures 11 and 12). We obtained similar results as the results using t-SNE as shown in Figures 9 and 10. We found that DCA achieved good performance in both datasets with zero expression rate of 0.78 and 0.42, especially when zero expression rate was 0.42 (Figures 11 and 12 and Supplemental Materials Tables S4 and S5). DrImpute, DeepImpute and scIGANs outperformed among these methods in dataset with zero expression rate of 0.42 (Figure 12 and Supplemental Materials Table S5). The NMI, ARI, Si score and Purity of DeepImpute have a clear increment by 0.9685, 0.9807, 0.2272 and 0.6720 as compared with the results of datasets with zero expression rate of 0.78 (Supplemental Materials, Tables S4 and S5). The similar results were also observed by DrImpute and scIGANs. Moreover, the NMI, ARI, Si score and Purity of different methods in different datasets can be found in Supplemental Materials (Supplementary Figures S40–S43).

In conclusion, DCA showed significant performance improvement than the other methods in cell clustering. DrImpute, DeepImpute, scIGANs and ALRA also showed an improvement when zero expression rate decreased.

### Comparison of imputation methods for gene differential expression

**Figure 13.**
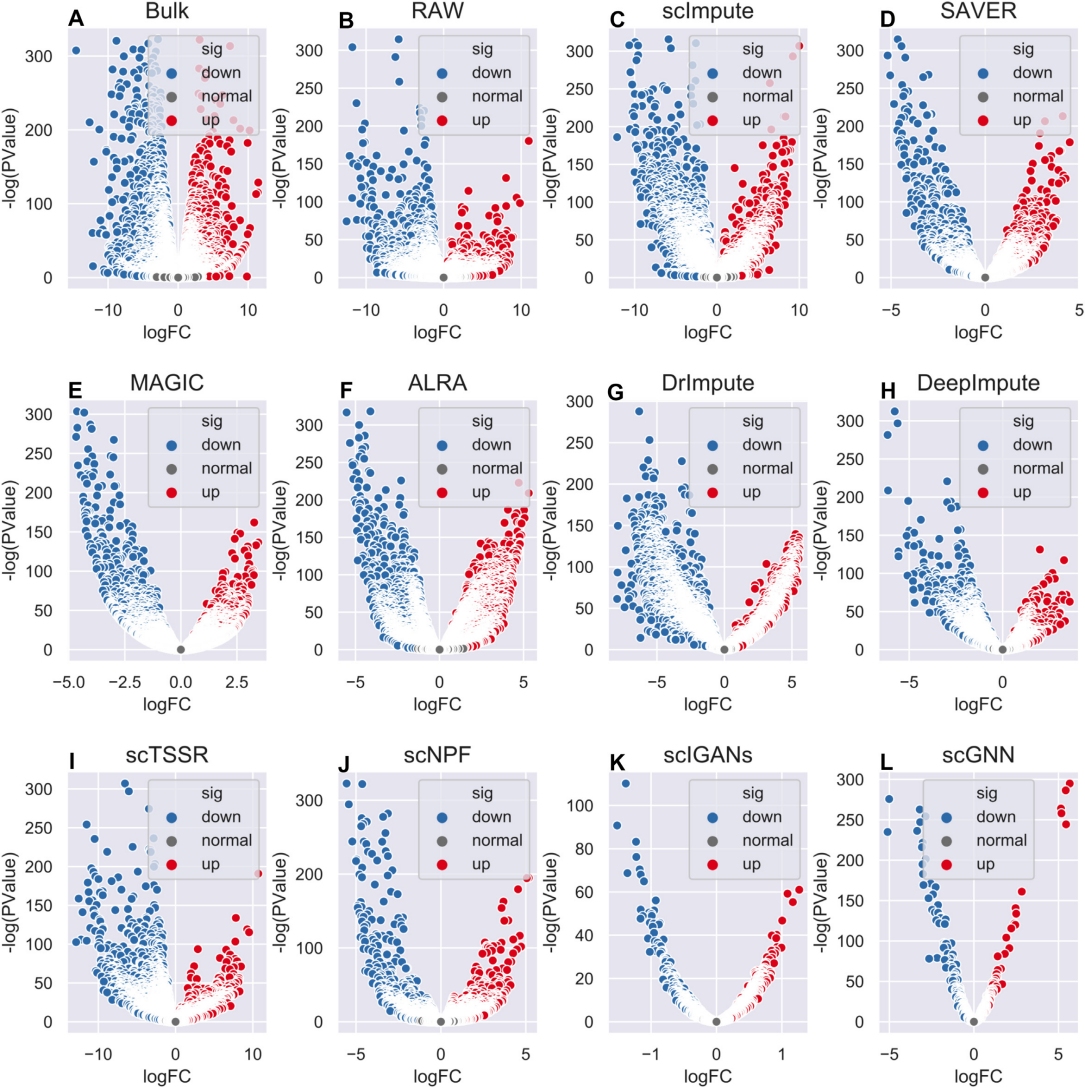
Performance of gene differential expression in different imputation methods. (**A–L**) Volcano plots of DEGs detected by bulk data, raw data, imputed data by scImpute, SAVER, MAGIC, ALRA, DrImpute, DeepImpute, scTSSR, scNPF, scIGANs, and scGNN. The *x*-axis represents $\log FC$, and the *y*-axis represents $ - \log ( {PValue} )$.

Gene differential expression analysis, as another common downstream analysis, refers to the analysis of genes whose expression levels depend on certain variables. ScRNA-seq can provide insights into the randomness of gene expressions in a single cell, while these differential expression genes impact the definition of different cell subpopulations. Therefore, an effective imputation method should preserve the consistency of scRNA-seq with bulk RNA-seq when detecting known differential genes in different cell types. Owing to the lack of golden standard of differential expression analysis, we took the differential expression genes predicted by bulk RNA-seq data as a golden reference. We investigated the performance of gene differential expression on Human Embryonic Stem Cells (ESCs) dataset. It is worth to note that DCA failed to be performed on this dataset, as the count matrix was regarded to have been normalized. A tool called edgeR (41) is commonly used to analyze gene differential expression. We ran edgeR on the raw and imputed count matrices from scRNA-seq data and the matched bulk RNA-seq data, and then visualized the results with volcano figures as illustrated in Figure 13, in which the *x-axis* represents $\log Fold\ Change$ ($\log {\rm{FC}}$), and the *y-axis* represents $ - \log {\rm{\ }}( {PValue} )$. Since edgeR failed to run on the results of AutoImpute, we only compared the performance of the rest 10 methods. The performance of capturing DEGs is defined as the overlapping between DEGs detected by bulk RNA-seq data and those detected by scRNA-seq data. From Figure 13, we can see that imputed data from scImpute detected more differentially expressed genes compared with the raw matrix. ScImpute and ALRA had the most similar shape compared with the results from bulk RNA-seq. For intuitive analysis, the numbers of differential expression genes detected by imputed data from 10 imputation methods were presented in Table 3. The raw scRNA-seq data had a much higher zero expression rate than bulk RNA-seq data, which are respectively 49.1% and 14.8%, and shared fewest DEGs with bulk RNA-seq data. As shown, we observed that the numbers of differential expression genes detected by the imputed data was larger than that detected by raw scRNA-seq data, except scGNN. This is probably due to the data preprocessing step of scGNN, which only retains the top 2000 variable genes. SAVER, scTSSR, scNPF and scIGANs captured fewer DEGs than other methods, and showed the poorest agreement with other methods. The most differential expression genes were predicted by DrImpute. In addition, the number of DEGs significantly increased compared to the number of DEGs detected by bulk RNA-seq data, especially in scImpute, ALRA, DrImpute and DeepImpute.

**Table 3.** Performance evaluation of gene differential expression in 10 imputation methods

Methods	Down expression	NotSig expression	Up expression	Differential expression
Bulk	5776	5355	5252	11028
Raw	5956	7358	3069	9025
scImpute	8231	3948	4204	12435
SAVER	3038	9323	4022	7060
MAGIC	5389	6161	4833	10222
ALRA	5965	4346	6072	12037
DrImpute	8868	3846	3890	12758
DeepImpute	7830	5322	3231	11061
scTSSR	7763	4050	1898	9661
scNPF	2265	10211	3907	6172
scIGANs	912	12528	2943	3855
scGNN	503	928	569	1072

**Figure 14.**
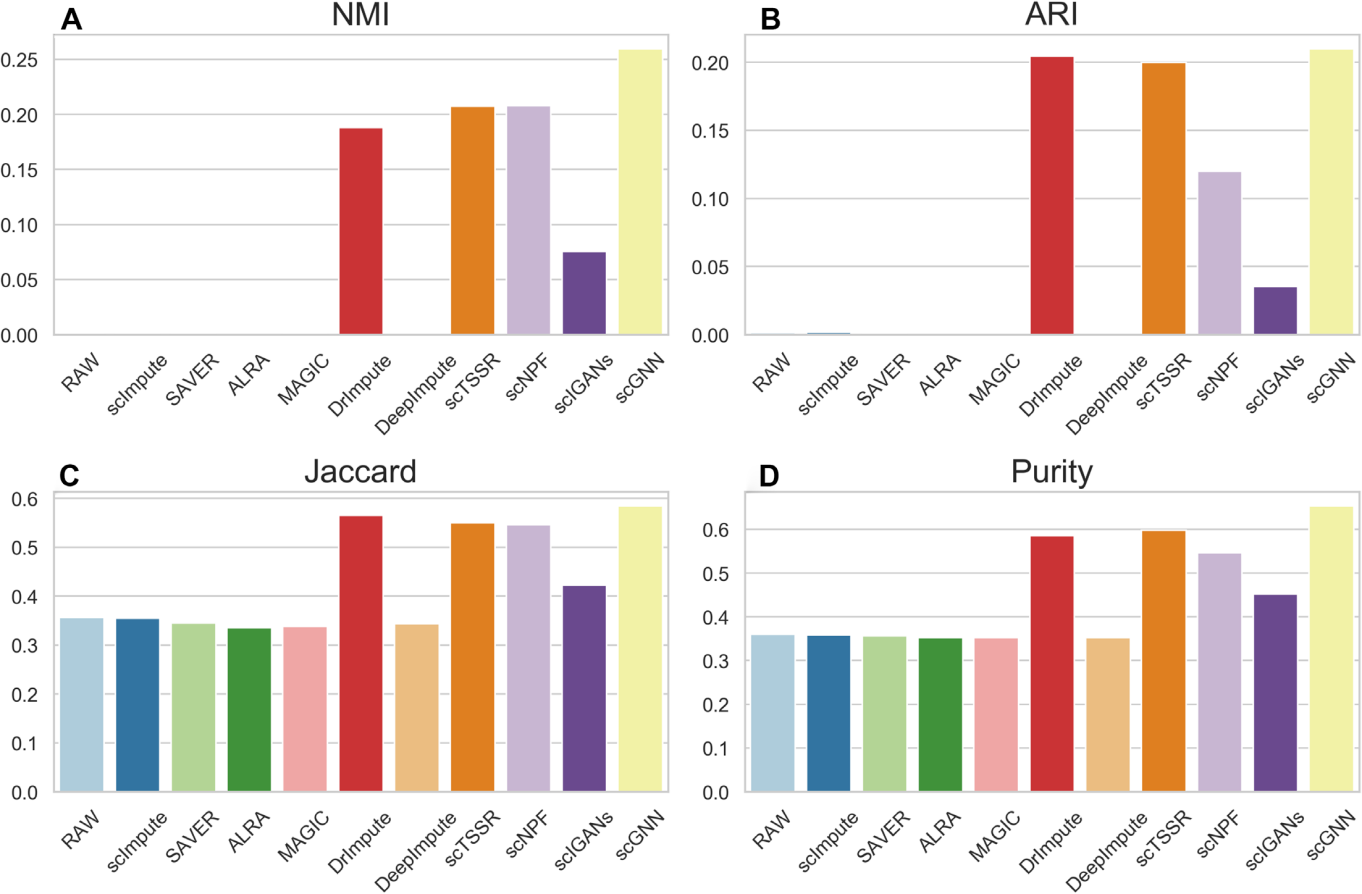
Performance evaluation of different imputation methods for gene differential expression analysis. (**A–D**) NMI, ARI, Jaccard and Purity scores of the differential expression analysis of different imputation methods obtained by edgeR on Human ESCs dataset.

Besides, we extracted the top 10 genes with highest *P*-value in bulk samples as reference for further research. Heat maps of these 10 genes in different imputed data were ploted (Supplemental Materials Figures S50–S60). Due to the gene selection strategy of AutoImpute and scGNN, only one gene overlapped with the standard gene respectively, so we did not ploted the corresponding heat maps. We found that four genes (CCDC90A, YEATS2, PPP3R1 and PSMC4) had significant differential expression in two cell types (H1 and DEC) in bulk data. SAVER, MAGIC and ALRA could modify the values of the four genes with obvious differential expression, and other methods could only identify half of them, while all genes of scIGANs showed high expression values in different cell types, which was significantly different from the results of other methods. However, we cannot confirm an imputation method's ability to detect differentially expressed genes by the number of differentially expressed genes it inferred. It is necessary to quantify how accurately the differentially expressed genes it detects compare with the gold standard. To facilitate discussion, we calculated four metrics (NMI, ARI, Jaccard and Purity) to compare the accuracy of predicting the differential expression genes. The results were shown in Figure 14. As shown, scGNN was the best method, achieving 0.2596 in terms of NMI, 0.2098 in terms of ARI, 0.5844 in terms of Jaccard, and 0.6536 in terms of Purity, respectively. Besides, DrImpute and scTSSR performed well with higher NMI, ARI, Jaccard, and Purity. Moreover, scNPF and scIGANs exceeded the average, significantly improving the performance of detecting differentially expressed genes. Note that the detailed results of different methods can be found in Supplemental Materials (Supplementary Table S6). In conclusion, scGNN, DrImpute and scTSSR are competitive methods for identifying the differential expression genes, which deserve to be recommended.

### Comparison of imputation methods for reconstructing the cellular trajectories

**Figure 15.**
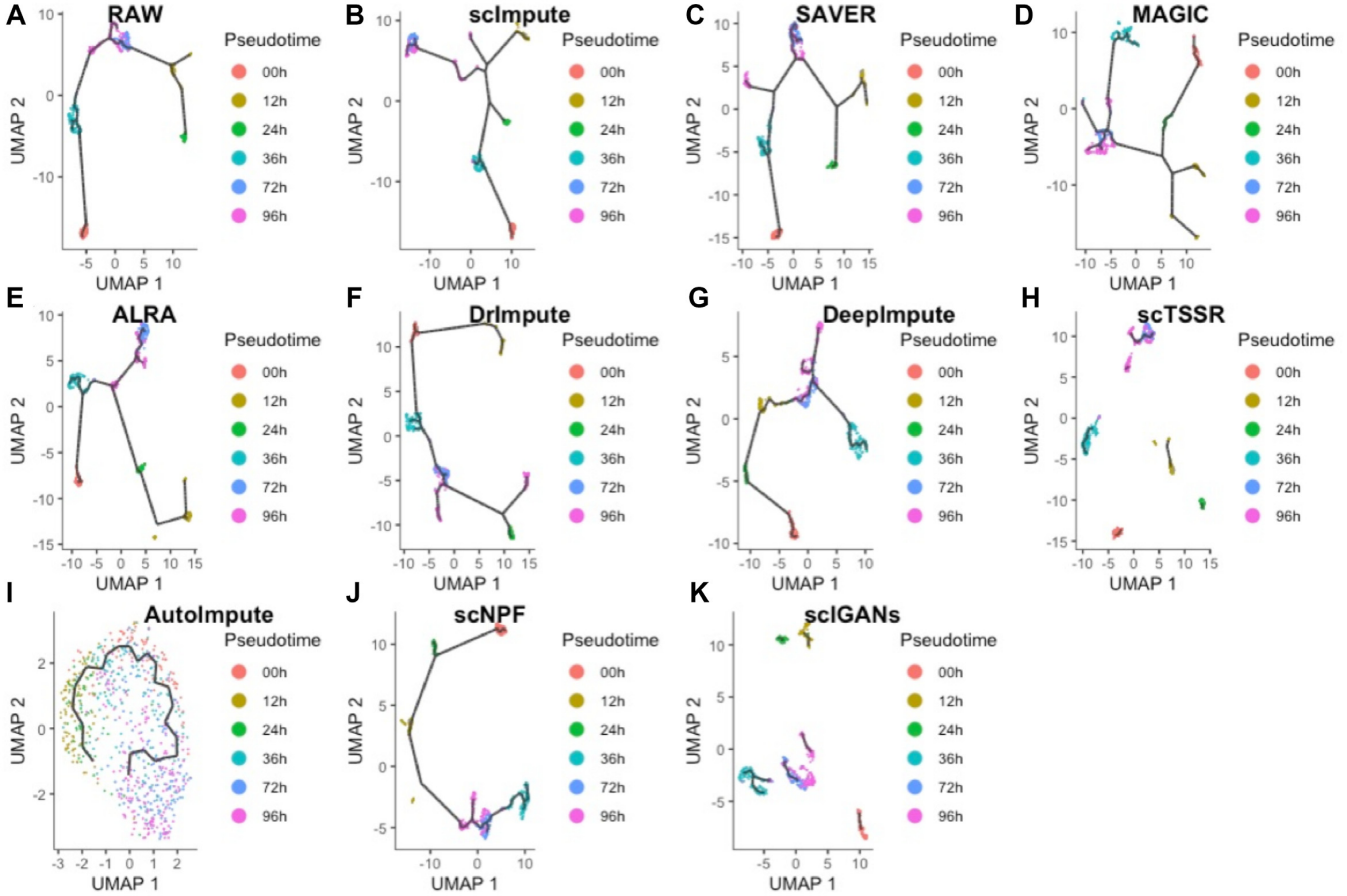
The cellular trajectories reconstructed by Monocle3 from the raw data and the imputed data obtained by different imputation methods. (**A–K**) The cellular trajectories reconstructed by raw data, scImpute, SAVER, MAGIC, ALRA, DrImpute, DeepImpute, scTSSR, AutoImpute, scNPF and scIGANs.

Reconstruction of cellular trajectories is critical to explore the pattern of cell cycle dynamics by time course in scRNA-seq data. Cellular trajectories analysis includes three main steps: dimension reducing, clustering, and trajectory reconstruction. Despite the wide use of cellular trajectories reconstruction in scRNA-seq data (53–61), it is seriously affected by ‘dropout’ events. We performed the 12 imputation methods on the raw data from Time-course scRNA-seq dataset, and used Monocle3 (42) and TSCAN (46) to reconstruct cellular trajectories. It is worth noting that DCA and scGNN failed to perform on this dataset, since the count matrix was regarded to be normalized in DCA, and the gene expression value after processing was so small to return null value in scGNN. POS and KOR scores are used to measure the correlation between the true time labels and the pseudo-time labels. In terms of its preprocessing step, TSCAN cannot perform on data imputed by SAVER, MAGIC, DeepImpute, AutoImpute, scNPF and scIGANs. As a consequence, we calculated POS and KOR scores with preprocessing step and without preprocessing step. The cellular trajectories constructed by Monocle3 were shown in Figure 15, and we also plotted the dynamic differentiation processes of two DEC signature genes (39): CER1 and HNF1B (Supplemental Materials Figures S61 and S62). POSs and KORs of different imputation methods were listed in Table 4. As can be seen in Table 4, scImpute achieved the highest correspondence between the cellular trajectory inferred by imputed data and true cell order with POS of 0.928 and KOR of 0.743, and also performed well in TSCAN without preprocessing step. Besides, DrImpute was the only method that improved the performance of TSCAN with (or without) preprocessing step in ordering cells along a trajectory by pseudo-time with higher POS (0.473 and 0.005) and KOR (0.370 and 0.011) than these of raw data. scTSSR performed well in TSCAN with preprocessing step with POS of 0.918 and KOR of 0.734. Moreover, SAVER, ALRA and scIGANs obtained lower POS and KOR scores, demonstrating that they achieved worse results. The POSs and KORs of SAVER and ALRA without preprocessing step were even negative. The results suggest that scImpute is most appropriate for exploring the cellular trajectory in scRNA-seq data.

**Table 4.** Performance evaluation of reconstructing cellular trajectories in existing imputation methods

Methods	POS (w)	KOR (w)	POS	KOR
Raw	0.372	0.255	0.877	0.662
scImpute	0.928	0.743	0.843	0.592
SAVER	–	–	–0.821	–0.607
MAGIC	–	–	0.781	0.515
ALRA	0.786	0.629	–0.867	–0.693
DrImpute	0.845	0.625	0.882	0.673
DeepImpute	–	–	0.845	0.592
scTSSR	0.918	0.734	0.778	0.491
AutoImpute	–	–	0.856	0.621
scNPF	–	–	0.87	0.636
scIGANs	–	–	0.632	0.434

*Note that POS (w) and KOR (w) represent POS and KOR scores obtained by TSCAN with preprocessing step. POS and KOR represent POS and KOR scores obtained by TSCAN without preprocessing step. In terms of its preprocessing step, TSCAN cannot perform on SAVER, MAGIC, DeepImpute, AutoImpute, scNPF and scIGANs. – represents no value.

## DISCUSSION

In this study, we comprehensively evaluated and compared a total of 12 state-of-the-art imputation methods for scRNA-seq analysis under different scenarios. We observed that every imputation method has its own advantages and disadvantages, with no outstanding method. In general, recent deep learning-based approaches exhibit better performance than model-based in most aspects. However, it's worth to note that some model-based methods achieved satisfied performance in some experiments (i.e. reconstruction of cellular trajectory), which might be due to their good ability of maintaining the relationships between cells and genes.

We firstly highlighted the advantage of imputation methods for recovering gene expression. We visualized the shape of true counts data, raw data and imputed data obtained by 12 imputation methods on six simulated datasets, and calculated RMSEs and PCCs to evaluate the performance. The results showed that DCA and DeepImpute outperformed other methods. DeepImpute used highly correlated genes of the target genes to impute the missing values, and DCA can capture the nonlinear gene-gene correlation. Moreover, the two methods tend to recover the missing values by deep learning algorithms, demonstrating that using deep learning algorithms reasonably can recover gene expression more effectively. In addition, we found scIGANs performed better when zero expression rate became lower, accounting for the application of GAN in scRNA-seq analysis. Generative adversarial network consists of a generative model and a discriminative model. Its advantage is that the parameter update of the generative model comes from the back propagation of the discriminative model. This mechanism may have improved the ability of GANs to capture some intrinsic information of scRNA-seq data.

Then, we focused on evaluating the performance of imputation methods for improving the downstream analysis, including cell clustering, gene differential expression analysis, and reconstructing cellular trajectories.

For cell clustering, we evaluated the results obtained by t-SNE and *k*-means and UMAP and k-means on six simulated datasets in terms of NMI, ARI, Si scores, and Purity. With the recovery of the missing values in terms of autoencoder, DCA focused on the distribution of scRNA-seq data. ALRA maintained the biological zeros in scRNA-seq data successfully, which was beneficial to preserve the original data distribution. Besides, while zero expression rate decreasing, DrImpute, DeepImpute, and scIGANs also showed a performance improvement. Based on the clustering results, DrImpute optimized the distribution pattern of cells from the same cluster, leading to the improvement of cell clustering. Highly correlated genes of the target genes were indispensable to impute the missing values in DeepImpute. DCA, DeepImpute, scIGANs, and ALRA belong to deep learning-based methods, demonstrating that deep learning-based methods are beneficial to the performance of cell clustering. In conclusion, similarity information in genes and cells plays an important role in cell clustering. Moreover, we also investigated the impact of different dimension reduction tools and found that the tools have similar results for cell clustering analysis.

For gene differential expression analysis, we ran edgeR on scRNA-seq data and the matched bulk RNA-seq data, and then visualized the results by a volcano figure. In addition, the number of differential expression genes detected by the compared methods is listed in Table 3. We found scGNN, a deep graph neural network based model, achieved significantly better performance than the other methods in differential expression analysis, demonstrating the power of graph neural network in effectively capturing gene similarity information. Interestingly, scGNN deployed a gene selection strategy, which retained the top 2000 variable genes for data imputation. It might also help to improve their performance in differential expression analysis. With the strength of the applicability, graph neural network is suitable for data that is relatively sparse and requires collaborative information of neighboring nodes, such as gene expression matrix. Moreover, two model-based methods, DrImpute and scTSSR, are not the best but also showed relatively good performance. It might because the similarity information of cells and genes in gene expression data used in the methods are beneficial to inferring the correlation of genes and cells, respectively.

To investigate the reconstruction of the cellular trajectories, we performed Monocle3 and TSCAN on scRNA-seq data. The results suggest that scImpute outperformed all other imputation methods, and proved scImpute showed better performance in imputing data with collinearity.

It is worth noting that, as for AutoImpute, in different aspects, its performance showed consistently worse than other methods. That is probably due to the gene selection in AutoImpute for preprocessing scRNA-seq data, AutoImpute only keeps the top 1000 high-dispersion genes for each expression data. The gene selection may significantly affect the existing gene expression structure, causing unreasonable results for downstream analysis.

In addition, we analyzed three possible reasons why deep learning-based imputation methods are better than model-based imputation methods. Firstly, the size of scRNA-seq data increased from hundreds to millions because of the widespread use of data, which caused the problem of high dimensionality. Based on the features of large sizes and high dimensionality, deep learning algorithms are more adaptable. Next, rather than recovering missing expression values, it is more important to recover data characteristics that are more meaningful for further analysis in scRNA-seq data. Therefore, deep learning-based methods are more advantageous for learning the features of data than model-based methods. Finally, deep learning algorithms are good at constructing gene-gene and cell-cell relationship networks in scRNA-seq data. With these prior information, the overall structure of data can be recovered through continuous optimization.

Most importantly, we built the first platform namely *scIMC* for comparison and visualization analysis, which would allow researchers of interest to perform the comparison analysis of all the available imputation methods on their specific scRNA-seq datasets. In particular, our platform can provide visualization comparison results for downstream analysis, and give users useful guidance to see which imputation method is more appropriate on their specific datasets.

Finally, it should be pointed out that the comparative results and corresponding conclusions in this study are all based on the datasets mentioned above, which might be not fully applicable in all situations. Those who wish to further explore the performance of imputation methods in different datasets, can use our *scIMC* to conduct the experiments.

## WEB SERVER IMPLEMENTATION

**Figure 16.**
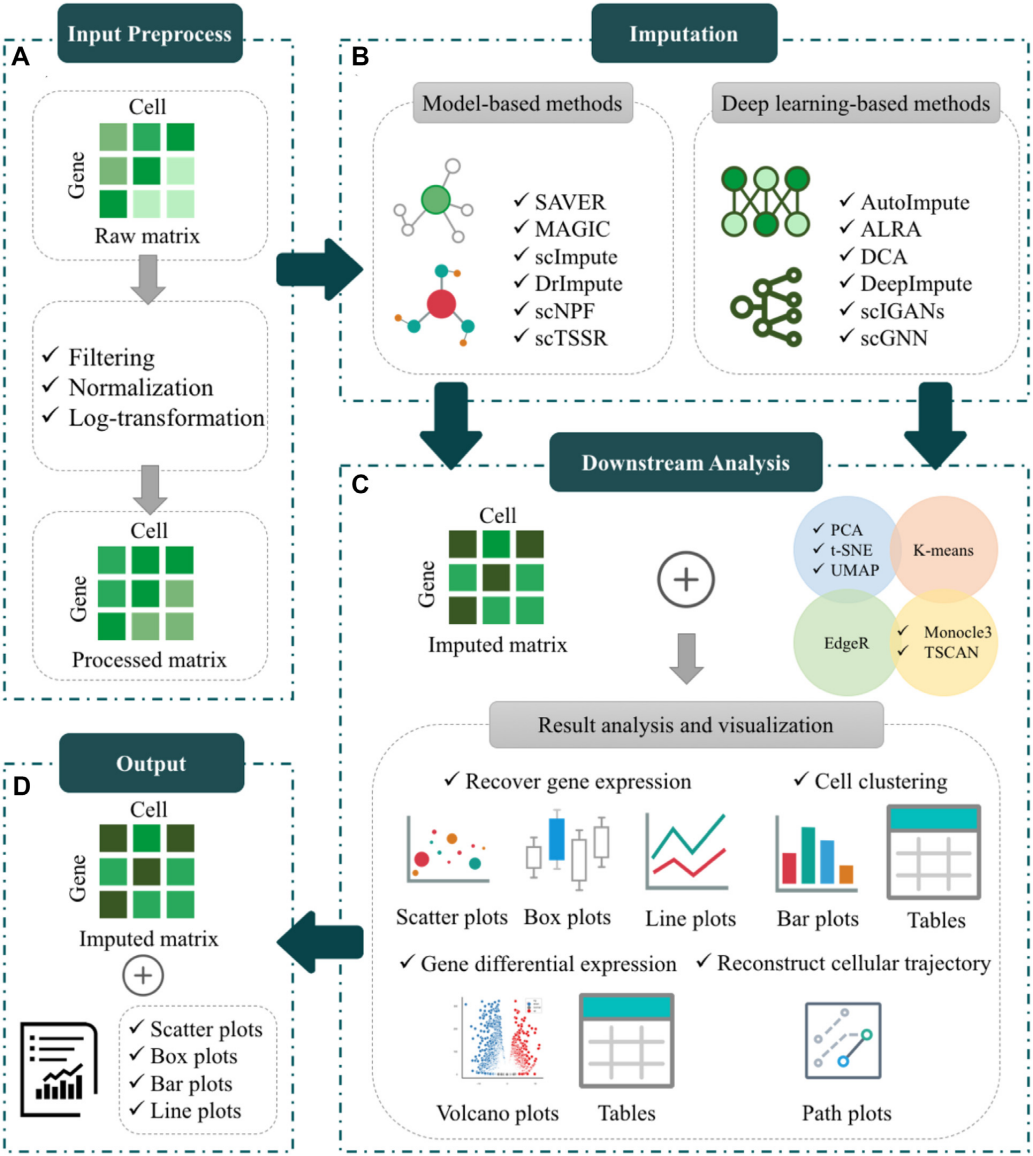
The workflow of *scIMC*. (**A**) Input Preprocess. The inputs from users are raw gene expression matrix, which will be preprocessed before imputation. The standard data preprocessing procedure contains three steps: filtering, normalization, and log-transformation. (**B**) Imputation. The processed gene expression matrix will be imputed by different imputation methods, which are divided into two categories: model-based and deep learning-based. (**C**) Downstream Analysis. In downstream analysis module, the inputs are true counts matrix (without dropouts), raw matrix, the imputed matrix and cell labels. There are four different analyses in this module: Recover gene expression, Cell clustering, Gene differential expression, and Reconstruct cellular trajectory. (**D**) Output. We provide the results of two modules: (1) imputation and (2) downstream analysis. The output of the Imputation module is the imputed gene expression matrix, while the output of the other module is data/result visualization.

We established a web server called *scIMC* (single-cell Imputation Methods Comparison platform) so as to help readers perform different imputation methods and downstream analysis (Figure 16). It is now freely accessible via https://server.wei-group.net/scIMC/, which is the first online platform that integrates all available state-of-the-art imputation methods for benchmarking comparison and visualization analysis. *scIMC* relies on cloud computing infrastructure by Ali Cloud, and is implemented by Python, supports Internet Explorer, Google Chrome, and Safari. Given the high computing costs, web servers only run up to 1GB of data.

The main functional modules of the server include: data preprocessing, gene expression matrix imputation and downstream analysis experiments (Recover gene expression, Cell clustering, Gene differential expression, and Reconstruct cellular trajectory).

### Imputation

Imputation is a common approach to recover gene expression affected by ‘dropout’ events. Users can employ a total of 12 state-of-the-art imputation methods in *Imputation* module of *scIMC*. The matrix is preprocessed as described in Data Preprocessing and then is used to be imputed by different methods (for examples, scImpute and SAVER). We perform the imputation methods with default parameters (Details described in section Comparative analysis overview) to impute gene expression matrix, in order to compare the performance of them impartially. Users can select imputation methods required and perform them on the input matrix. After submitting all the necessary datasets, *scIMC* will provide a Job ID to query the imputation results. When the imputation completes, *scIMC* will send an email to the user's mailbox (which need to be provided together with datasets), notifying to preview and download the generated results. The results of imputed matrix for different methods can be downloaded from Job List page by clicking the button ‘Details’ corresponding to Jod ID.

### Downstream analysis

In this module, there are four kinds of experiment analysis, including Recover gene expression, Cell clustering, Gene differential expression and Reconstruct cellular trajectories. To start experiments, csv-formatted files should first be uploaded as prompted: true counts matrix, raw matrix, imputed matrices (for examples, imputed by scImpute and SAVER) and the cell labels. We performed four experiments and showed their results, respectively. To summarize the performance of recovering gene expression, RMSE, PCC and scatter plots by PCA, t-SNE and UMAP are used. When it comes to cell clustering, its performance is shown by scatter plots and four metrics: NMI, ARI, Si scores and Purity. We use edgeR to detect DEGs from different gene expression matrix and evaluate the ability of them across volcano figures and numbers of DEGs detected. Cellular trajectories reconstructed are placed to reflect the performance of reconstructing the cellular trajectories for different imputation methods. The same as *Imputation* module, *scIMC* will provide a Job ID to users. When the experiments complete, *scIMC* will send an email to the user's mailbox (which need to be provided together with datasets), notifying to preview and download the generated results. The results will show on Detail page, and can be downloaded directly on this page. To display Detail page, click the button ‘Details’ corresponding to Jod ID.

Please refer to the *scIMC* website (https://server.wei-group.net/scIMC/) for more details. Users can find User Guide on *scIMC*, and we prepared a user guide video on how to use *scIMC*. It is expected to be a useful platform for researchers in this field.

## DATA AVAILABILITY

The authors declare that the data supporting the findings of this study are available within the article and its supplementary information files. Besides, the benchmarking datasets were also available for downloading at https://server.wei-group.net/scIMC/.

## Supplementary Material

gkac317_Supplemental_File
