# Supplementary material for: scIMC: a platform for benchmarking comparison and visualization analysis of scRNA-seq data imputation methods
Source: Nucleic Acids Res. 2022 May 7;50(9):4877–99. doi: 10.1093/nar/gkac317 (PMC9122610; doi:10.1093/nar/gkac317)
Supplement: gkac317_Supplemental_File [file gkac317_supplemental_file.pdf]

Supplemental Information

Literature review of state-of-the-art imputation methods

For convenience of method description, we defined raw gene expression matrix as X^{m*n}, in which the row represents *m* genes and the column represents *n* cells. $X_{ij} \in X^{m*n}$ represents the expression value of gene i in cell j, $\hat{X}$ is the imputed matrix.

Model-based imputation methods

SAVER is a method that optimizes the whole gene expression counts, using information across genes and cells to impute zeros as well as to improve the expression values for all genes. It uses a multi-gene prediction model to restore gene expressions, which is formulated as follows:

$$X_{ij} \sim Poisson\ (s_i R_{ij}) \quad (1)$$

where s_i is a cell-specific size factor, as well as R_{ij} is the true expression (without dropouts) of X_{ij}. SAVER assumes $R_{ij} \sim \mathrm{Gamma}\ (\alpha_{ij}, \beta_{ij})$, where $\widehat{\alpha_{ij}}$ and $\widehat{\beta_{ij}}$ are the estimated shape and rate parameters after maximum-likelihood, respectively. $\widehat{X_{ij}}$ is the imputed value of X_{ij}. Finally, the imputed value is as:

$$\widehat{X_{ij}} = \frac{s_i}{s_i + \widehat{\beta_{ij}}} * \frac{X_{ij\cdot}}{s_i} + \frac{\widehat{\beta_{ij}}}{s_i + \widehat{\beta_{ij}}} * \mu_{ij} \quad (2)$$

where μ_{ij} is a prediction based on the observed expression values of the informative genes in the same cell.

MAGIC applies data diffusion to share information between similar cells to optimize the gene expression matrix, and impute the missing values**.** It imputes the "dropout" events in the following four steps: (1) converting X^{m*n} to a cell-cell distance matrix $Dist$; (2) computing an affinity matrix A using $Dist$ through a Gaussian Kernel; (3)

constructing Markov affinity matrix M, representing the probability distribution of each cell transitioning to each other cell in one step; (4) data diffusion is performed by the exponentiation of M. Finally, after t iterations, the imputed matrix is computed as:

$$\hat{X} = M^t * \bar{X} \tag{3}$$

where $\bar{X}$ is the preprocessed X^{m*n}.

ScImpute can automatically identify possible "dropout" events ("false" zero values), and perform imputation only on the identified values without introducing new noise to the rest data. It first performs the preprocessing operation of normalization and log-transformation on X^{m*n}. One of advantages of scImpute is that it can detect outliers. Clustering is used to obtain the nearest neighbors of each gene to detect outliers. Instead of treating all zero values as "dropout" events, scImpute builds a statistical model to determine whether zero values are from true "dropout" events. For gene i in cell subpopulation k, its expression value is modeled as a random variable $X_i^{(k)}$, which obeys the following distribution:

$$f_{X_i^{(k)}}(x) = \lambda_i^{(k)} Gamma\left(x; \alpha_i^{(k)}, \beta_i^{(k)}\right) + \left(1 - \lambda_i^{(k)}\right) Normal\ (x; \mu_i^{(k)}, \sigma_i^{(k)}) \tag{4}$$

where $\lambda_i^{(k)}$ is dropout rate of gene i in cell subpopulation k, $\alpha_i^{(k)}, \beta_i^{(k)}$ are parameters of Gamma distribution, and $\mu_i^{(k)}, \sigma_i^{(k)}$ are parameters of Normal distribution, respectively. Afterwards, the Expectation–Maximization (EM) is used to estimate the parameters of the mixture model, which are denoted as $\widehat{\lambda_i^{(k)}}, \widehat{\alpha_i^{(k)}}, \widehat{\beta_i^{(k)}}, \widehat{\mu_i^{(k)}}$ and $\widehat{\sigma_i^{(k)}}$, respectively. Therefore, the dropout probability d_{ij} of gene i in cell j is calculated as follows:

$$d_{ij} = \frac{\widehat{\lambda_\imath^{(k)}} Gamma\left(X_{ij}; \widehat{\alpha_\imath^{(k)}}, \widehat{\beta_\imath^{(k)}}\right)}{\widehat{\lambda_\imath^{(k)}} Gamma\left(X_{ij}; \widehat{\alpha_\imath^{(k)}}, \widehat{\beta_\imath^{(k)}}\right) + \left(1 - \widehat{\lambda_\imath^{(k)}}\right) Normal\ (X_{ij}; \widehat{\mu_\imath^{(k)}}, \widehat{\sigma_\imath^{(k)}})} \tag{5}$$

For every cell b, the genes are divided into two groups: $A_b = \{a|d_{ab} \geq t\}$ represents genes in need of imputation; $B_b = \{a|d_{ab} < t\}$ represents genes having precise expression, where t is the threshold. As last, it uses B_b to impute the expression values of genes in A_b.

DrImpute identifies similar cells using clustering, and imputes the missing values by the mean of the expression values from the cells of the same cluster. Assume that there are *h* results of clusters: $C_1, C_2, \ldots, C_h$, where *Ck (1<k<h)* denotes the *k*-th result. The missing value in C_k is calculated as follows:

$$E\left(X_{ij}\middle|C_k\right) = mean\ (X_{ij}|X_{ij}\ are\ in\ the\ same\ groups\ in\ C_k) \tag{6}$$

Next, it computes $E\left(X_{ij}\middle|C_k\right)$ for each clustering result, and imputes the "dropout" event through the mean of $E\left(X_{ij}\middle|C_k\right)$. The imputed values is predicted as:

$$E\left(X_{ij}\right) = \frac{1}{h}\sum_{k=1}^{h} E\left(X_{ij}\middle|C_k\right) \tag{7}$$

scNPF adopts the network propagation approach based on random walk with restart (RWR) to optimize the count matrix, which considers both local and global topology of the interaction network. A gene-gene interaction network *G=<V, E, B>*, where V is the set of genes, as well as *E* is the set of interactions. *B* represents the set of transition probabilities, where B_{ij} is the transition probability from node *i* to node *j*. The start point is a vector P_0 of scores on genes representing the gene expression profile of a given cell. The network propagation approach based on RWR can be computed as below:

$$P_{t+1} = rP_0 + (1-r)P_tW \quad (8)$$

W is a degree-normalized adjacency matrix of the interaction network, which is constructed by the adjacency matrix *B* and a degree matrix *D*, defining as $W = BD^{-1}$. r is the trade-off between prior information and network diffusion, governing the distance that a signal is allowed to diffuse through the network during smoothing. The propagation function runs iteratively until it converges to a steady state *P*:

$$P = r(I-(1-r)W)^{-1}P_0 \quad (9)$$

By repeating the network propagation process for each cell, a higher density matrix with smooth gene expression values is generated.

scTSSR imputes the missing values considering the similarity information between genes, and the similarity information between cells. The imputed values can be predicted as:

$$\widehat{X_{ij}} = \sum_{k\neq i}\widehat{A_{ik}}X_{kj} + \sum_{h\neq j}X_{ih}\widehat{B_{hj}} + \sum_{k\neq i}\sum_{h\neq j}\widehat{A_{ik}}X_{kh}\widehat{B_{hj}} \quad (10)$$

where $\widehat{A_{ik}}$ and $\widehat{B_{hj}}$ represent the estimate of representation coefficients which can capture the similarity between genes and the similarity between cells, respectively. It uses penalized least square method to estimate the two parameters.

Deep learning-based methods

AutoImpute is designed based on deep auto-encoder network and sparse gene expression matrix. It aims to learn the inherent distribution of input data, and estimate the missing values with minimal impact on biologically low-expressed genes. The input in AutoImpute is defined as:

$$X = M \circ R \tag{11}$$

where $\circ$ is the Hadamard product, and *R* is true counts matrix (a matrix without dropouts), which we would like to recover. *M* is a binary matrix, in which the element is set to 0 when the corresponding element in *X* is 0; otherwise, the rest elements of *M* are all set to 1. In order to obtain the optimal solution to the linear inverse problem, it assumes that $\hat{X}$ is a low-rank matrix. Accordingly, $\hat{X}$ is imputed by auto-encoder:

$$\hat{X} = D\sigma(E(X)) \tag{12}$$

where σ is the sigmoid activation function used in the encoder layer, *D* is the decoder of auto-encoder, and *E* is the encoder of auto-encoder. To this end, AutoImpute restores equation (12) using deep auto-encoder network to calculate $\hat{X}$. Because $\hat{X}$ is the estimation of *R*, the loss function of AutoImpute is defined by equation (11) and equation (12):

$$Loss = \min_{E,D} ||R - D\sigma(E(X))||_O^2 + \frac{\lambda}{2}(\|E\|_F^2 + \|D\|_F^2) \tag{13}$$

where λ is the regularization coefficient, $||||_F^2$ is the Euclidean cost function, and $||||_O^2$ represents loss which is calculated only for the non-zero counts present in expression matrix.

ALRA is a method based on low-rank approximation, which applies non-negativity and correlation structure to selectively impute the missing values, leading to maintain biological zero while imputing "dropout" events. First of all, X^{m*n} is normalized, and log-transformed. Subsequently, there are three steps in ALRA for imputing the missing values. First, it uses SVD (singular vector decomposition) to calculate the approximate optimal rank matrix of the normalized expression matrix. Next, it thresholds each gene of the resulting matrix by the absolute value of the most negative entry of the gene.

Finally, the result value is rescaled so that the mean and standard deviation of the non-zero values of each gene in the result matrix match that of the original matrix, respectively.

DCA uses the negative binomial noise model with or without zero-inflation considering the count distribution, over-dispersion and sparsity of the data to impute the missing values, and captures the nonlinear gene-gene correlation. It assumes that the noise distribution obeys $ZINB(x; a, b, c) = a\delta_0(x) + (1 - a)NB(x; b, c)$, and applies the auto-encoder to train the parameters of the distribution. DCA constructs an auto-encoder network with three output layers. The architecture formulations are as follows:

$$\begin{cases} E = ReLU(\bar{X}W_E) \\ B = ReLU(EW_B) \\ D = ReLU(BW_D) \\ \hat{M} = exp(DW_b) \\ \Pi = sigmoid(DW_a) \\ \Theta = exp(DW_c) \end{cases} \tag{14}$$

where *E*, *B*, and *D* represent the encoder, bottleneck and decoder layers, respectively. $\bar{X}$ represents the matrix that is normalized and log-transformed. The three output layers of the model ($\hat{M}$, Π, Θ) correspond to the three parameters of the ZINB distribution (*a, b, c*), which are used to learn the initial distribution of the noise. The imputed matrix is generated by replacing the raw count values with the mean of the negative binomial component.

DeepImpute utilizes dropout layers and loss function of deep neural network to learn patterns in data. It divides genes into *N* random subsets, and each subset has *S* genes, which call "target genes". If the number of target genes is not a multiple of *S*, it rounds the number of genes estimated in the subset of *N*+1. For every group of target genes, a model of deep neural network is trained. The input genes of each model are

screened through two rules: a) not belonging to the target gene; b) the top five Pearson correlation coefficients with a target gene. The number of output genes is *S*. For a given cell *c*, the loss of each model is defined as:

$$Loss = \sum Y_i * (Y_i - \widehat{Y_i})^2 \quad (15)$$

where Y_i is the value of gene i in cell *c*, and $\widehat{Y_i}$ is the estimated value.

scIGANs uses a deep generative network called BEGAN to achieve the goal of imputing the missing values. Firstly, the data of scRNA-seq are normalized by the maximum read count of each cell. After that, scIGANs reshapes the count matrix into images with size of $n * n$. The pixels of the images are represented by the normalized gene expression. If the number of genes is less than $n * n$, zero values are padded. The network of scIGANs includes two parts: generator and discriminator. The generator is constructed as follows:

$$G(z, L_{a_z}; \theta) \quad (16)$$

The input *a* is defined as: $z \sim norm\,(0, 1)$ which is obtained from any noise distribution, and label $L_{a_z} \sim U\,(1, k)$, where *k* is the number of cell types. θ is the hyper-parameter of the model that needs to be trained. The discriminator of scIGANs is defined as:

$$D(X, L_{a_X}; \omega) \quad (17)$$

The input *X* represents the reshaped images, and is defined as: $X \sim P_r$, where P_r is the distribution of real data. L_{a_X} is the cell type.

For a given cell X_i that belongs to cell type *K*, scIGANs generated a candidate set A^K, and obtained the k nearest neighbors of X_i by Euclidian distance in A^k, which is

shown as X'_{iknn}. Accordingly, the impute values are calculated:

$$\hat{X}_{i,j} = \begin{cases} X_{i,j} \; if \; X_{i,j} > 0 \\ X_{iknn,j} \; others \end{cases} \tag{18}$$

ScGNN imputes the missing values via the cell-cell relationship inferred by the iteration process. The imputation model contains three regularizers from cell graph, cell types and L1 regularizer:

$$\text{cell graph}: \gamma_1 \sum (A \cdot (X - \hat{X})^2), \gamma_1 \in [0, 1]$$
$$\text{cell types}: \gamma_2 \sum (B \cdot (X - \hat{X})^2), \gamma_2 \in [0, 1]$$
$$B_{ij} = \begin{cases} 1 \;\; \text{where i and j in the same cell type} \\ 0 \;\; \text{else} \end{cases}$$
$$\text{L1}: \beta \sum |\omega| \tag{19}$$

where A is included in the adjacency matrix (N*N) from the pruned cell graph in the last iteration, and γ_1, γ_2 represent the intensities of the regularizers. L1 reduces non-zero ω terms in $\sum |\omega|$, in which β denotes a hyper-parameter. Finally, the loss function is defined as:

$$\text{Loss} = (1 - \alpha) \sum (X - \hat{X})^2 + \alpha \sum ((X - \hat{X})^2 \circ TRS) + \beta \sum |\omega| + \gamma_1 \sum (A \cdot (X - \hat{X})^2) + \gamma_2 \sum (B \cdot (X - \hat{X})^2) \tag{20}$$

TRS is k possible gene regulatory signals, corresponding to a mixture of k Gaussian distributions that $x_j \in X$ is followed. x_j represents the normalized expression values of a gene over N cells, $\mathrm{X} = \{x_1, x_2, \dots, x_N\}$.

Supplemental Tables

Table S1. RMSEs between the true counts data and the imputed data on six simulated datasets with different dropout ratios.

Methods	zr=0.42	zr=0.48	zr=0.55	zr=0.63	zr=0.71	zr=0.78
scImpute	138.77	155.58	165.41	172.69	178.75	181.80
SAVER	186.17	186.40	186.88	186.94	187.17	187.26
MAGIC	186.16	186.37	186.86	186.92	187.15	187.25
ALRA	185.41	185.39	185.72	185.98	185.71	185.97
DCA	46.00	65.94	54.97	78.26	96.84	122.06
DrImpute	106.30	130.49	146.22	158.90	170.49	177.56
DeepImpute	59.87	75.24	114.77	109.39	119.63	127.41
scTSSR	149.96	164.07	172.84	179.31	185.27	152.33
AutoImpute	191.08	191.26	191.53	192.17	192.58	193.53
scIGANs	185.78	185.97	185.94	185.49	187.13	186.79
scGNN	187.93	188.27	188.45	188.82	188.75	189.54

* In this table, zr means *zero.rate*.

Table S2. Performance evaluation for cell clustering of 11 imputation methods obtained by t-SNE and k-means in dataset with zero expression rate of 0.78.

Methods	NMI	ARI	Si score	Purity
RAW	0.0080	0.0024	0.0028	0.3040
scImpute	0.0119	0.0057	0.0002	0.3100
SAVER	0.0103	0.0031	0.0001	0.3000
MAGIC	0.0057	-0.0014	-0.0777	0.2940
ALRA	0.0088	0.0026	0.1893	0.2940
DCA	0.1593	0.1409	0.0474	0.4900
DrImpute	0.0061	-0.0003	-0.0006	0.2980
DeepImpute	0.0115	0.0030	0.0242	0.2960
scTSSR	0.0139	0.0050	-0.0021	0.3140
AutoImpute	0.0104	-0.0039	0.0000	0.2860
scIGANs	0.0062	-0.0014	0.0225	0.2880
scGNN	0.0040	-0.0023	0.0021	0.2920

Table S3. Performance evaluation for cell clustering of 11 imputation methods obtained by t-SNE and k-means in dataset with zero expression rate of 0.42.

Methods	NMI	ARI	Si score	Purity
RAW	0.1000	0.0765	0.0388	0.4100
scImpute	0.1318	0.1012	0.0303	0.4140
SAVER	0.0043	-0.0020	0.0011	0.2960
MAGIC	0.0047	-0.0024	-0.1173	0.2860
ALRA	0.2169	0.1974	0.1127	0.5360

DCA	0.9915	0.9943	0.4722	0.9980
DrImpute	0.7715	0.7354	0.0889	0.8840
DeepImpute	0.9852	0.9897	0.2546	0.9960
scTSSR	0.1628	0.1451	0.0332	0.4980
AutoImpute	0.0146	0.0065	-0.0011	0.3120
scIGANs	1.0000	1.0000	0.0339	1.0000
scGNN	0.3072	0.3095	0.0267	0.608

Table S4. Performance evaluation for cell clustering of 11 imputation methods obtained by UMAP and k-means in dataset with zero expression rate of 0.78.

Methods	NMI	ARI	Si score	Purity
RAW	0.0127	0.0051	0.0293	0.3000
scImpute	0.0215	0.0131	0.0294	0.3220
SAVER	0.0053	-0.0011	0.0003	0.2960
MAGIC	0.0090	0.0004	-0.0989	0.2960
ALRA	0.0113	0.0035	0.1838	0.3000
DCA	0.2542	0.2483	0.0378	0.5800
DrImpute	0.0139	0.0093	0.0038	0.3160
DeepImpute	0.0167	0.0090	0.0274	0.3240
scTSSR	0.0149	0.0058	0.0344	0.3140
AutoImpute	0.0062	-0.0005	0.0007	0.2900
scIGANs	0.0050	-0.0019	0.0234	0.2960
scGNN	0.0116	0.0046	-0.0002	0.3060

Table S5. Performance evaluation for cell clustering of 11 imputation methods obtained by UMAP and k-means in dataset with zero expression rate of 0.42.

Methods	NMI	ARI	Si score	Purity
RAW	0.1530	0.1373	0.0357	0.4640
scImpute	0.1677	0.1353	0.0409	0.4520
SAVER	0.0077	0.0012	0.0030	0.2980
MAGIC	0.0052	-0.0023	-0.0822	0.2900
ALRA	0.1887	0.1528	0.1410	0.4880
DCA	0.9832	0.9893	0.4729	0.9960
DrImpute	0.5933	0.4537	0.0920	0.6740
DeepImpute	0.9852	0.9897	0.2546	0.9960
scTSSR	0.1757	0.1234	0.0530	0.4420
AutoImpute	0.0237	0.0123	-0.0005	0.3120
scIGANs	1.0000	1.0000	0.0339	1.0000
scGNN	0.5167	0.5576	0.0371	0.8060

Table S6. Performance evaluation for gene differential expression analysis of 10 imputation methods.

Methods	NMI	ARI	Jaccard	Purity

RAW	0.0015	0.0015	0.3565	0.3601
scImpute	0.0011	0.0018	0.3546	0.3581
SAVER	0.0011	0.0004	0.3455	0.3563
MAGIC	0.0003	0.0002	0.3376	0.3526
ALRA	0.0001	0.0001	0.3355	0.3526
DrImpute	0.1880	0.2042	0.5654	0.5852
DeepImpute	0.0001	0.0002	0.3439	0.3526
scTSSR	0.2076	0.1998	0.5495	0.5978
scNPF	0.2077	0.1199	0.5459	0.5459
scIGANs	0.0754	0.0353	0.4222	0.4521
scGNN	0.2596	0.2098	0.5844	0.6536

Supplemental Figures

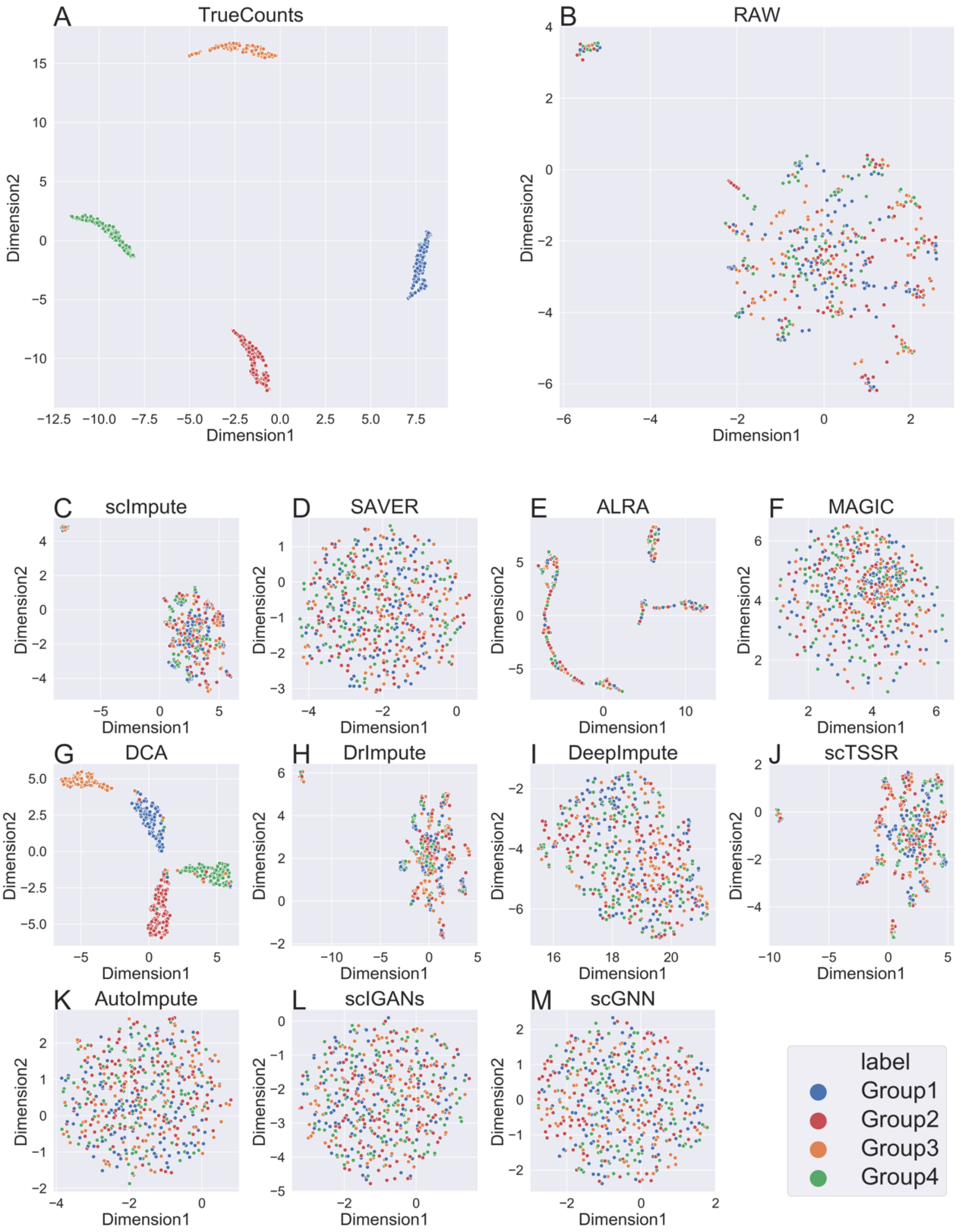

Figure S1. UMAP plots of gene expression distribution of 11 methods on simulated dataset with zero expression rate of 0.71. (A-M) UMAP plots on true counts matrix (without dropouts), raw gene expression matrix (with dropouts), and imputed matrices by scImpute, SAVER, ALRA, MAGIC, DCA, DrImpute, DeepImpute, scTSSR, AutoImpute, scIGANs, and scGNN, respectively.

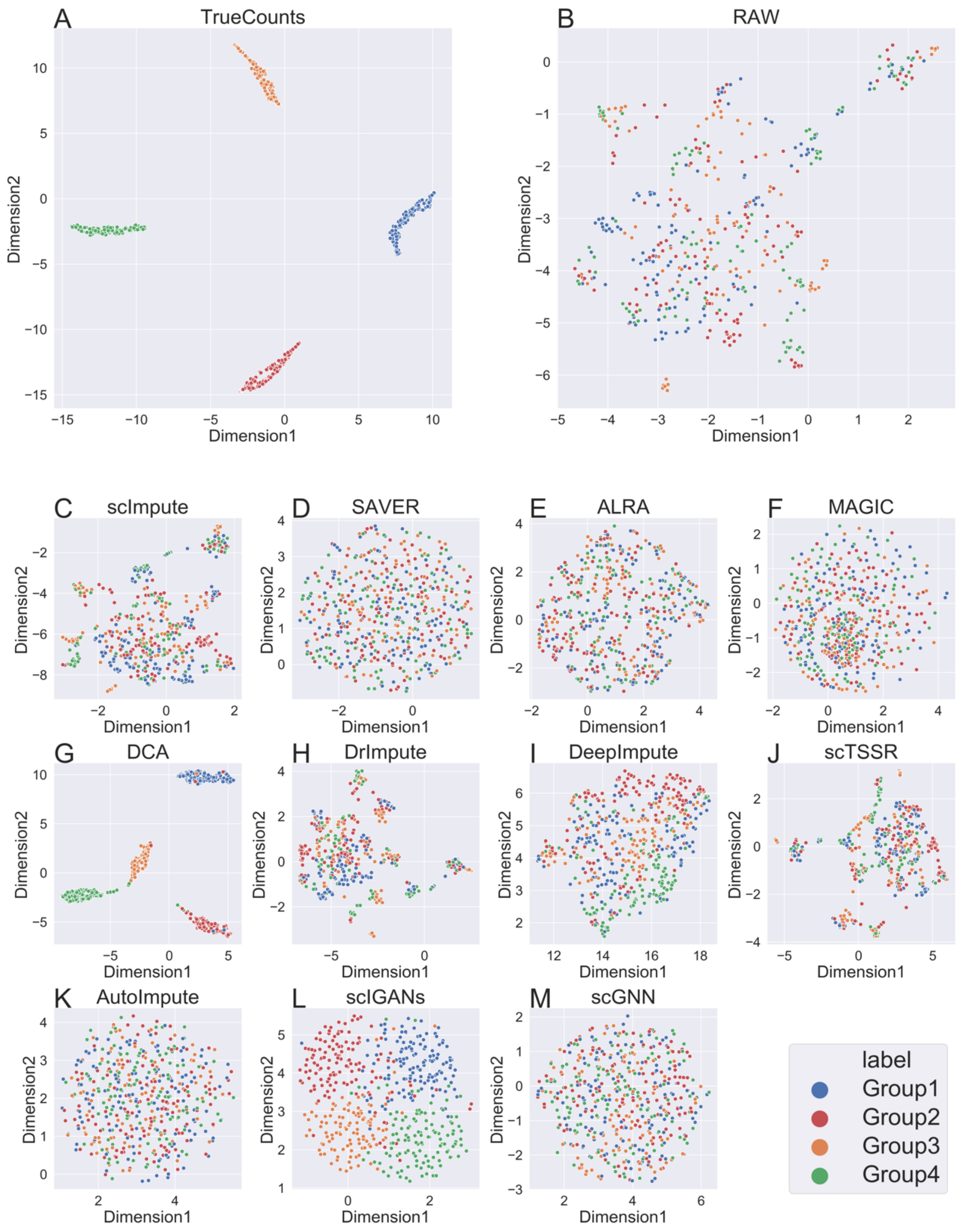

Figure S2. UMAP plots of gene expression distribution of 11 methods on simulated dataset with zero expression rate of 0.63. (A-M) UMAP plots on true counts matrix (without dropouts), raw gene expression matrix (with dropouts), and imputed matrices by scImpute, SAVER, ALRA, MAGIC, DCA, DrImpute, DeepImpute, scTSSR, AutoImpute, scIGANs, and scGNN, respectively.

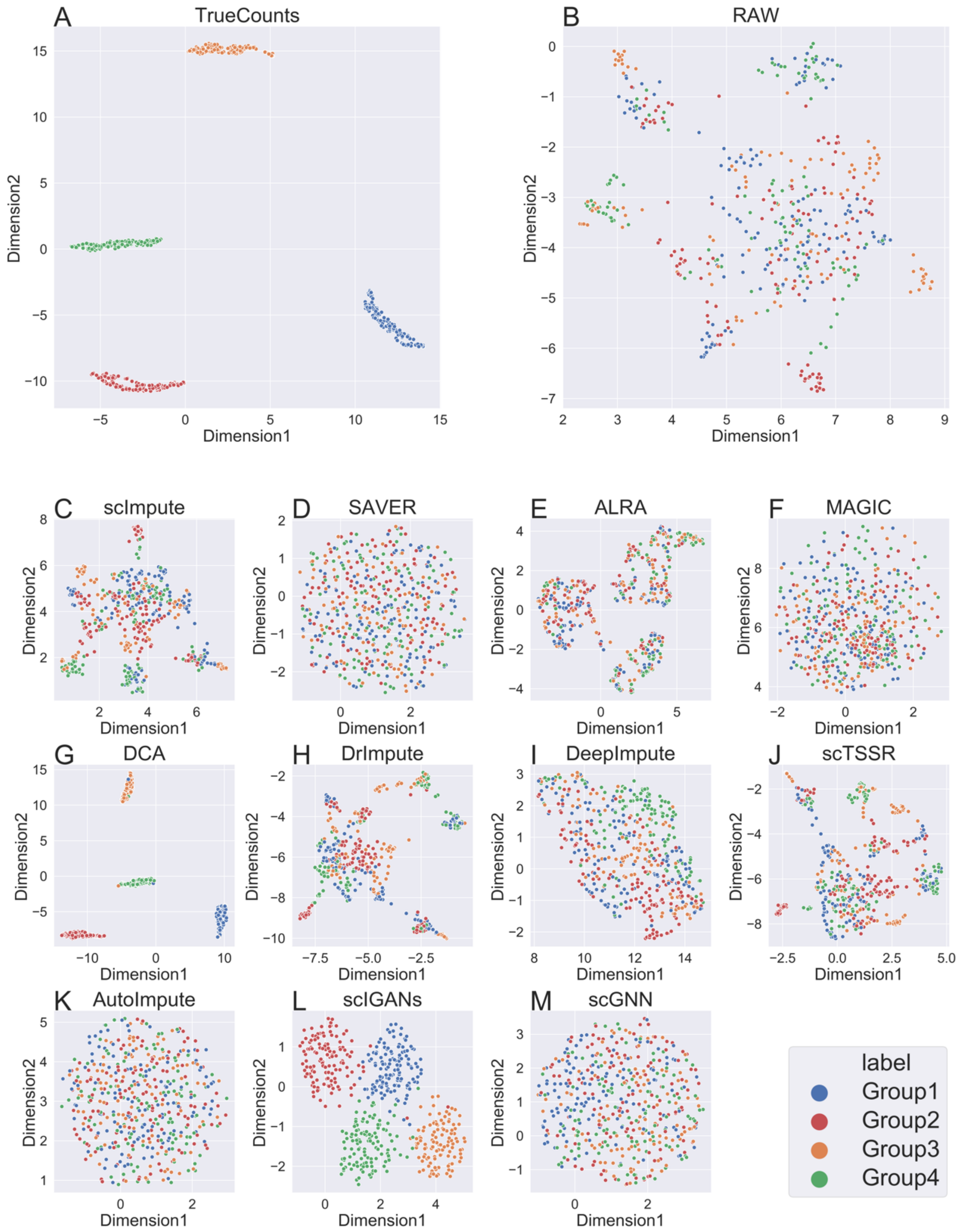

Figure S3. UMAP plots of gene expression distribution of 11 methods on simulated dataset with zero expression rate of 0.55. (A-M) UMAP plots on true counts matrix (without dropouts), raw gene expression matrix (with dropouts), and imputed matrices by scImpute, SAVER, ALRA, MAGIC, DCA, DrImpute, DeepImpute, scTSSR, AutoImpute, scIGANs, and scGNN, respectively.

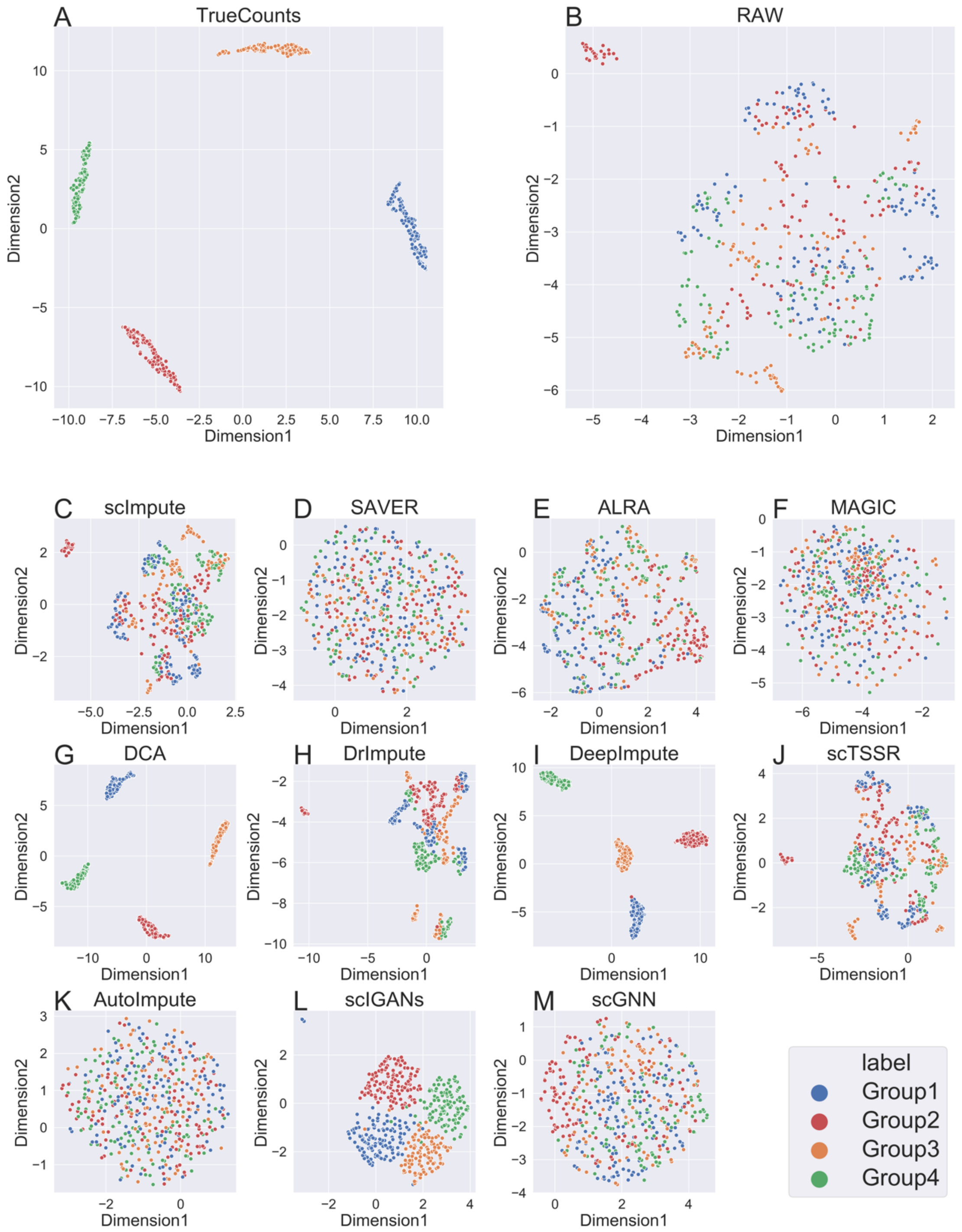

Figure S4. UMAP plots of gene expression distribution of 11 methods on simulated dataset with zero expression rate of 0.48. (A-M) UMAP plots on true counts matrix (without dropouts), raw gene expression matrix (with dropouts), and imputed matrices by scImpute, SAVER, ALRA, MAGIC, DCA, DrImpute, DeepImpute, scTSSR, AutoImpute, scIGANs, and scGNN, respectively.

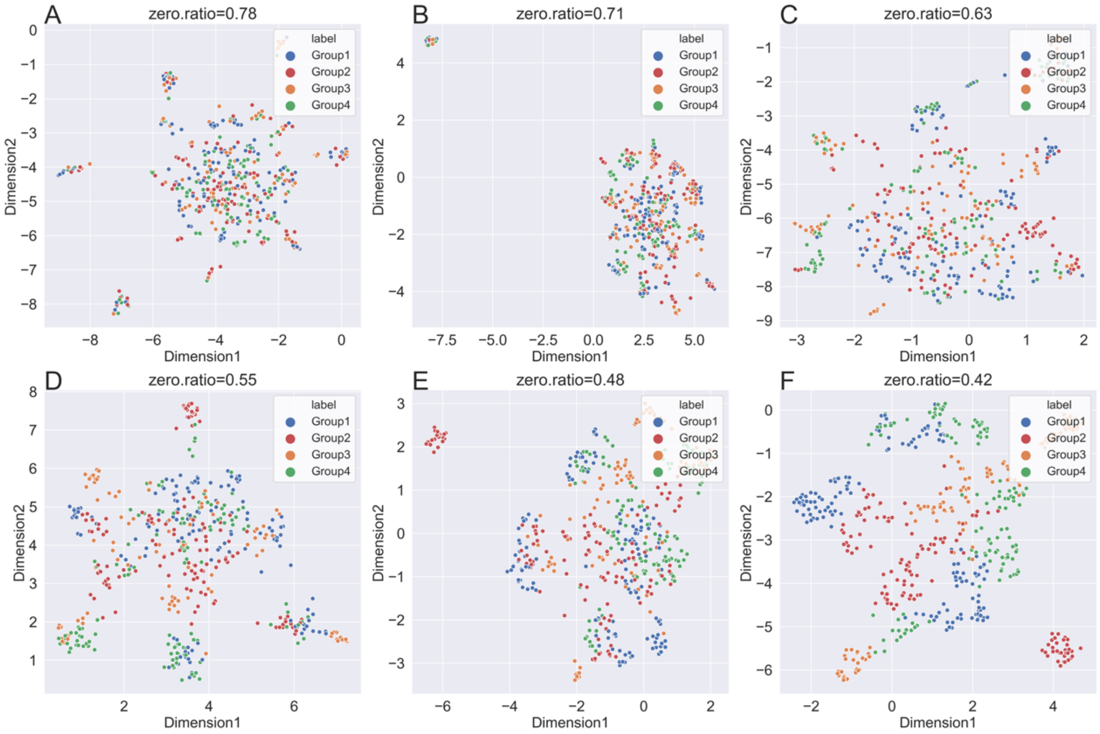

Figure S5. UMAP plots of imputed data for scImpute in six simulated datasets with different zero expression rates. (A-F) UMAP plots on simulated dataset with zero expression rate of 0.78, 0.71, 0.63, 0.55, 0.48, and 0.42.

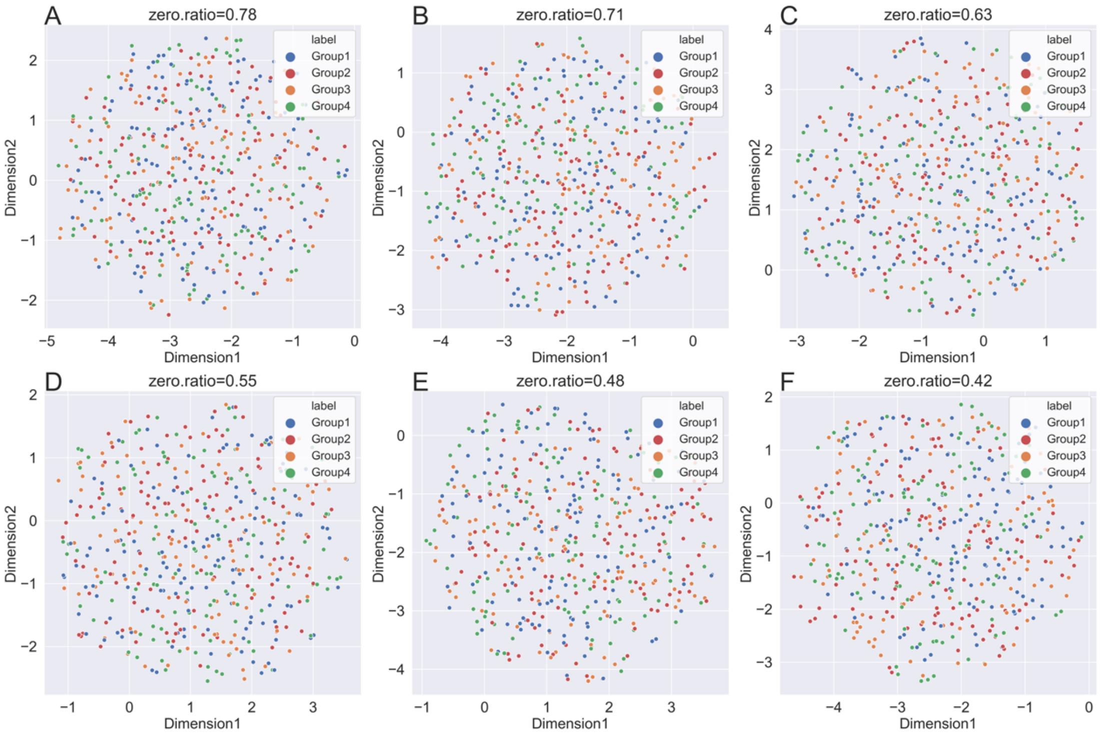

Figure S6. UMAP plots of imputed data for SAVER in six simulated datasets with different zero expression rates. (A-F) UMAP plots on simulated dataset with zero expression rate of 0.78, 0.71, 0.63, 0.55, 0.48, and 0.42.

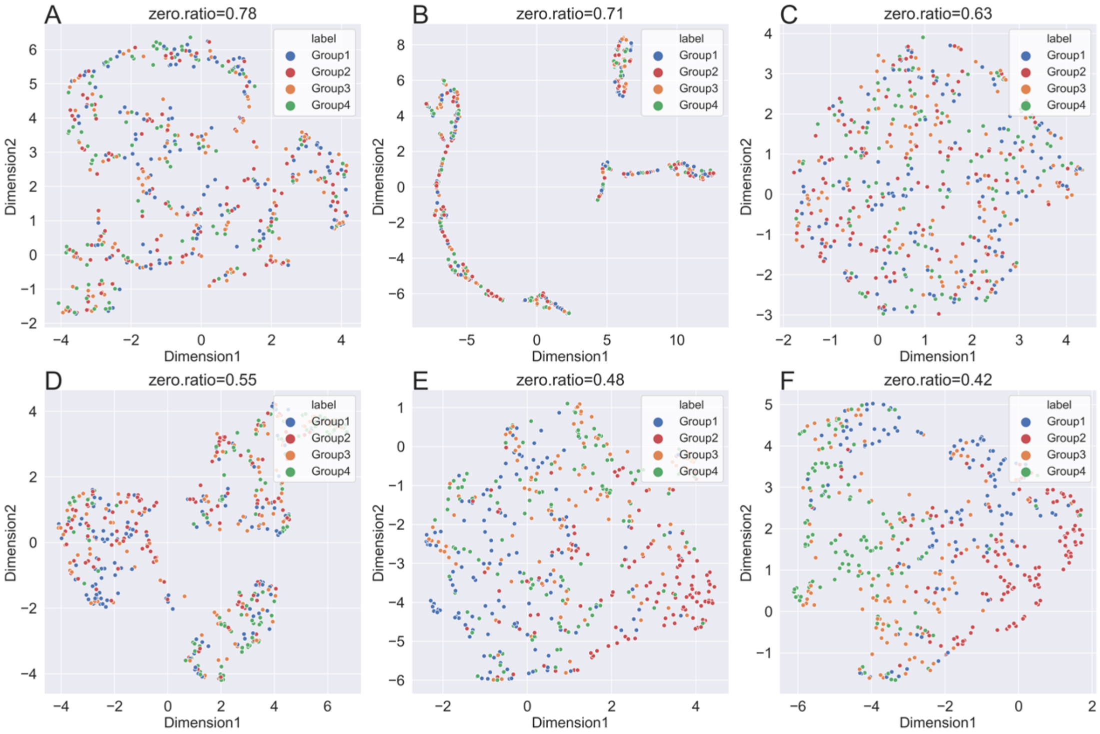

Figure S7. UMAP plots of imputed data for ALRA in six simulated datasets with different zero expression rates. (A-F) UMAP plots on simulated dataset with zero expression rate of 0.78, 0.71, 0.63, 0.55, 0.48, and 0.42.

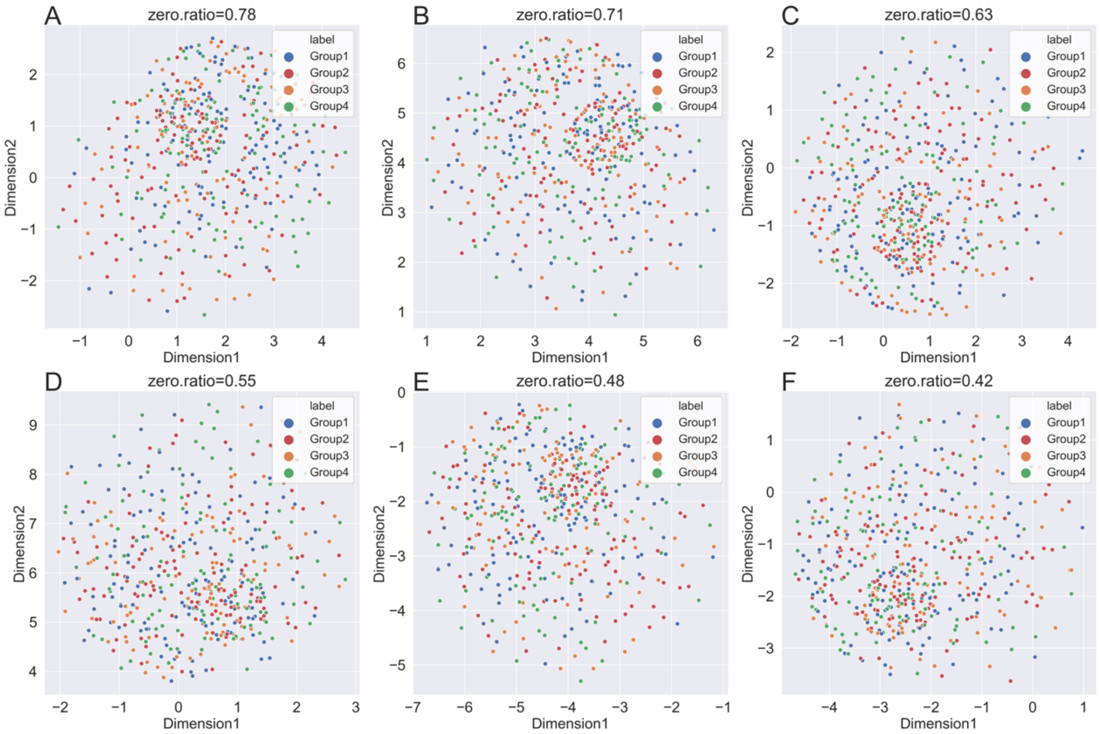

Figure S8. UMAP plots of imputed data for MAGIC in six simulated datasets with different zero expression rates. (A-F) UMAP plots on simulated dataset with zero expression rate of 0.78, 0.71, 0.63, 0.55, 0.48, and 0.42.

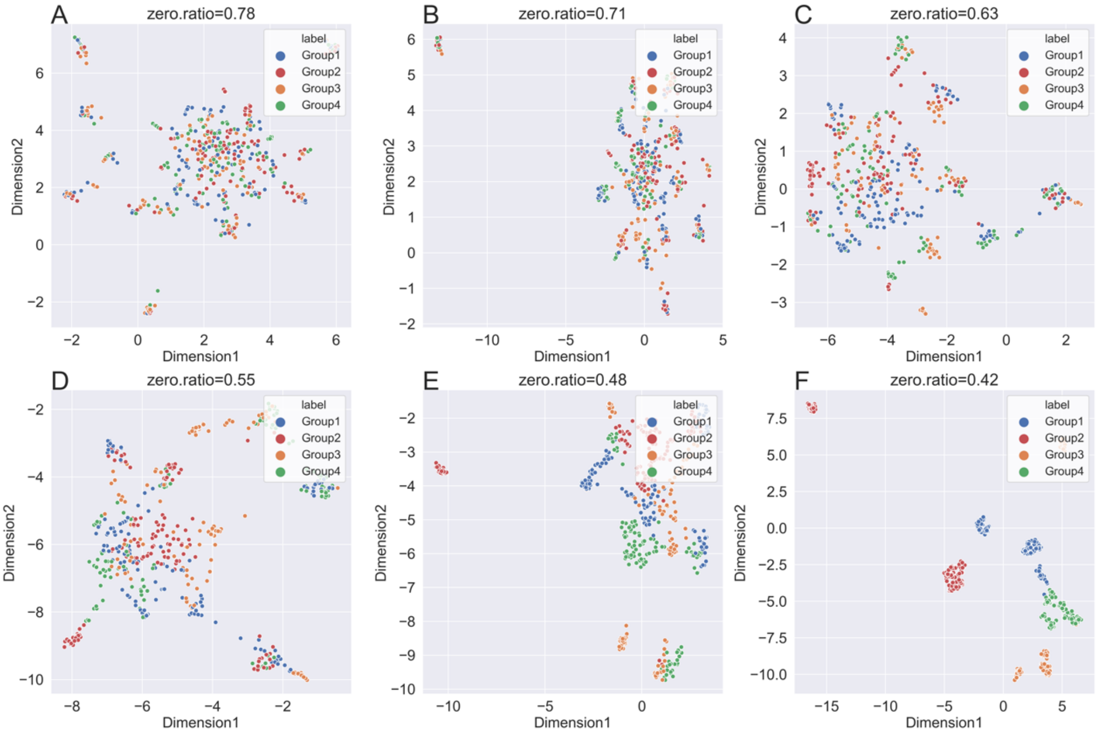

Figure S9. UMAP plots of imputed data for DrImpute in six simulated datasets with different zero expression rates. (A-F) UMAP plots on simulated dataset with zero expression rate of 0.78, 0.71, 0.63, 0.55, 0.48, and 0.42.

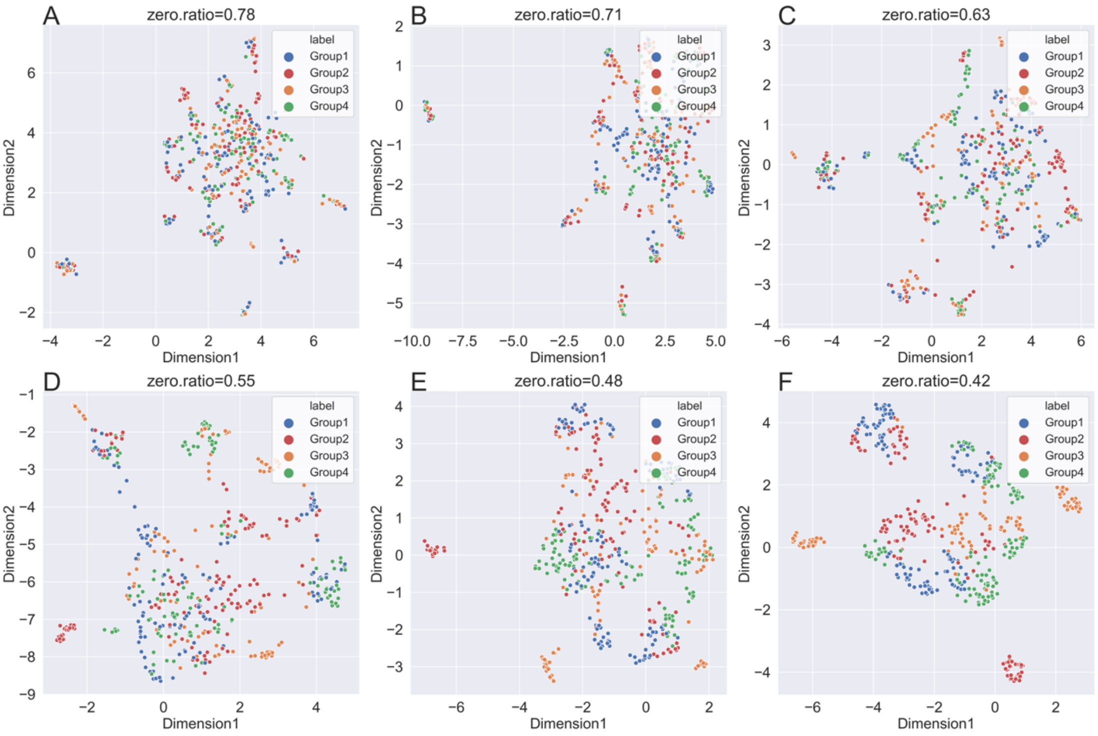

Figure S10. UMAP plots of imputed data for scTSSR in six simulated datasets with different zero expression rates. (A-F) UMAP plots on simulated dataset with zero expression rate of 0.78, 0.71, 0.63, 0.55, 0.48, and 0.42.

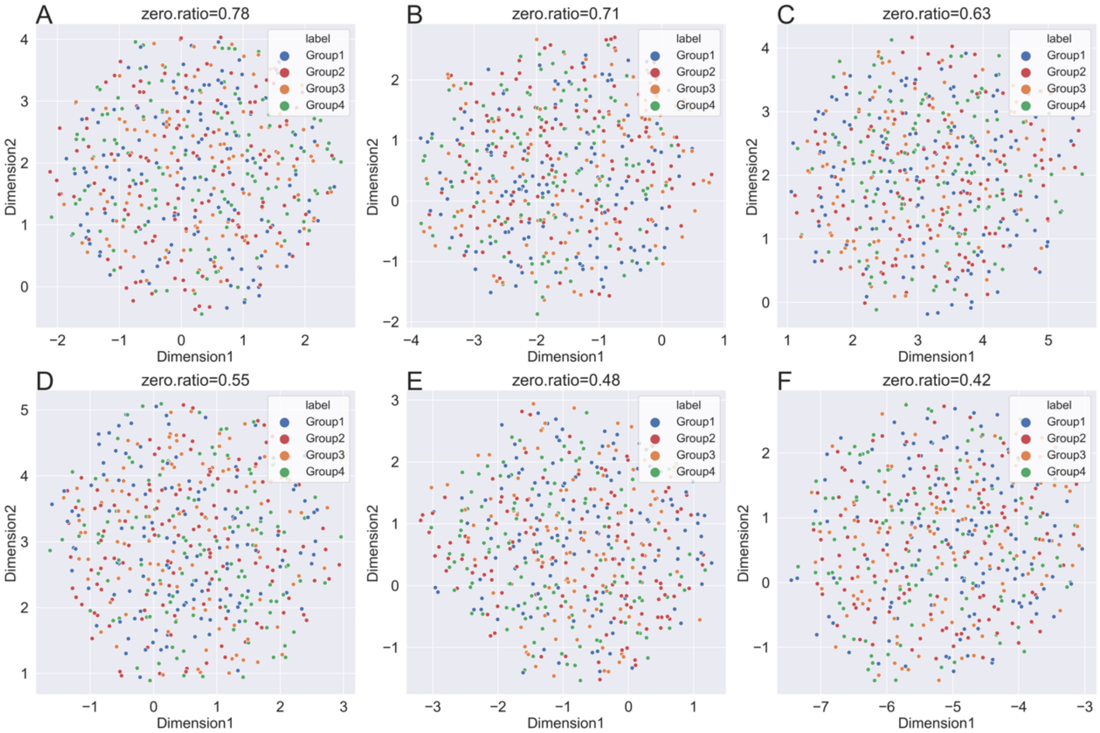

Figure S11. UMAP plots of imputed data for AutoImpute in six simulated datasets with different zero expression rates. (A-F) UMAP plots on simulated dataset with zero expression rate of 0.78, 0.71, 0.63, 0.55, 0.48, and 0.42.

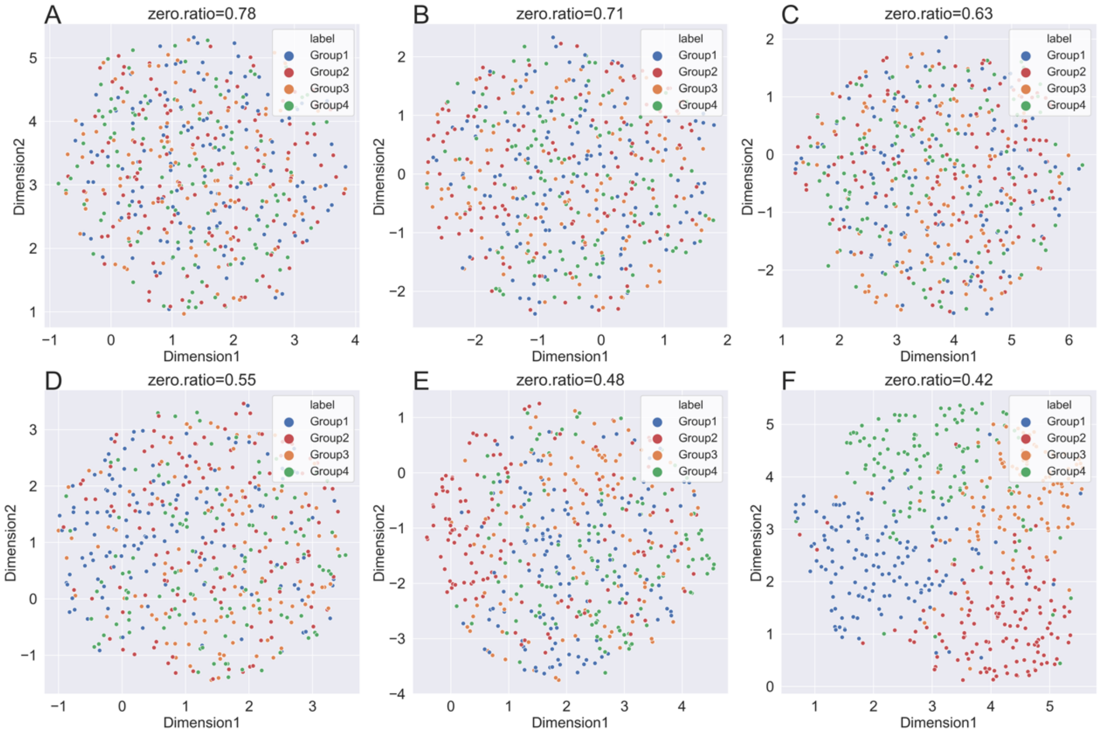

Figure S12. UMAP plots of imputed data for scGNN in six simulated datasets with different zero expression rates. (A-F) UMAP plots on simulated dataset with zero expression rate of 0.78, 0.71, 0.63, 0.55, 0.48, and 0.42.

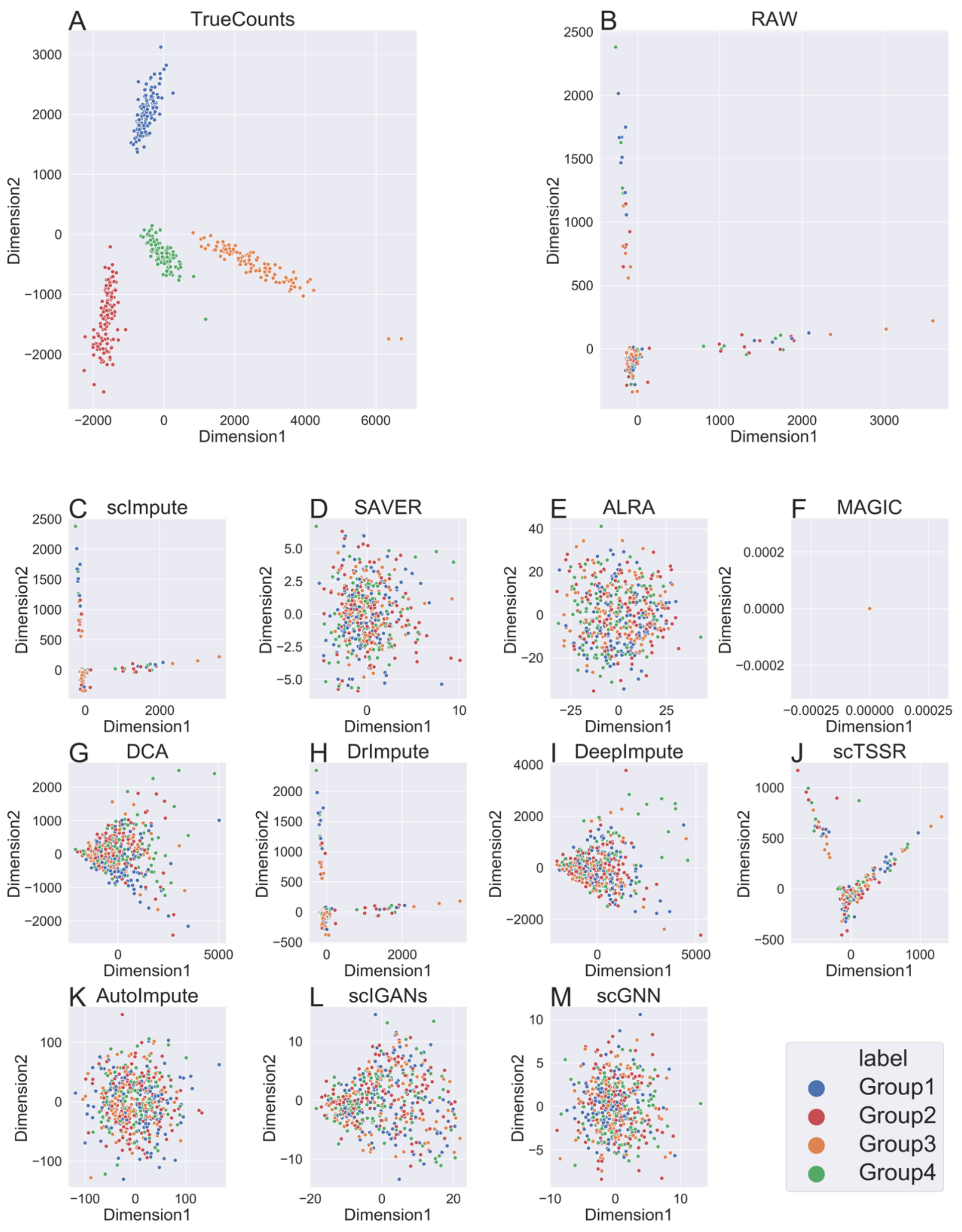

Figure S13. PCA plots of gene expression distribution of 11 methods on simulated dataset with zero expression rate of 0.78. (A-M) PCA plots on true counts matrix (without dropouts), raw gene expression matrix (with dropouts), and imputed matrices by scImpute, SAVER, ALRA, MAGIC, DCA, DrImpute, DeepImpute, scTSSR, AutoImpute, scIGANs, and scGNN, respectively.

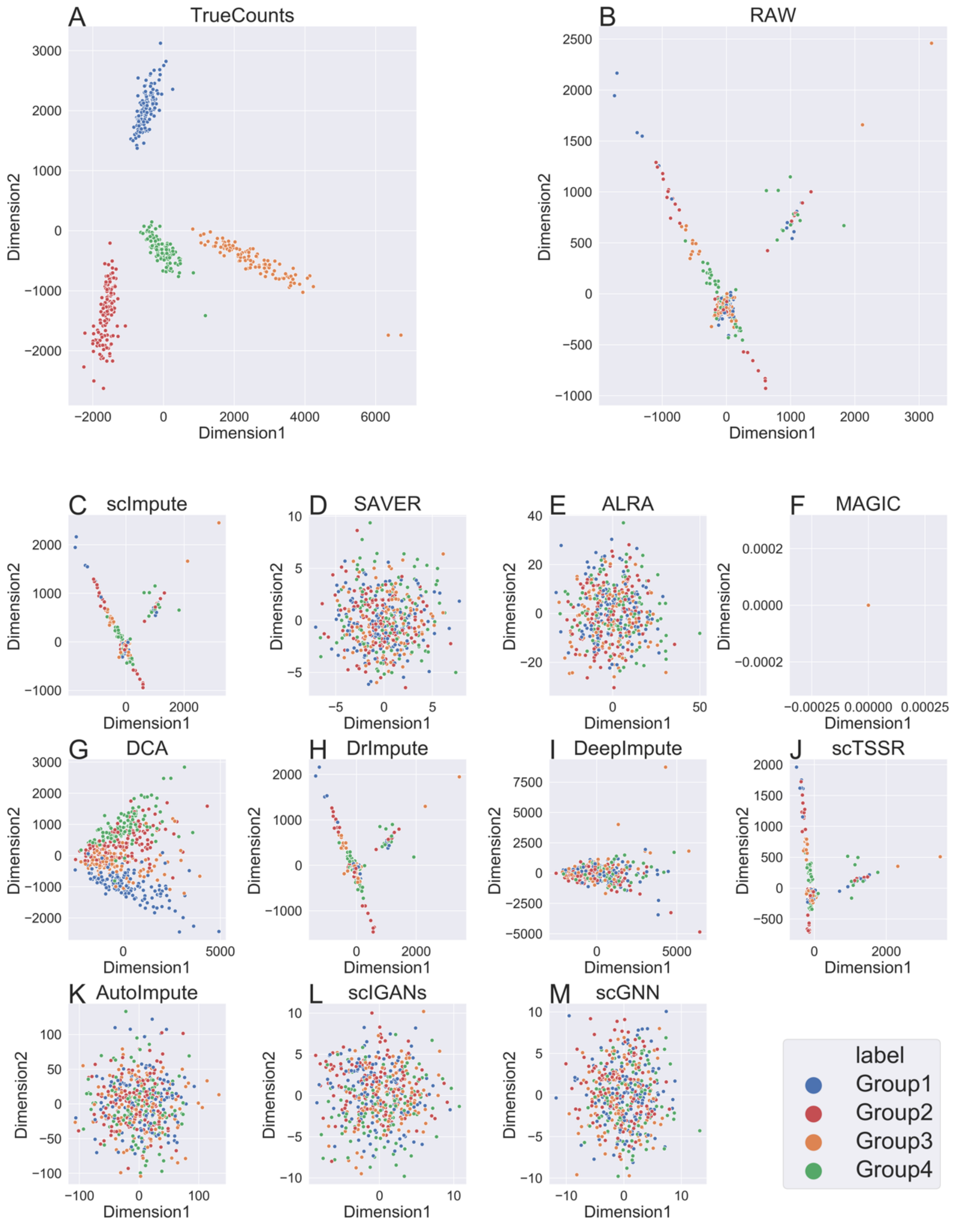

Figure S14. PCA plots of gene expression distribution of 11 methods on simulated dataset with zero expression rate of 0.71. (A-M) PCA plots on true counts matrix (without dropouts), raw gene expression matrix (with dropouts), and imputed matrices by scImpute, SAVER, ALRA, MAGIC, DCA, DrImpute, DeepImpute, scTSSR, AutoImpute, scIGANs, and scGNN, respectively.

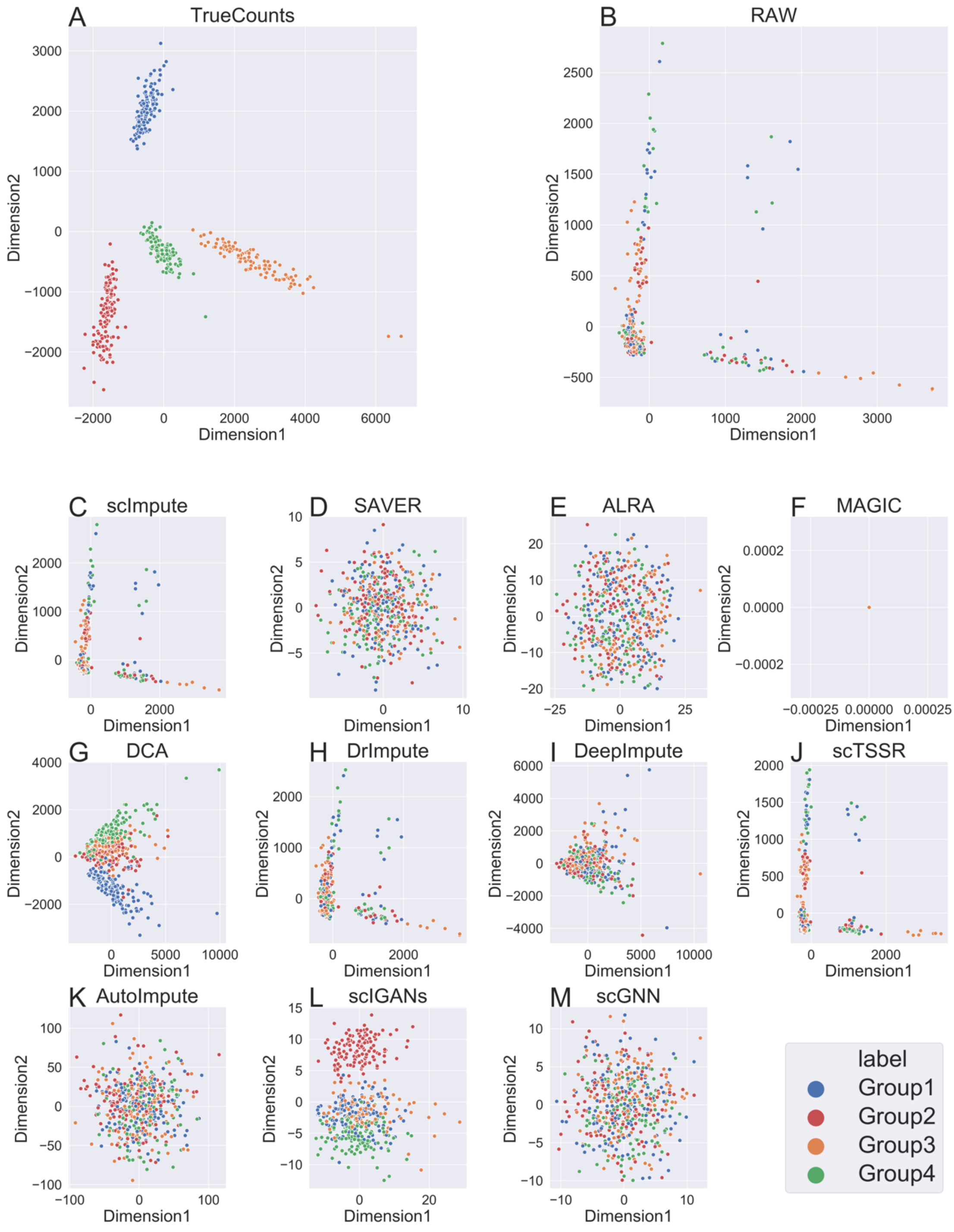

Figure S15. PCA plots of gene expression distribution of 11 methods on simulated dataset with zero expression rate of 0.63. (A-M) PCA plots on true counts matrix (without dropouts), raw gene expression matrix (with dropouts), and imputed matrices by scImpute, SAVER, ALRA, MAGIC, DCA, DrImpute, DeepImpute, scTSSR, AutoImpute, scIGANs, and scGNN, respectively.

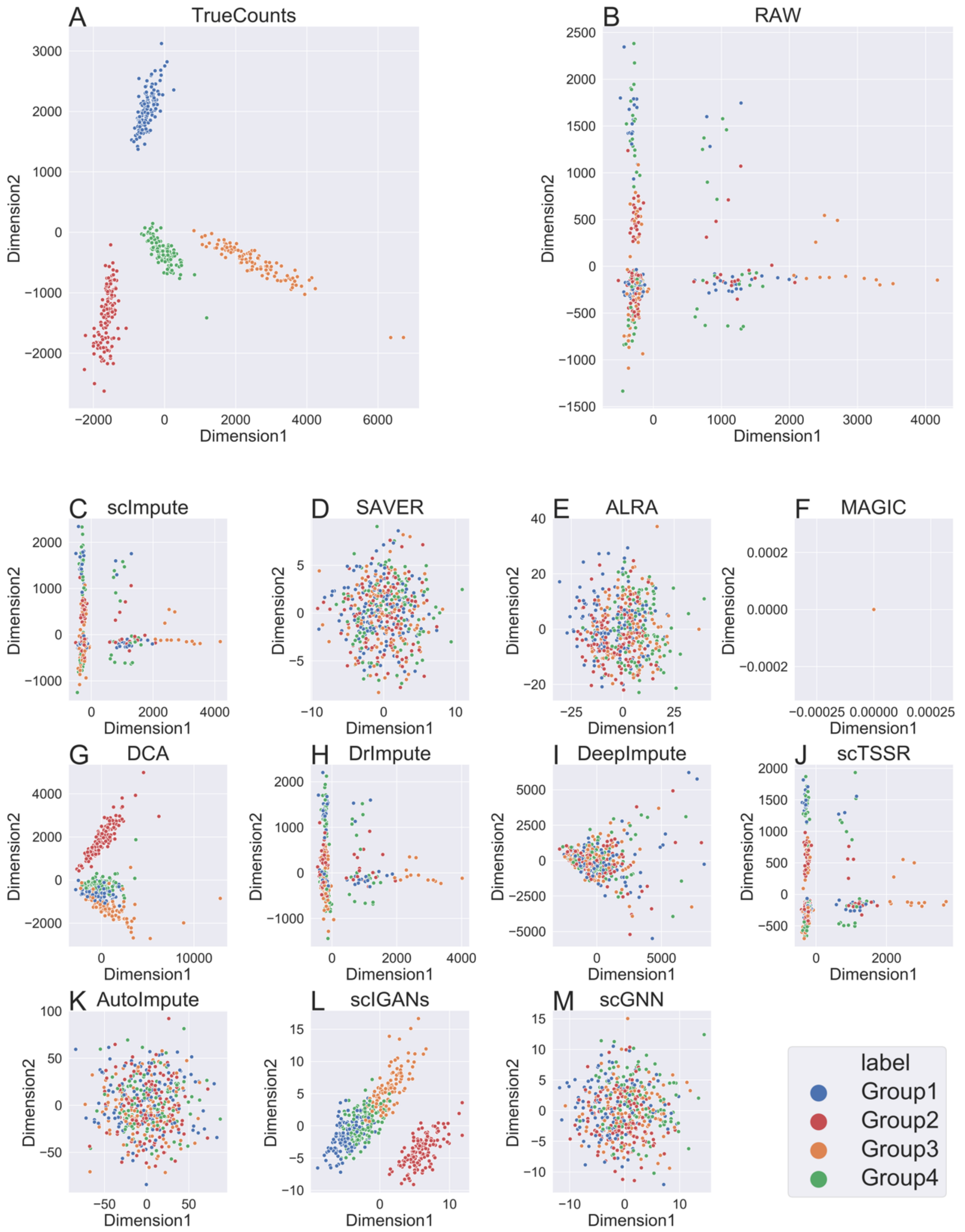

Figure S16. PCA plots of gene expression distribution of 11 methods on simulated dataset with zero expression rate of 0.55. (A-M) PCA plots on true counts matrix (without dropouts), raw gene expression matrix (with dropouts), and imputed matrices by scImpute, SAVER, ALRA, MAGIC, DCA, DrImpute, DeepImpute, scTSSR, AutoImpute, scIGANs, and scGNN, respectively.

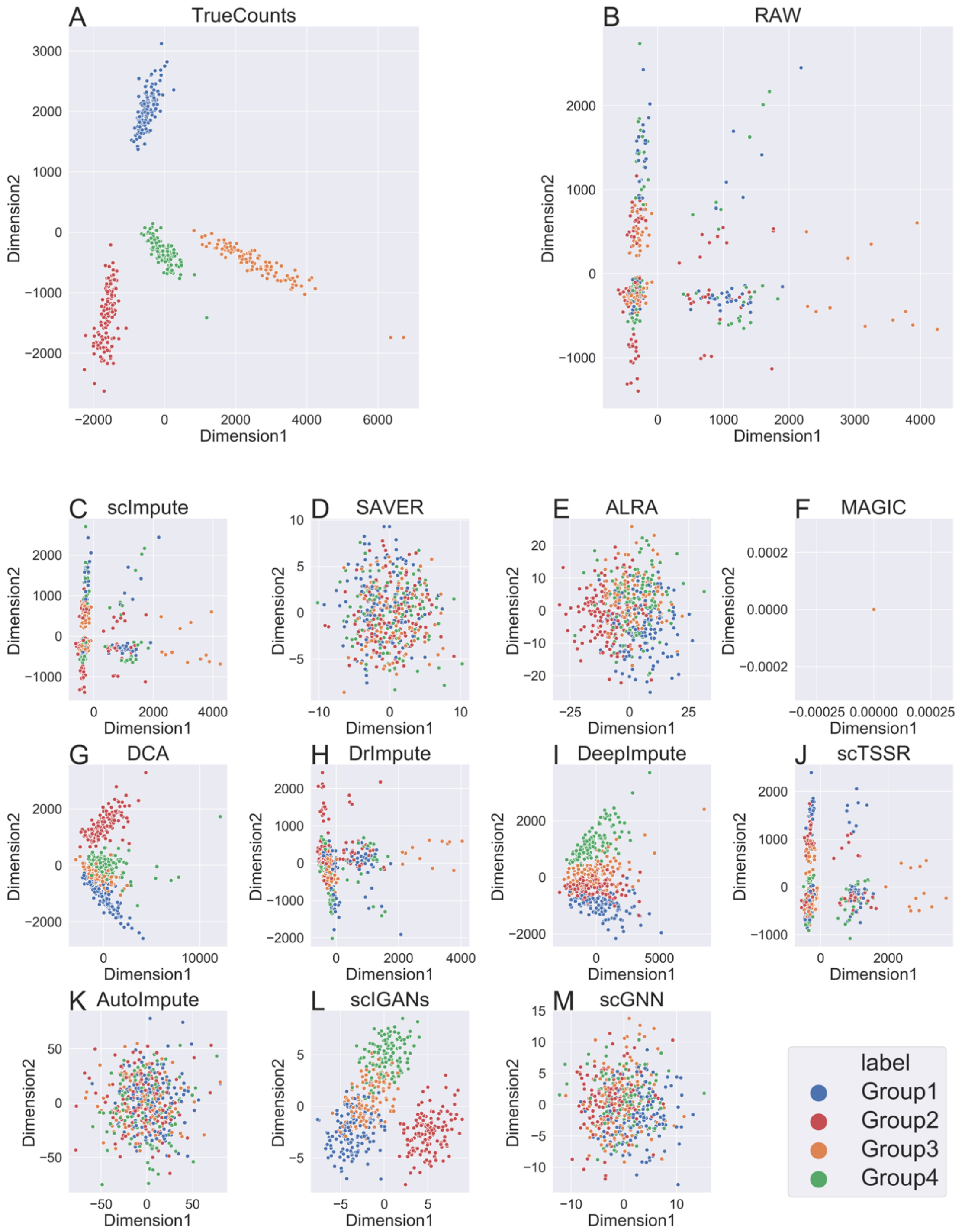

Figure S17. PCA plots of gene expression distribution of 11 methods on simulated dataset with zero expression rate of 0.48. (A-M) PCA plots on true counts matrix (without dropouts), raw gene expression matrix (with dropouts), and imputed matrices by scImpute, SAVER, ALRA, MAGIC, DCA, DrImpute, DeepImpute, scTSSR, AutoImpute, scIGANs, and scGNN, respectively.

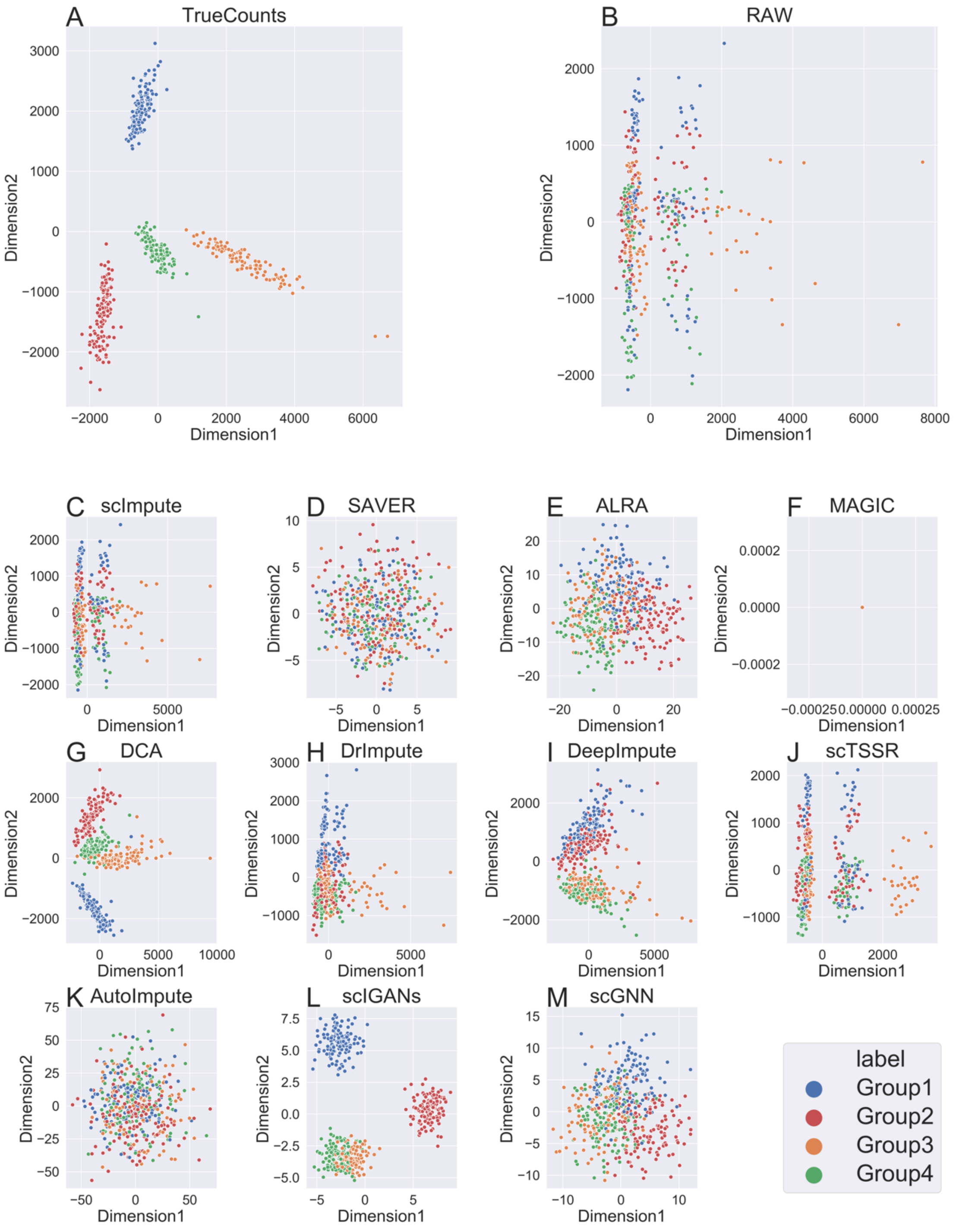

Figure S18. PCA plots of gene expression distribution of 11 methods on simulated dataset with zero expression rate of 0.42. (A-M) PCA plots on true counts matrix (without dropouts), raw gene expression matrix (with dropouts), and imputed matrices by scImpute, SAVER, ALRA, MAGIC, DCA, DrImpute, DeepImpute, scTSSR, AutoImpute, scIGANs, and scGNN, respectively.

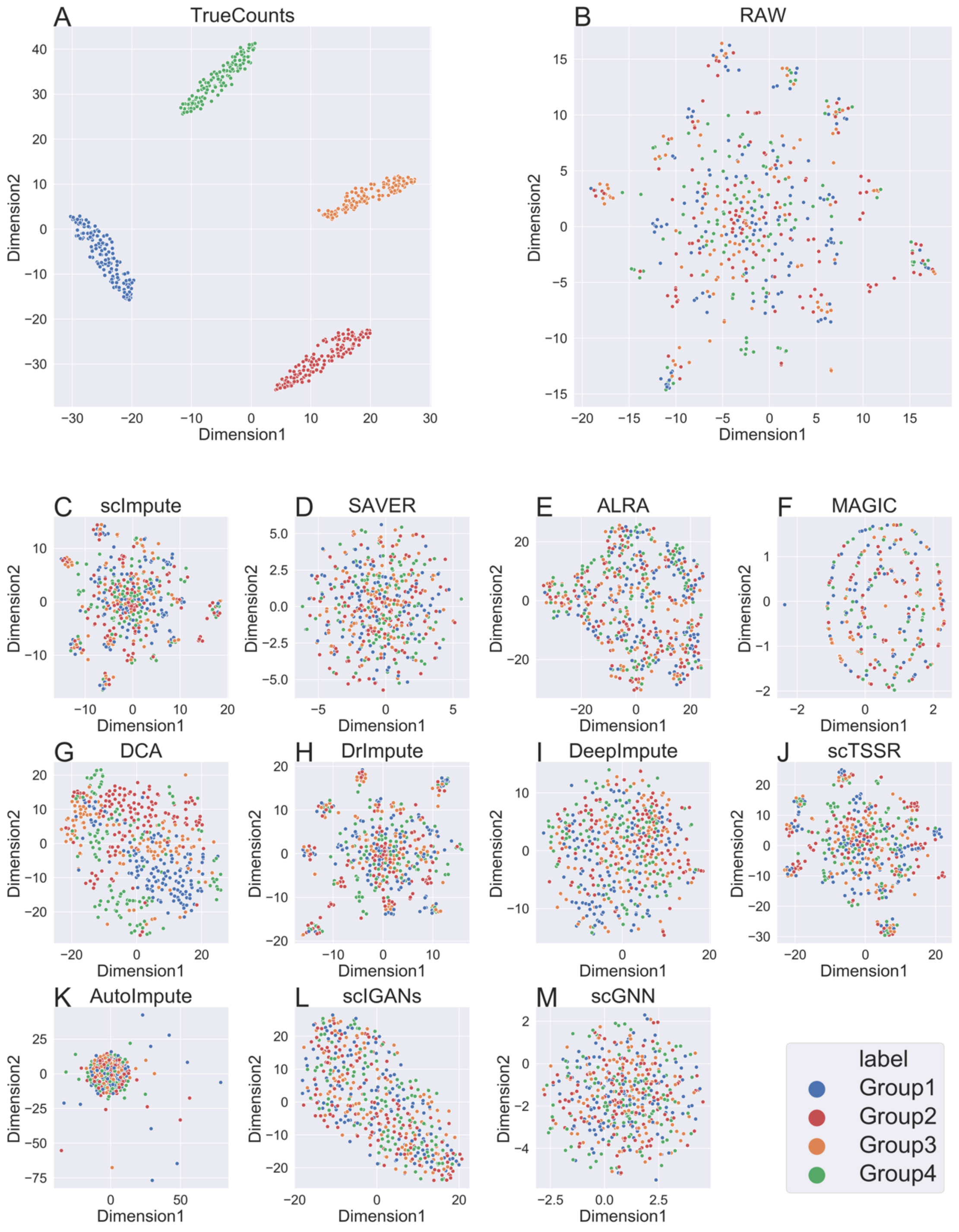

Figure S19. t-SNE plots of gene expression distribution of 11 methods on simulated dataset with zero expression rate of 0.78. (A-M) T-SNE plots on true counts matrix (without dropouts), raw gene expression matrix (with dropouts), and imputed matrices by scImpute, SAVER, ALRA, MAGIC, DCA, DrImpute, DeepImpute, scTSSR, AutoImpute, scIGANs, and scGNN, respectively.

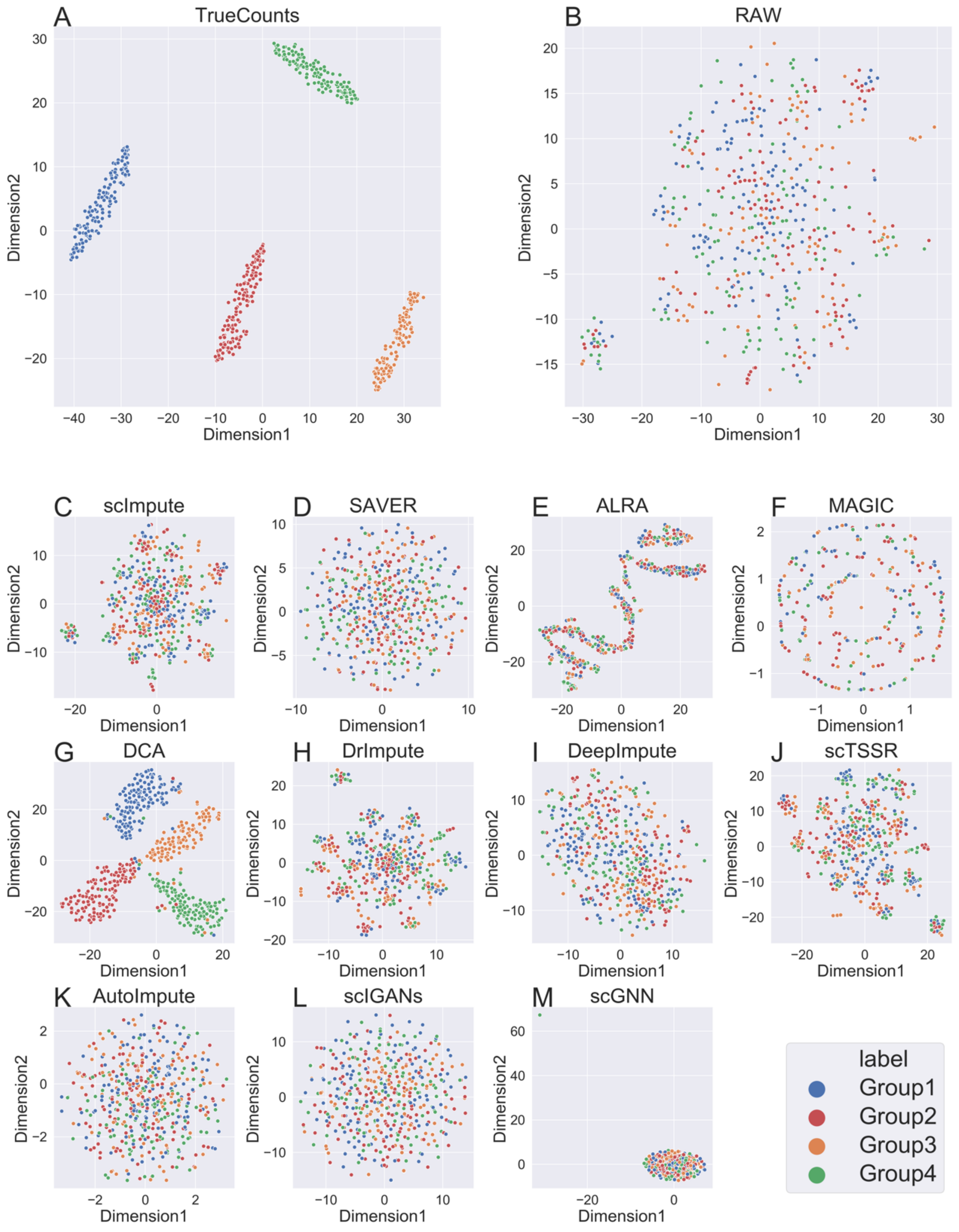

Figure S20. t-SNE plots of gene expression distribution of 11 methods on simulated dataset with zero expression rate of 0.71. (A-M) T-SNE plots on true counts matrix (without dropouts), raw gene expression matrix (with dropouts), and imputed matrices by scImpute, SAVER, ALRA, MAGIC, DCA, DrImpute, DeepImpute, scTSSR, AutoImpute, scIGANs, and scGNN, respectively.

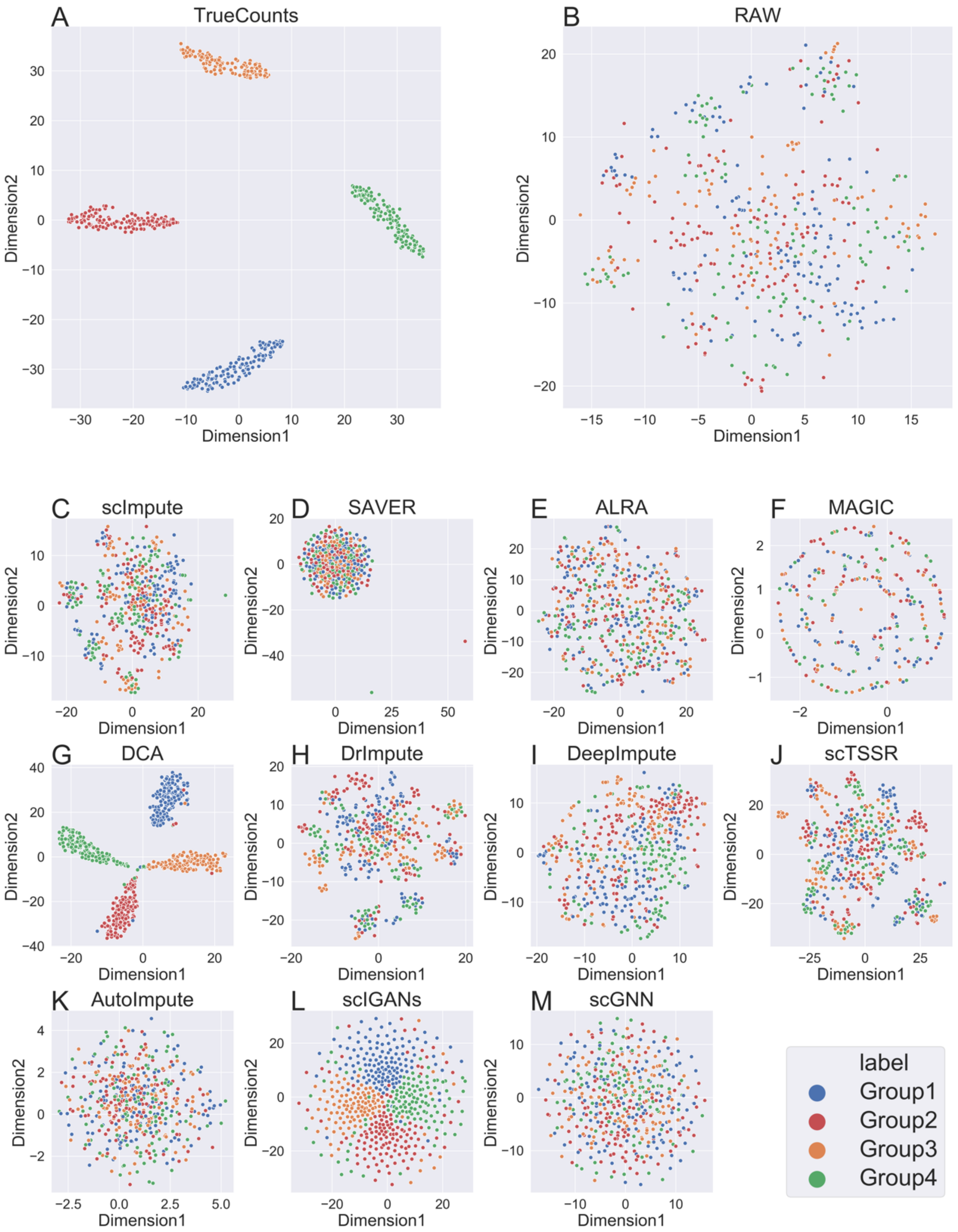

Figure S21. t-SNE plots of gene expression distribution of 11 methods on simulated dataset with zero expression rate of 0.63. (A-M) T-SNE plots on true counts matrix (without dropouts), raw gene expression matrix (with dropouts), and imputed matrices by scImpute, SAVER, ALRA, MAGIC, DCA, DrImpute, DeepImpute, scTSSR, AutoImpute, scIGANs, and scGNN, respectively.

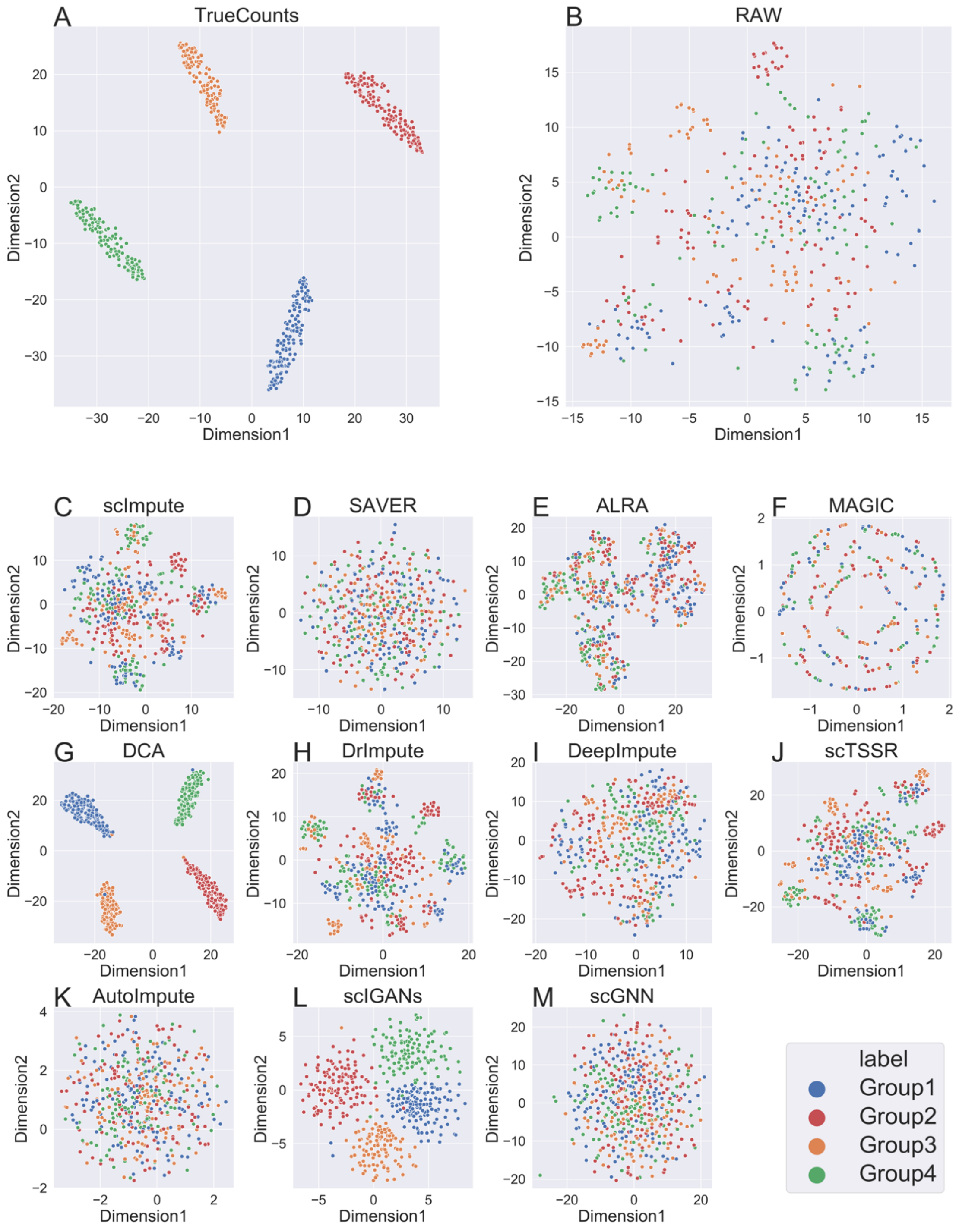

Figure S22. t-SNE plots of gene expression distribution of 11 methods on simulated dataset with zero expression rate of 0.55. (A-M) T-SNE plots on true counts matrix (without dropouts), raw gene expression matrix (with dropouts), and imputed matrices by scImpute, SAVER, ALRA, MAGIC, DCA, DrImpute, DeepImpute, scTSSR, AutoImpute, scIGANs, and scGNN, respectively.

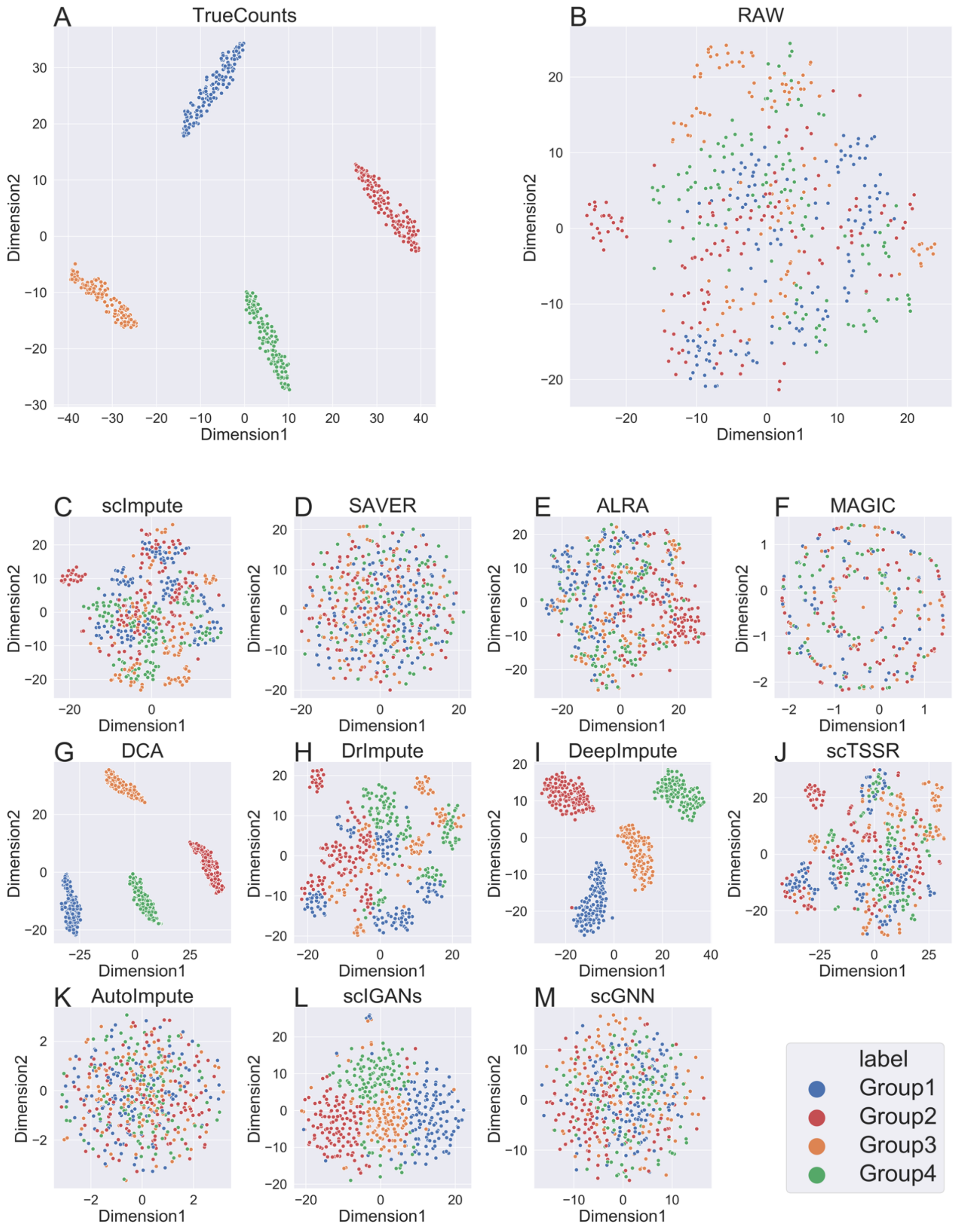

Figure S23. t-SNE plots of gene expression distribution of 11 methods on simulated dataset with zero expression rate of 0.48. (A-M) T-SNE plots on true counts matrix (without dropouts), raw gene expression matrix (with dropouts), and imputed matrices by scImpute, SAVER, ALRA, MAGIC, DCA, DrImpute, DeepImpute, scTSSR, AutoImpute, scIGANs, and scGNN, respectively.

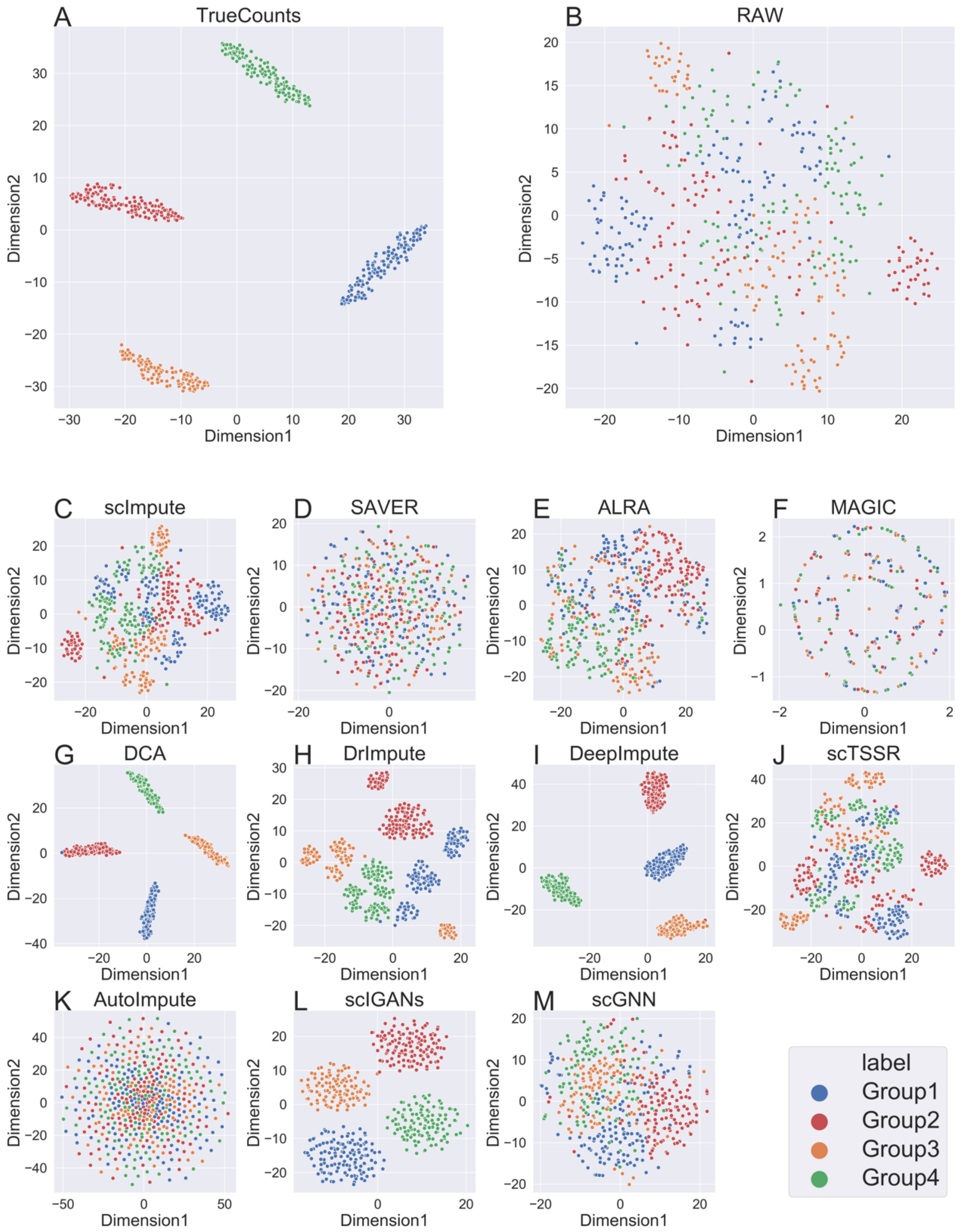

Figure S24. t-SNE plots of gene expression distribution of 11 methods on simulated dataset with zero expression rate of 0.42. (A-M) T-SNE plots on true counts matrix (without dropouts), raw gene expression matrix (with dropouts), and imputed matrices by scImpute, SAVER, ALRA, MAGIC, DCA, DrImpute, DeepImpute, scTSSR, AutoImpute, scIGANs, and scGNN, respectively.

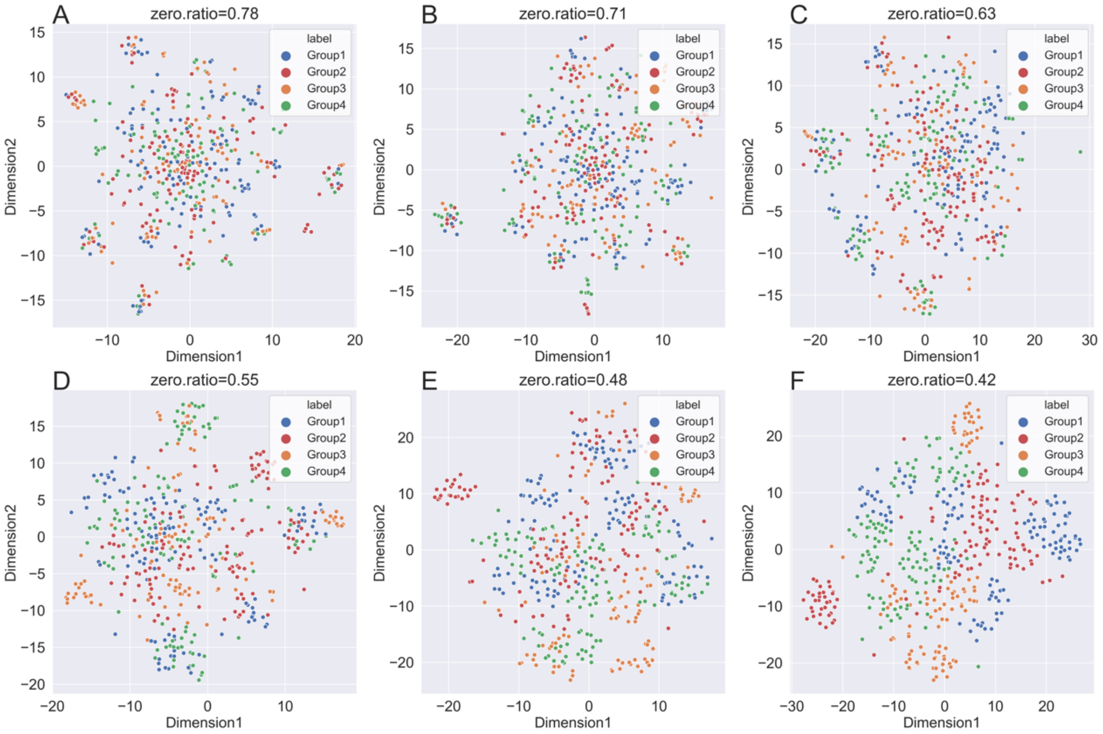

Figure S25. t-SNE plots of imputed data for scImpute in six simulated datasets with different zero expression rates. (A-F) T-SNE plots on simulated dataset with zero expression rate of 0.78, 0.71, 0.63, 0.55, 0.48, and 0.42.

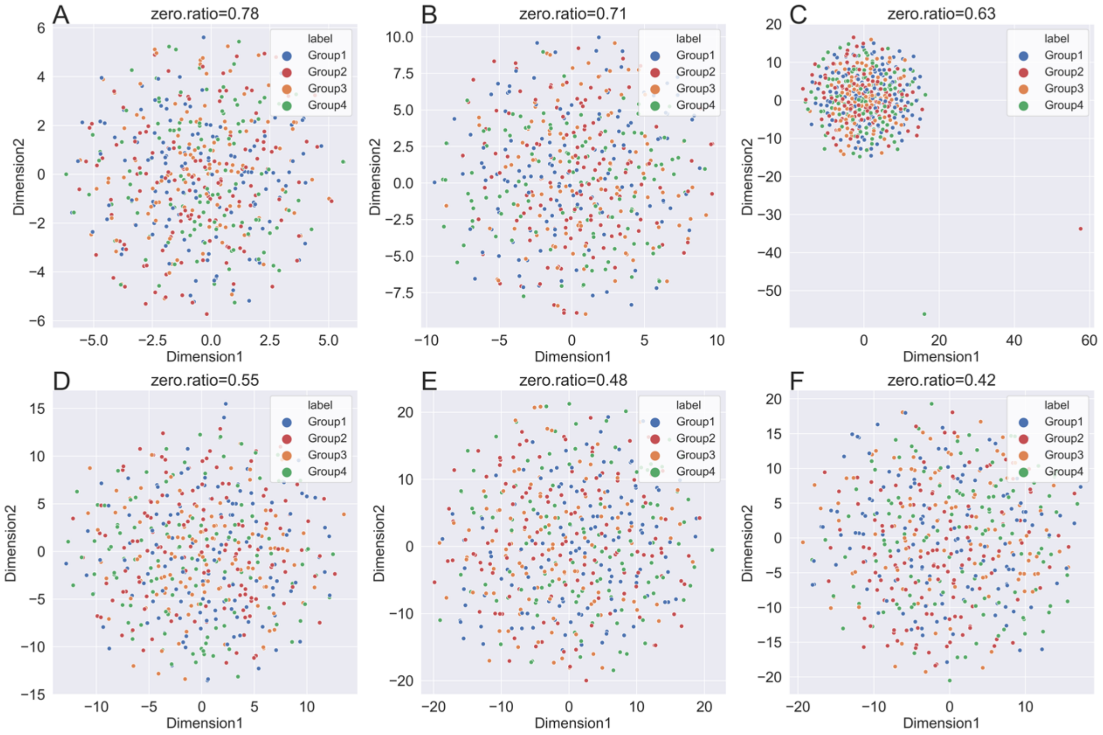

Figure S26. t-SNE plots of imputed data for SAVER in six simulated datasets with different zero expression rates. (A-F) T-SNE plots on simulated dataset with zero expression rate of 0.78, 0.71, 0.63, 0.55, 0.48, and 0.42.

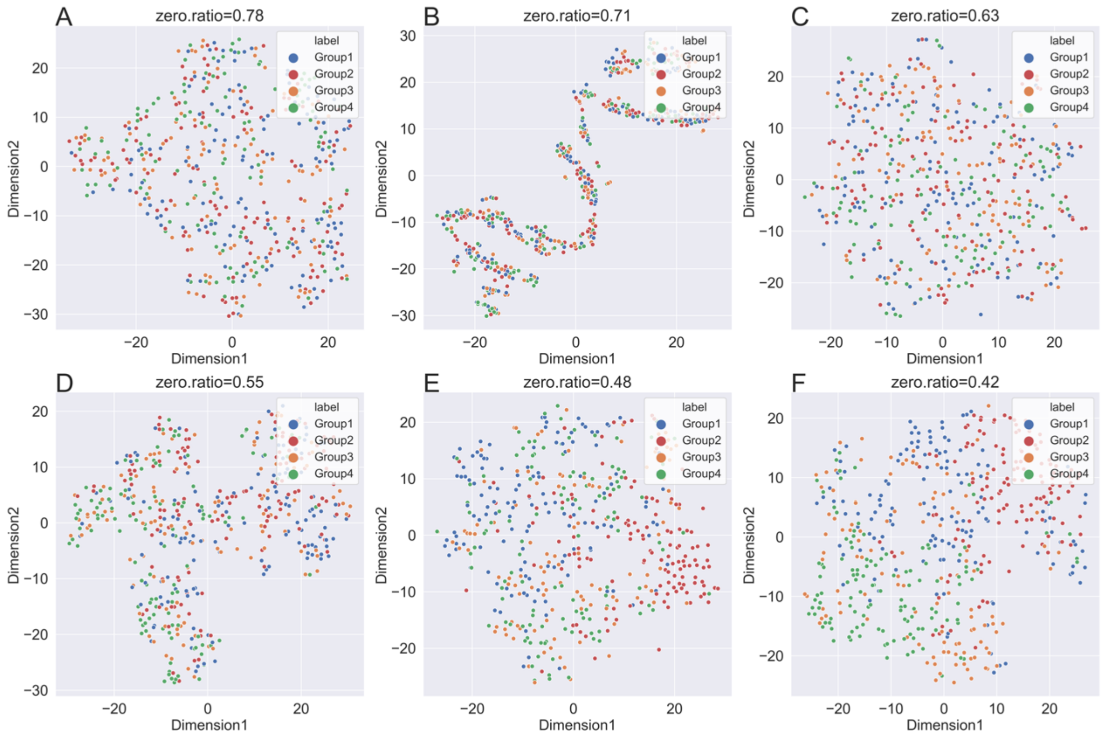

Figure S27. t-SNE plots of imputed data for ALRA in six simulated datasets with different zero expression rates. (A-F) T-SNE plots on simulated dataset with zero expression rate of 0.78, 0.71, 0.63, 0.55, 0.48, and 0.42.

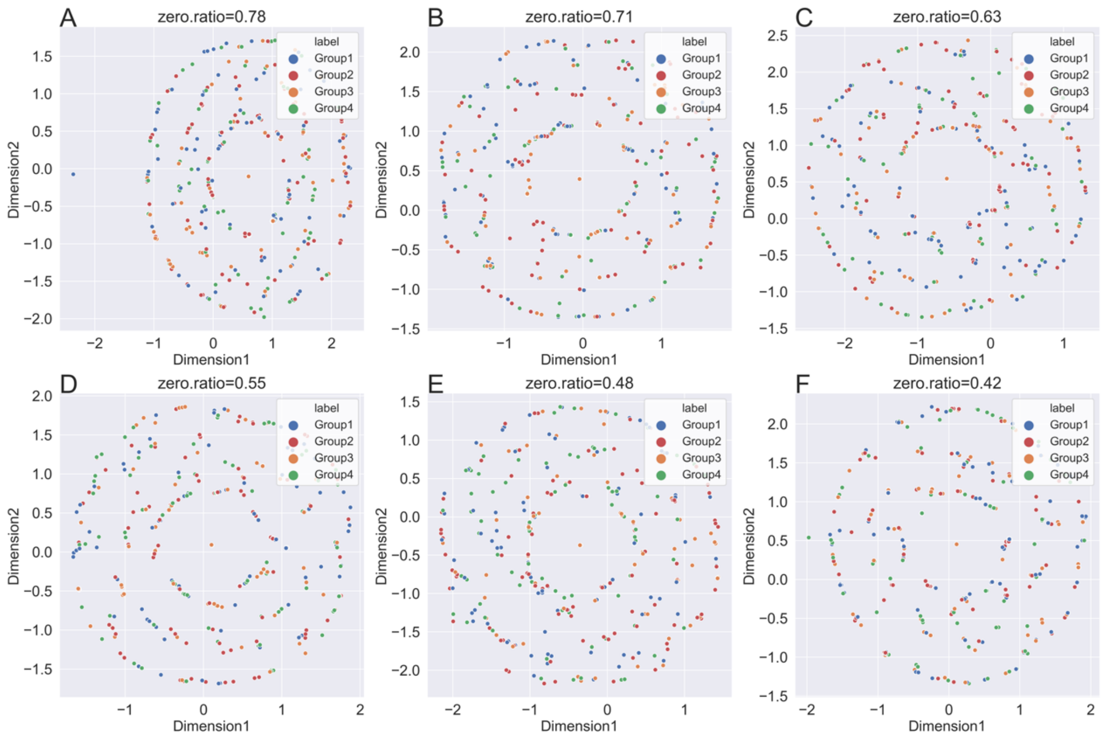

Figure S28. t-SNE plots of imputed data for MAGIC in six simulated datasets with different zero expression rates. (A-F) T-SNE plots on simulated dataset with zero expression rate of 0.78, 0.71, 0.63, 0.55, 0.48, and 0.42.

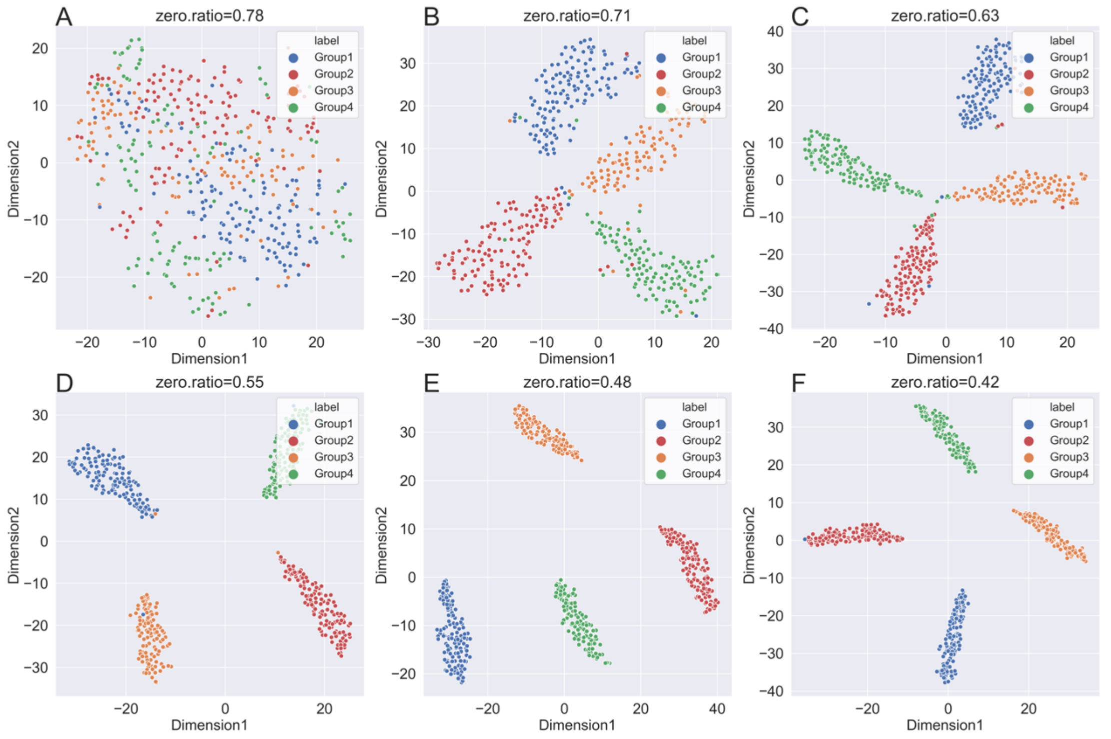

Figure S29. t-SNE plots of imputed data for DCA in six simulated datasets with different zero expression rates. (A-F) T-SNE plots on simulated dataset with zero expression rate of 0.78, 0.71, 0.63, 0.55, 0.48, and 0.42.

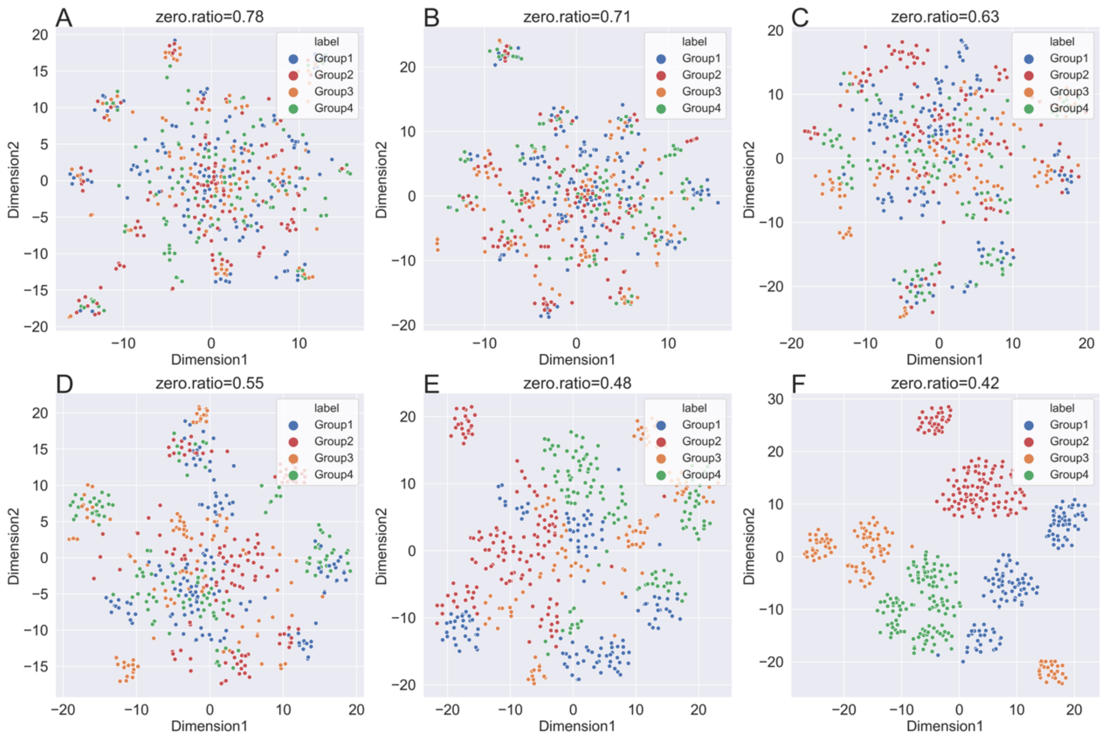

Figure S30. t-SNE plots of imputed data for DrImpute in six simulated datasets with different zero expression rates. (A-F) T-SNE plots on simulated dataset with zero expression rate of 0.78, 0.71, 0.63, 0.55, 0.48, and 0.42.

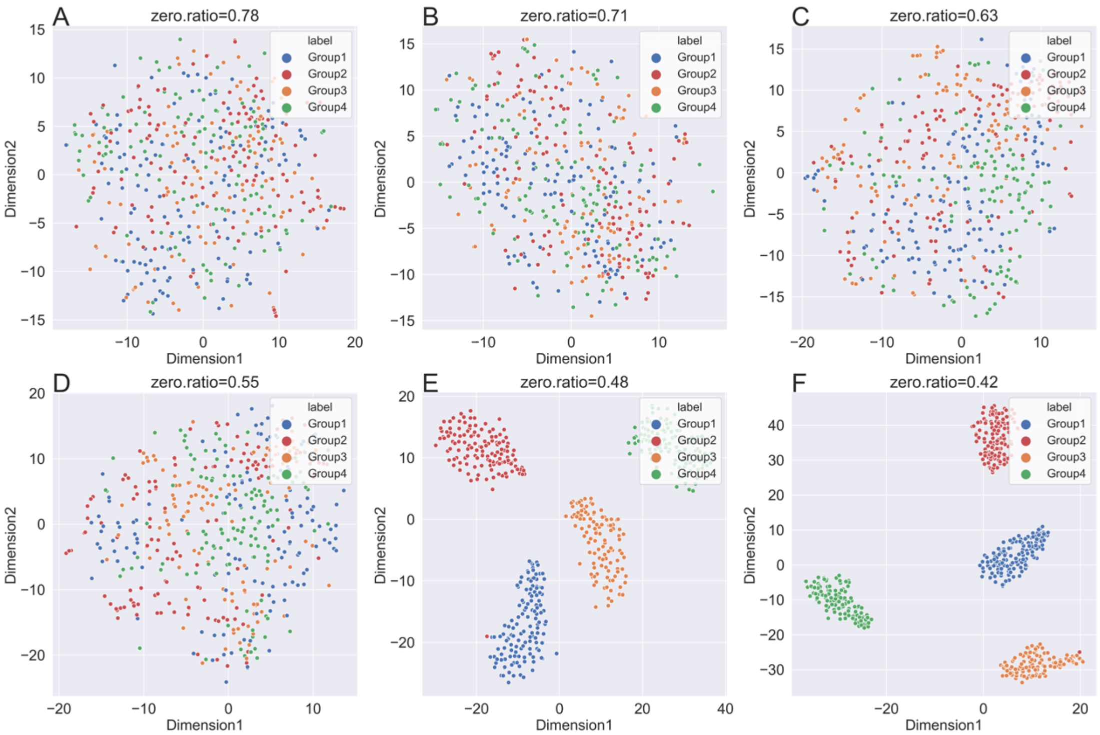

Figure S31. t-SNE plots of imputed data for DeepImpute in six simulated datasets with different zero expression rates. (A-F) T-SNE plots on simulated dataset with zero expression rate of 0.78, 0.71, 0.63, 0.55, 0.48, and 0.42.

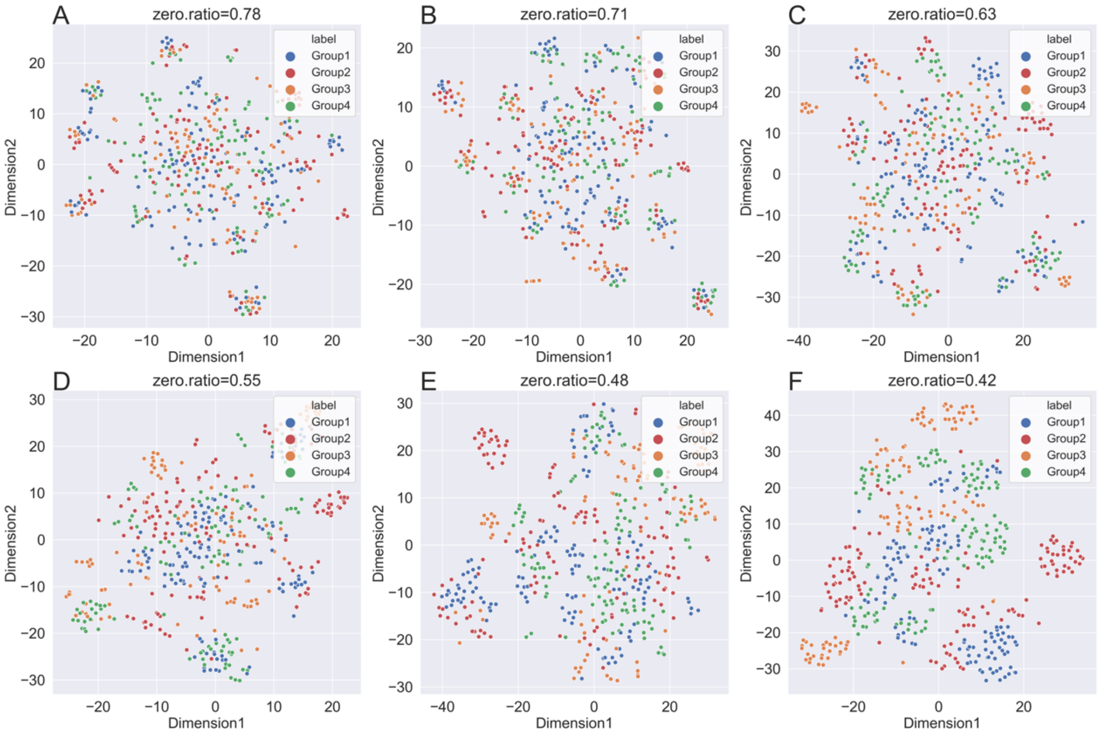

Figure S32. t-SNE plots of imputed data for scTSSR in six simulated datasets with different zero expression rates. (A-F) T-SNE plots on simulated dataset with zero expression rate of 0.78, 0.71, 0.63, 0.55, 0.48, and 0.42.

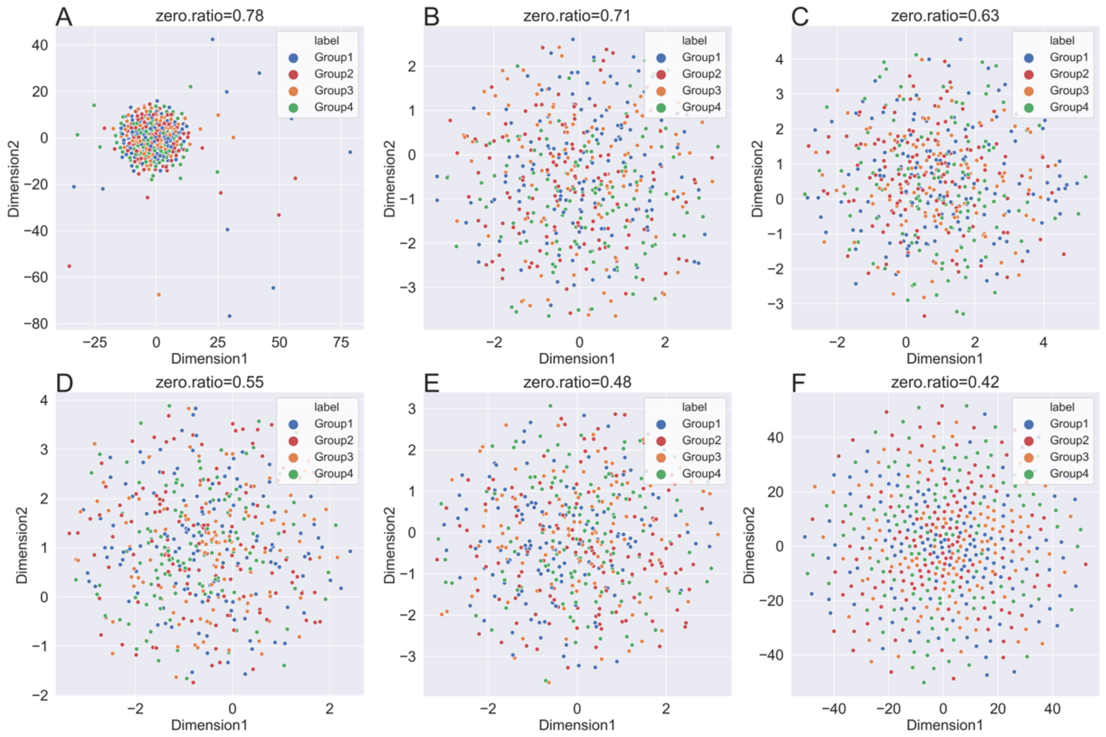

Figure S33. t-SNE plots of imputed data for AutoImpute in six simulated datasets with different zero expression rates. (A-F) T-SNE plots on simulated dataset with zero expression rate of 0.78, 0.71, 0.63, 0.55, 0.48, and 0.42.

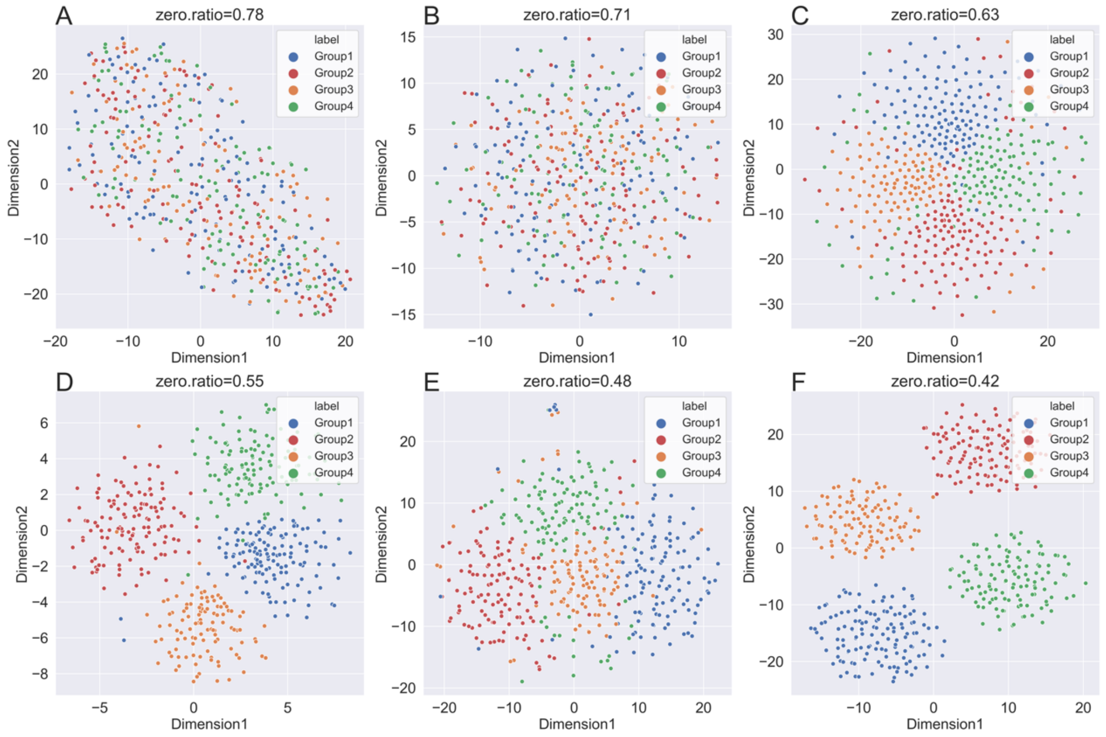

Figure S34. t-SNE plots of imputed data for scIGANs in six simulated datasets with different zero expression rates. (A-F) T-SNE plots on simulated dataset with zero expression rate of 0.78, 0.71, 0.63, 0.55, 0.48, and 0.42.

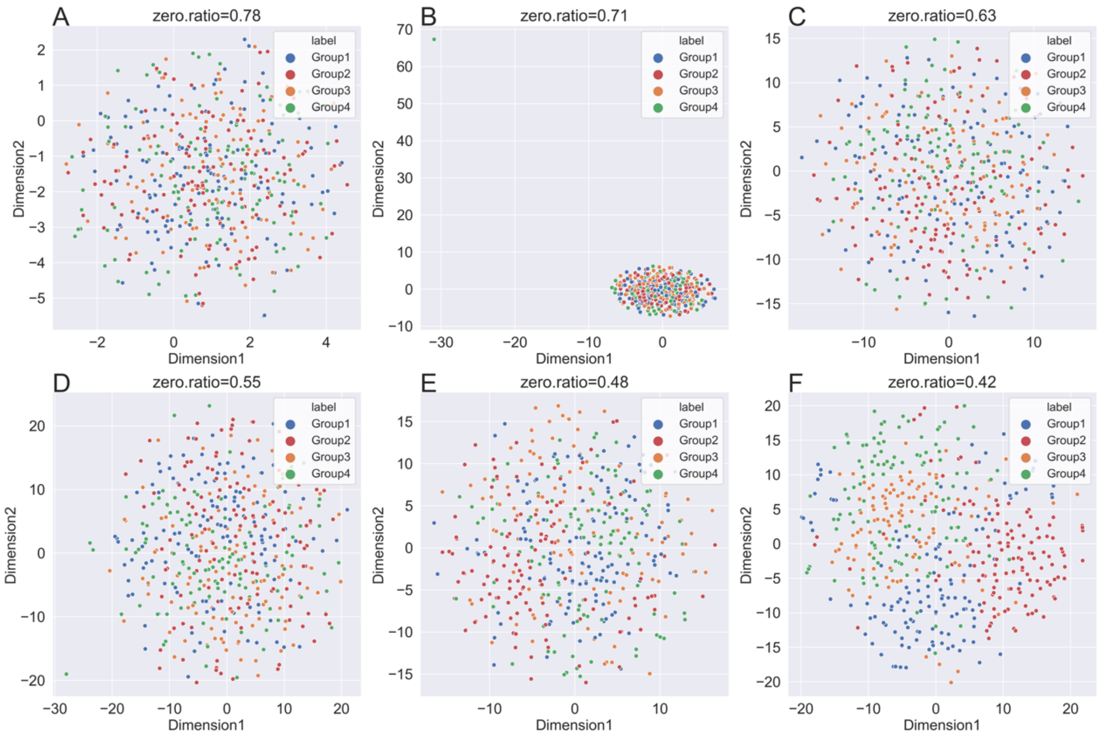

Figure S35. t-SNE plots of imputed data for scGNN in six simulated datasets with different zero expression rates. (A-F) T-SNE plots on simulated dataset with zero expression rate of 0.78, 0.71, 0.63, 0.55, 0.48, and 0.42.

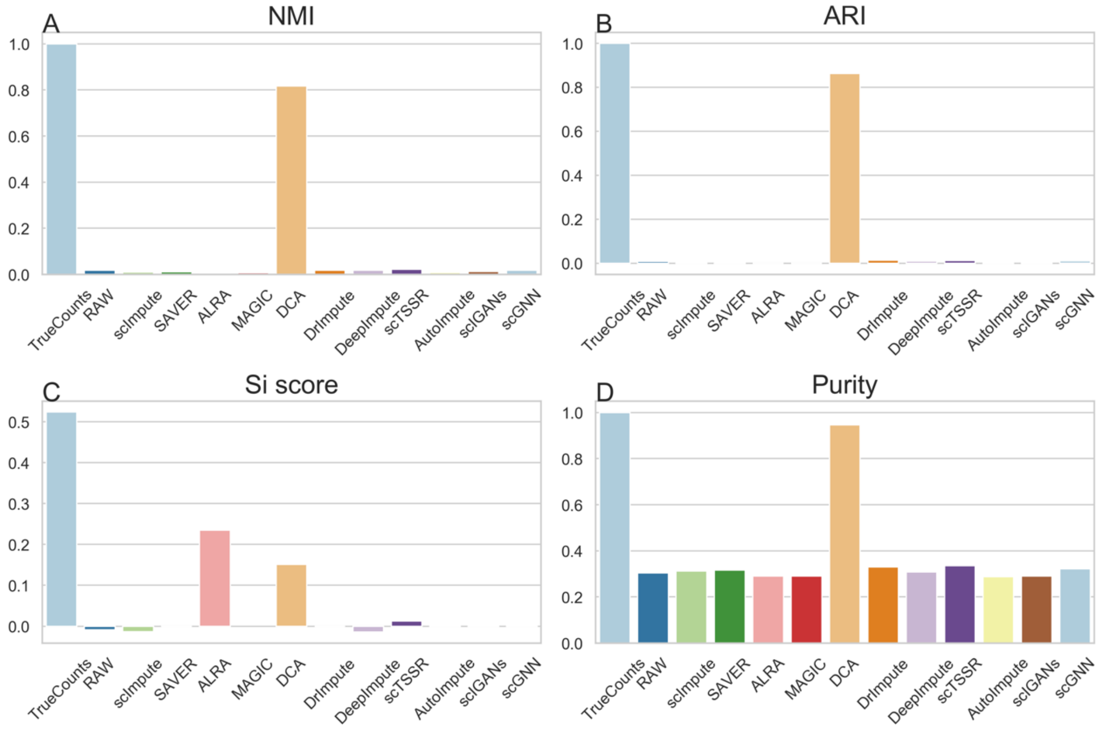

Figure S36. Performance evaluation of 11 imputation methods for cell clustering at dataset with zero expression rate of 0.71. (A-D) NMI, ARI, Si, and Purity scores of the clustering results of 11 imputation methods obtained by t-SNE

and k-means on simulated dataset.

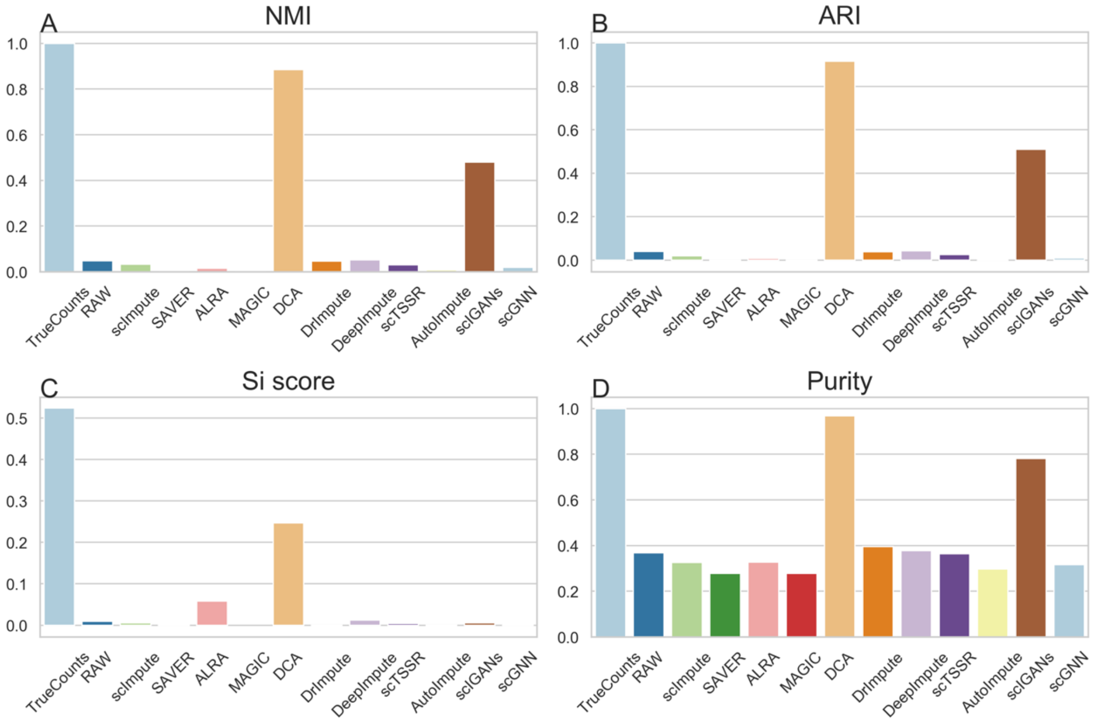

Figure S37. Performance evaluation of 11 imputation methods for cell clustering at dataset with zero expression rate of 0.63. (A-D) NMI, ARI, Si, and Purity scores of the clustering results of 11 imputation methods obtained by t-SNE and k-means on simulated dataset.

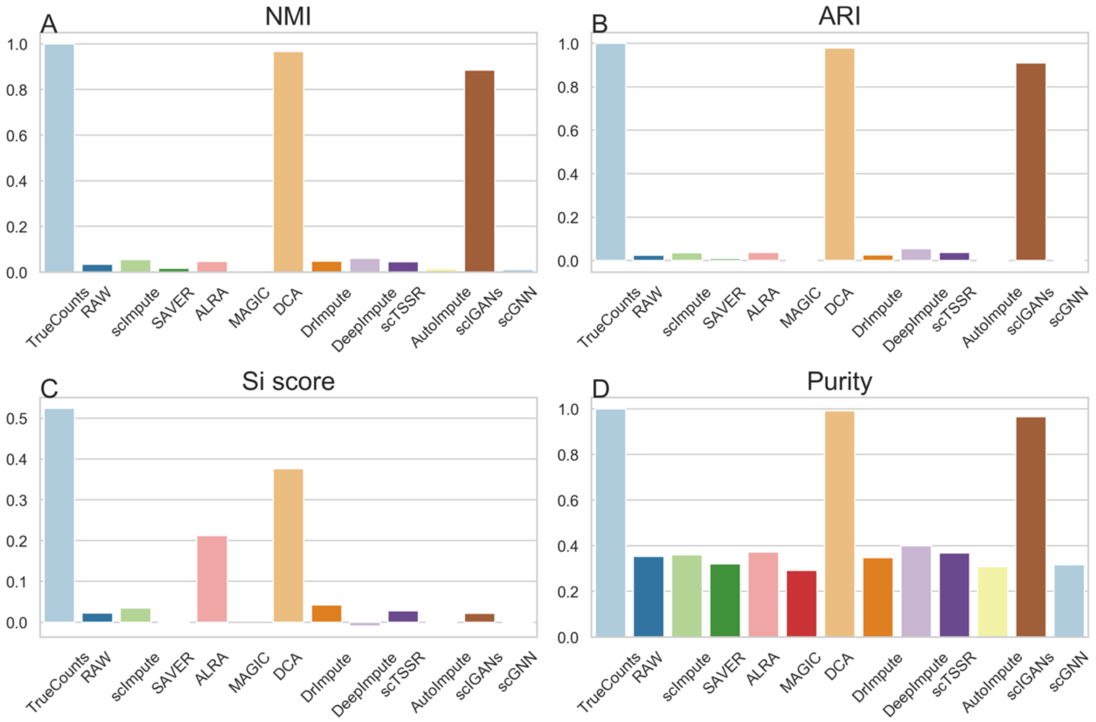

Figure S38. Performance evaluation of 11 imputation methods for cell

clustering at dataset with zero expression rate of 0.55. (A-D) NMI, ARI, Si, and Purity scores of the clustering results of 11 imputation methods obtained by t-SNE and k-means on simulated dataset.

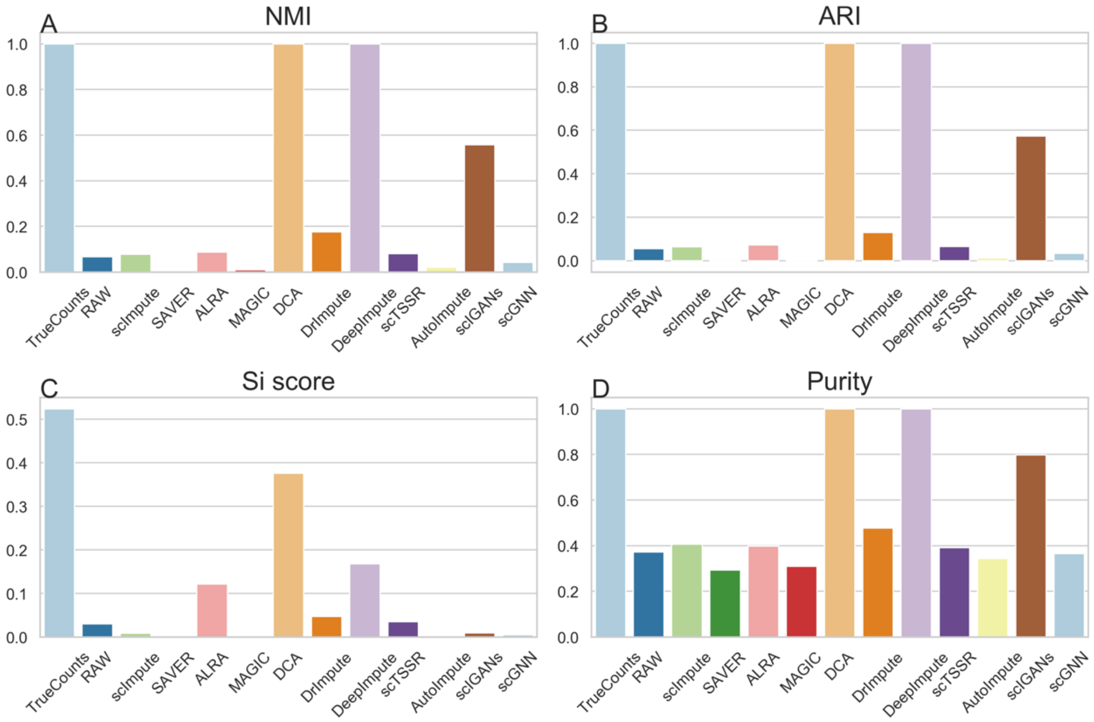

Figure S39. Performance evaluation of 11 imputation methods for cell clustering at dataset with zero expression rate of 0.48. (A-D) NMI, ARI, Si, and Purity scores of the clustering results of 11 imputation methods obtained by t-SNE and k-means on simulated dataset.

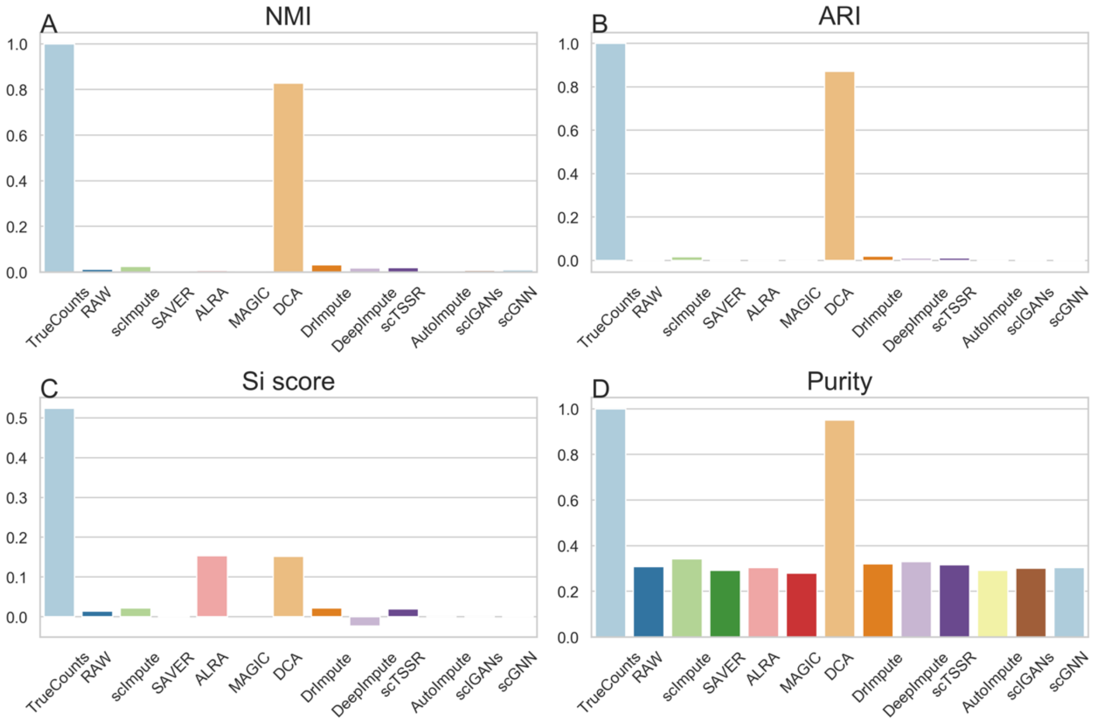

Figure S40. Performance evaluation of 11 imputation methods for cell clustering at dataset with zero expression rate of 0.71. (A-D) NMI, ARI, Si, and Purity scores of the clustering results of 11 imputation methods obtained by UMAP and k-means on simulated dataset.

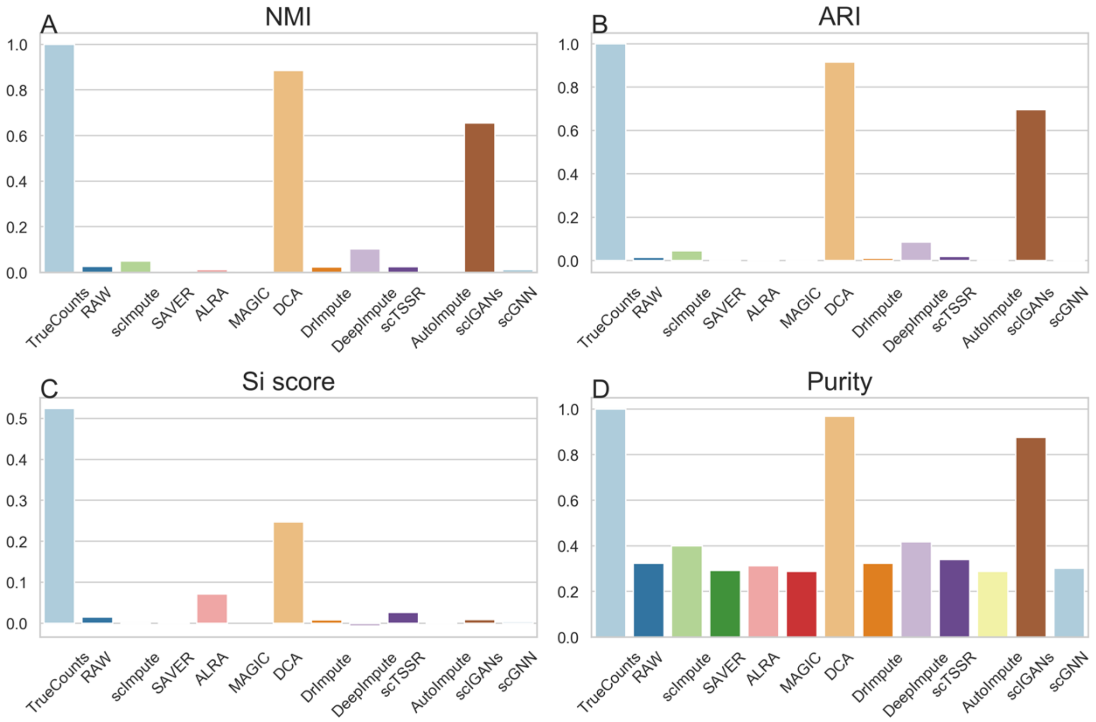

Figure S41. Performance evaluation of 11 imputation methods for cell clustering at dataset with zero expression rate of 0.63. (A-D) NMI, ARI, Si, and Purity scores of the clustering results of 11 imputation methods obtained by UMAP

and k-means on simulated dataset.

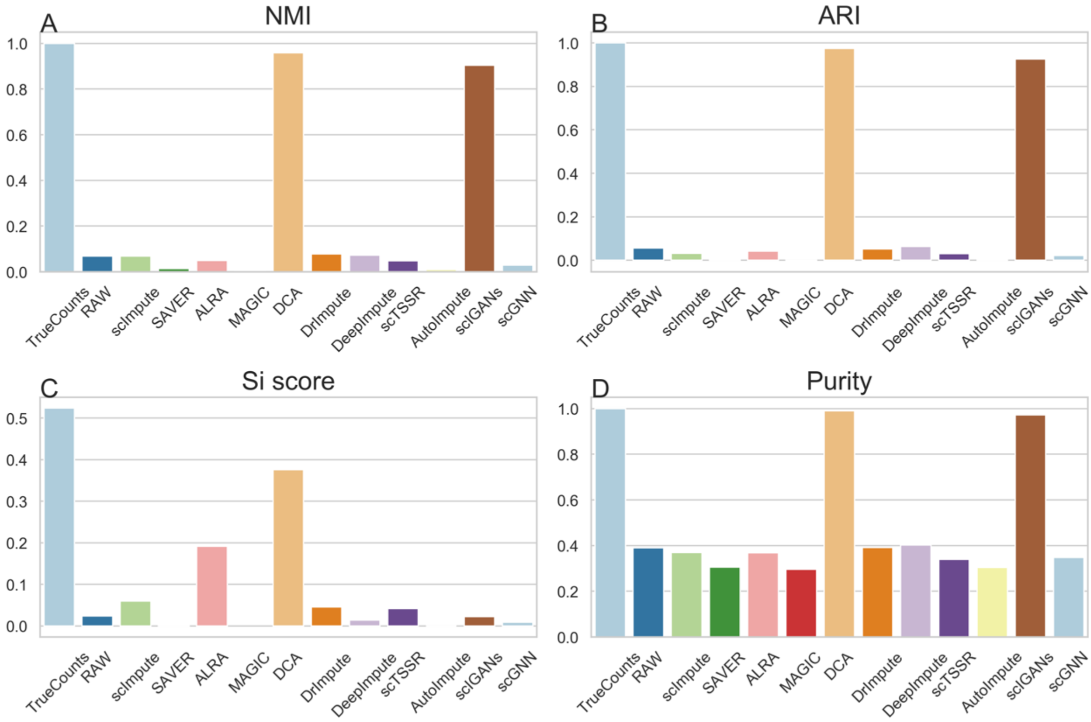

Figure S42. Performance evaluation of 11 imputation methods for cell clustering at dataset with zero expression rate of 0.55. (A-D) NMI, ARI, Si, and Purity scores of the clustering results of 11 imputation methods obtained by UMAP and k-means on simulated dataset.

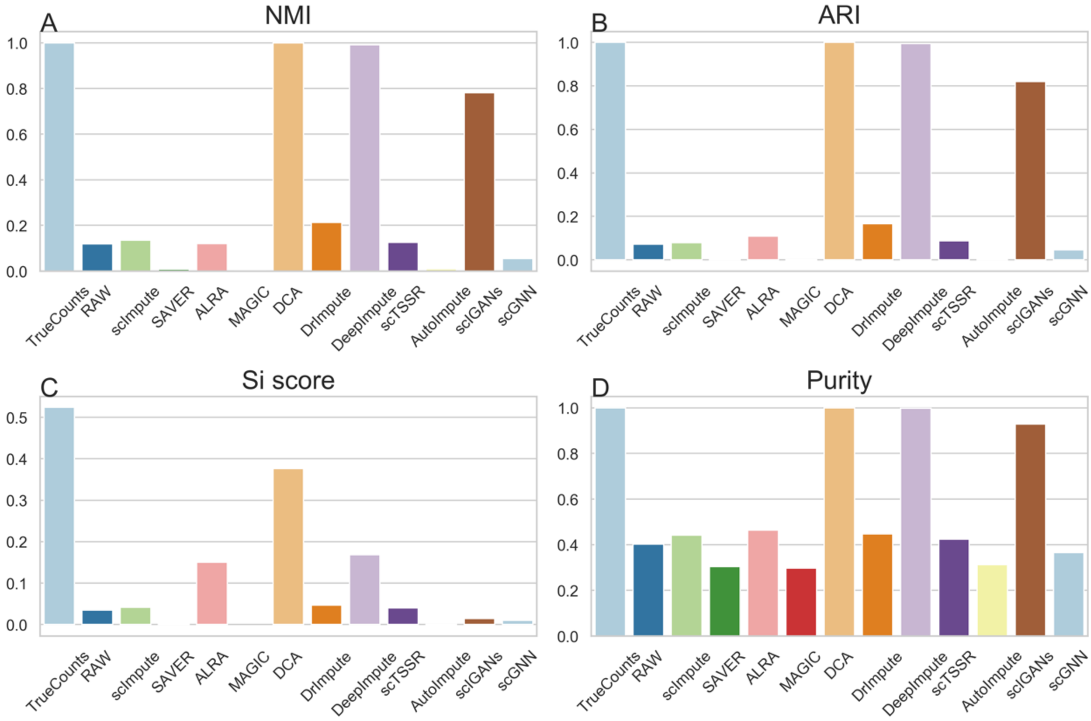

Figure S43. Performance evaluation of 11 imputation methods for cell

clustering at dataset with zero expression rate of 0.48. (A-D) NMI, ARI, Si, and Purity scores of the clustering results of 11 imputation methods obtained by UMAP and k-means on simulated dataset.

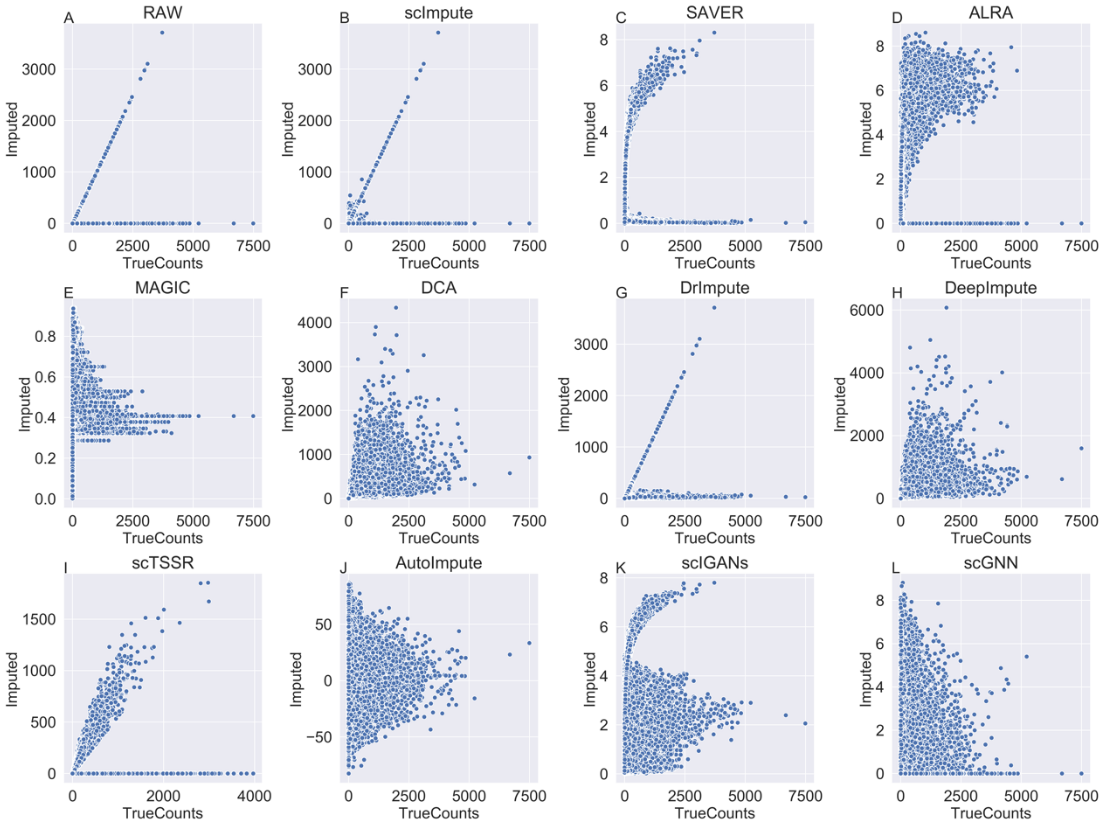

Figure S44. Scatter plots for the true gene expression values and imputed gene expression values for dataset with zero expression rate of 0.78. (A-L) Results from RAW data, and imputed data by scImpute, SAVER, ALRA, MAGIC, DCA, DrImpute, DeepImpute, scTSSR, AutoImpute, scIGANs, and scGNN. The *x-axis* represents true gene expression value, and the *y-axis* represents imputed gene expression value.

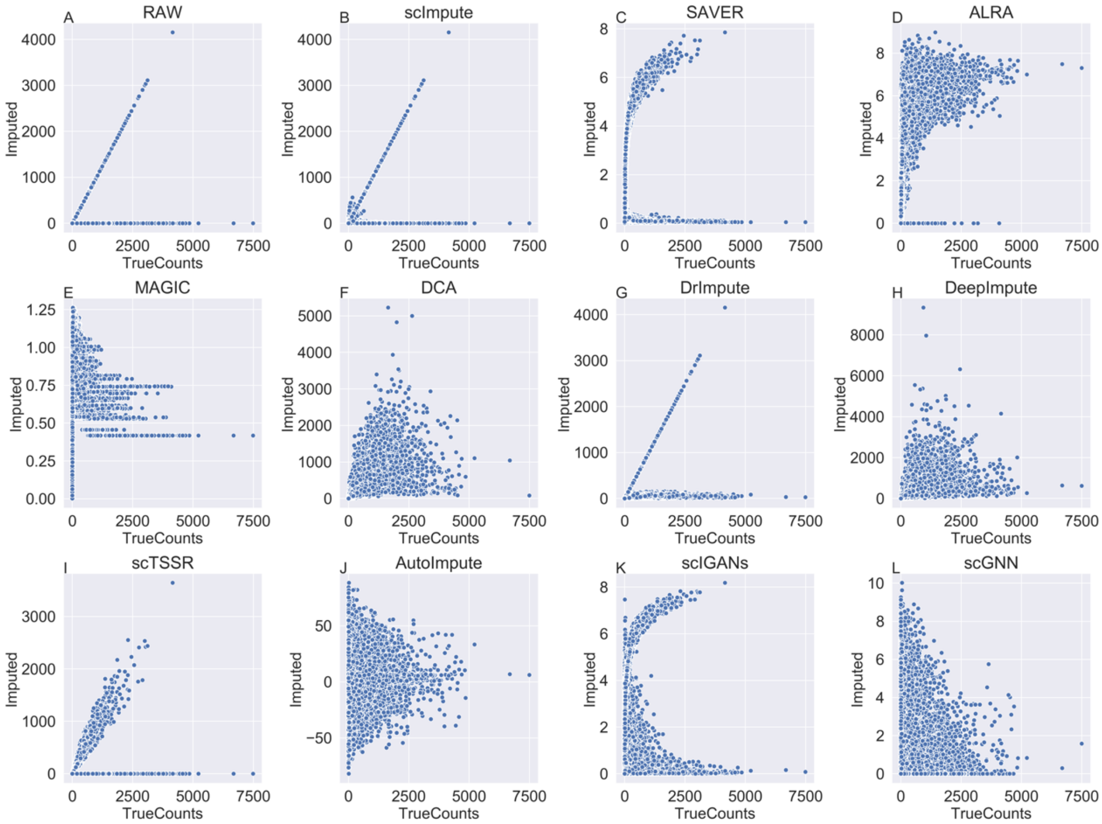

Figure S45. Scatter plots for the true gene expression values and imputed gene expression values for dataset with zero expression rate of 0.71. (A-L) Results from RAW data, and imputed data by scImpute, SAVER, ALRA, MAGIC, DCA, DrImpute, DeepImpute, scTSSR, AutoImpute, scIGANs, and scGNN. The *x-axis* represents true gene expression value, and the *y-axis* represents imputed gene expression value.

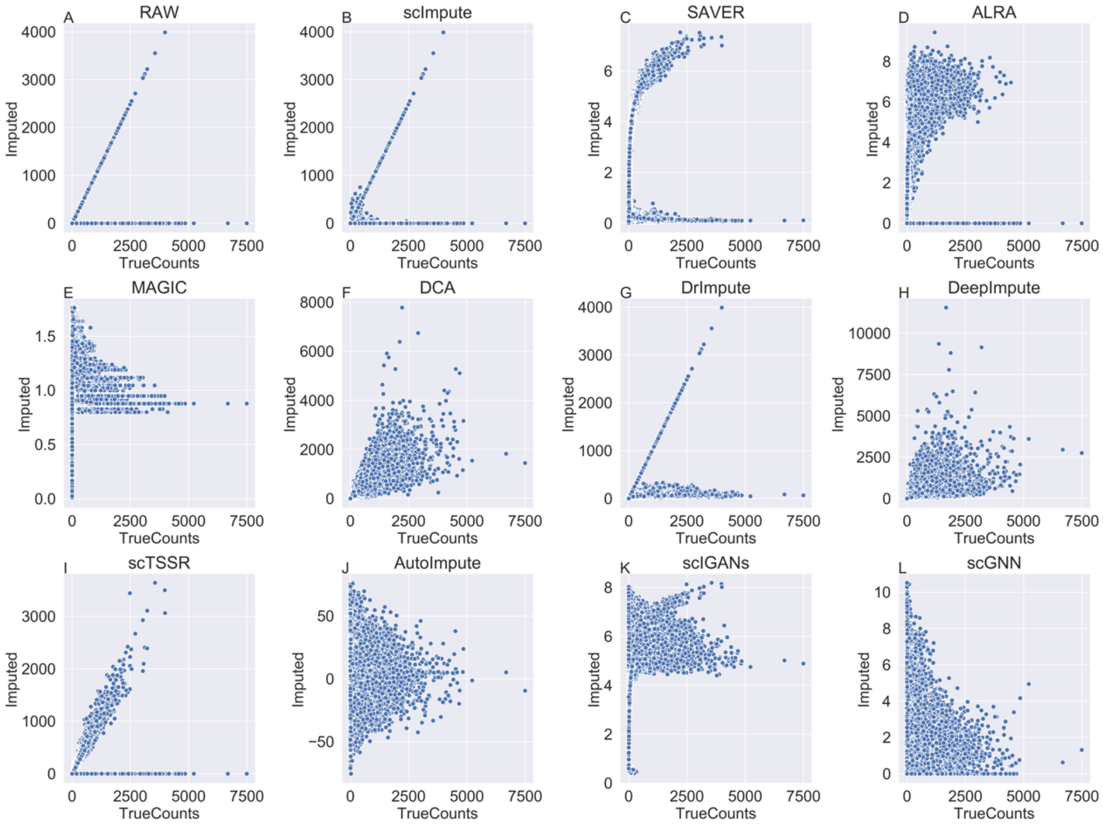

Figure S46. Scatter plots for the true gene expression values and imputed gene expression values for dataset with zero expression rate of 0.63. (A-L) Results from RAW data, and imputed data by scImpute, SAVER, ALRA, MAGIC, DCA, DrImpute, DeepImpute, scTSSR, AutoImpute, scIGANs, and scGNN. The *x-axis* represents true gene expression value, and the *y-axis* represents imputed gene expression value.

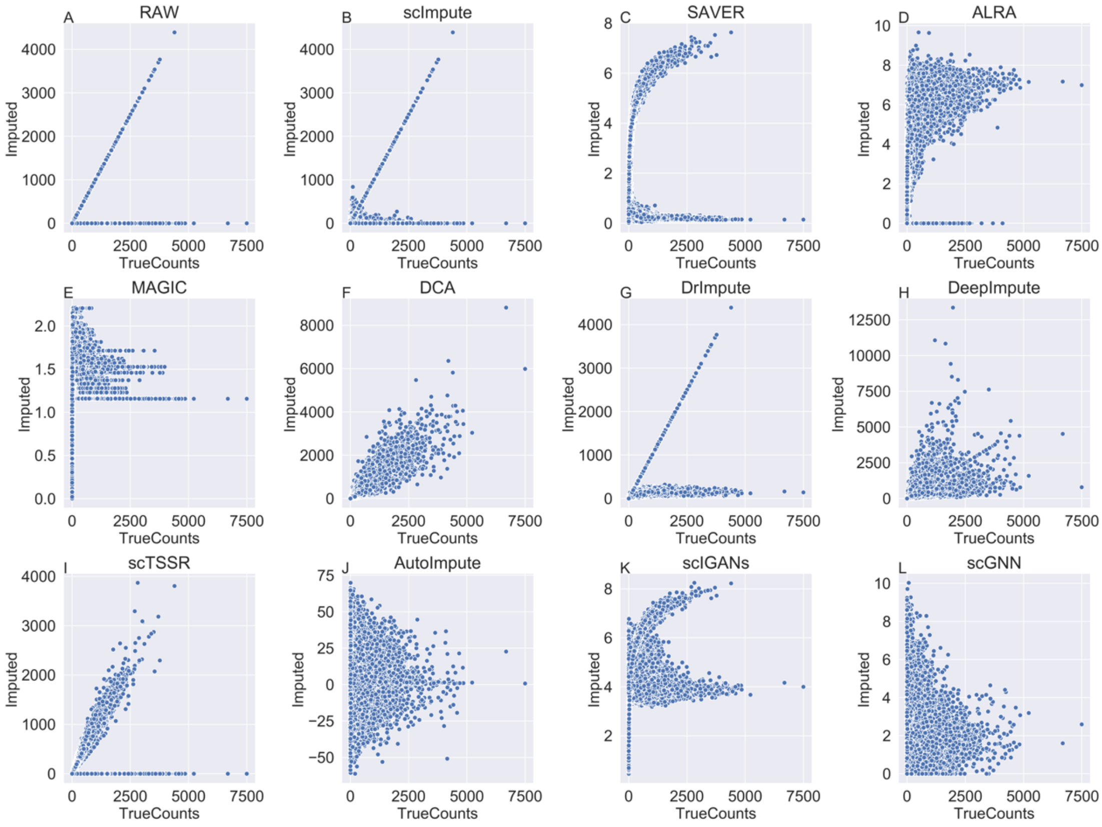

Figure S47. Scatter plots for the true gene expression values and imputed gene expression values for dataset with zero expression rate of 0.55. (A-L) Results from RAW data, and imputed data by scImpute, SAVER, ALRA, MAGIC, DCA, DrImpute, DeepImpute, scTSSR, AutoImpute, scIGANs, and scGNN. The *x-axis* represents true gene expression value, and the *y-axis* represents imputed gene expression value.

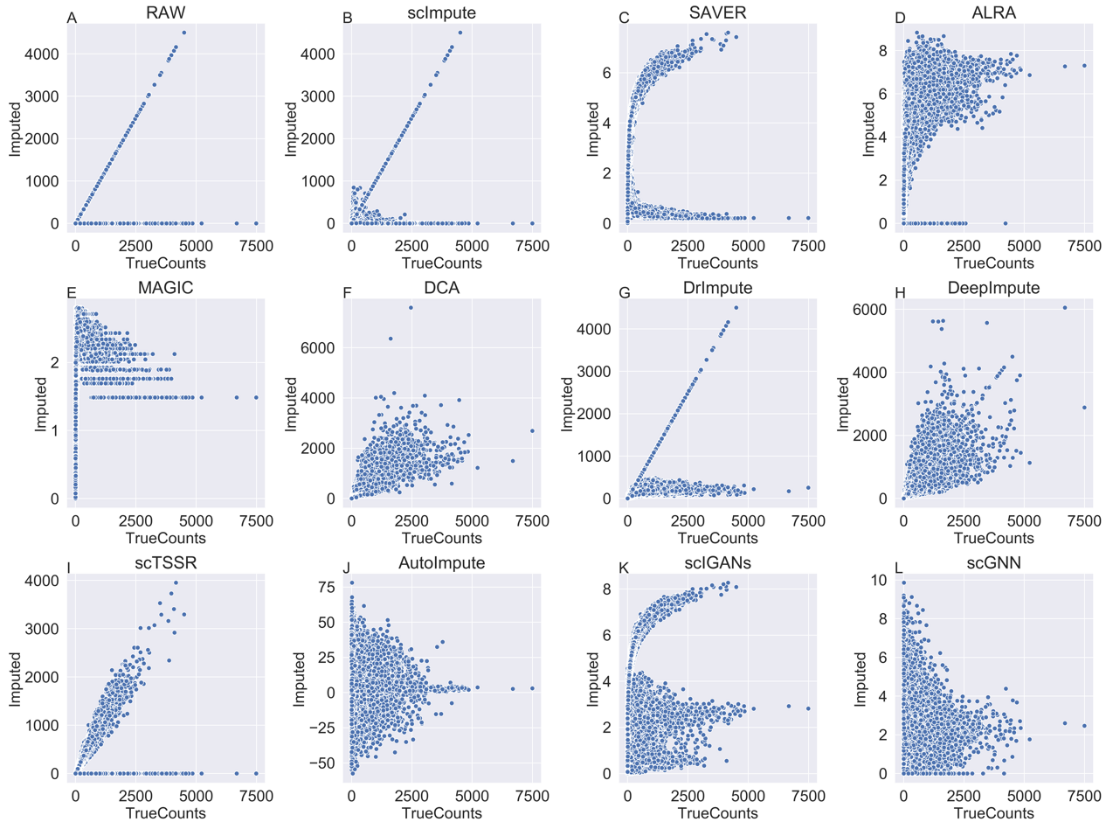

Figure S48. Scatter plots for the true gene expression values and imputed gene expression values for dataset with zero expression rate of 0.48. (A-L) Results from RAW data, and imputed data by scImpute, SAVER, ALRA, MAGIC, DCA, DrImpute, DeepImpute, scTSSR, AutoImpute, scIGANs, and scGNN. The *x-axis* represents true gene expression value, and the *y-axis* represents imputed gene expression value.

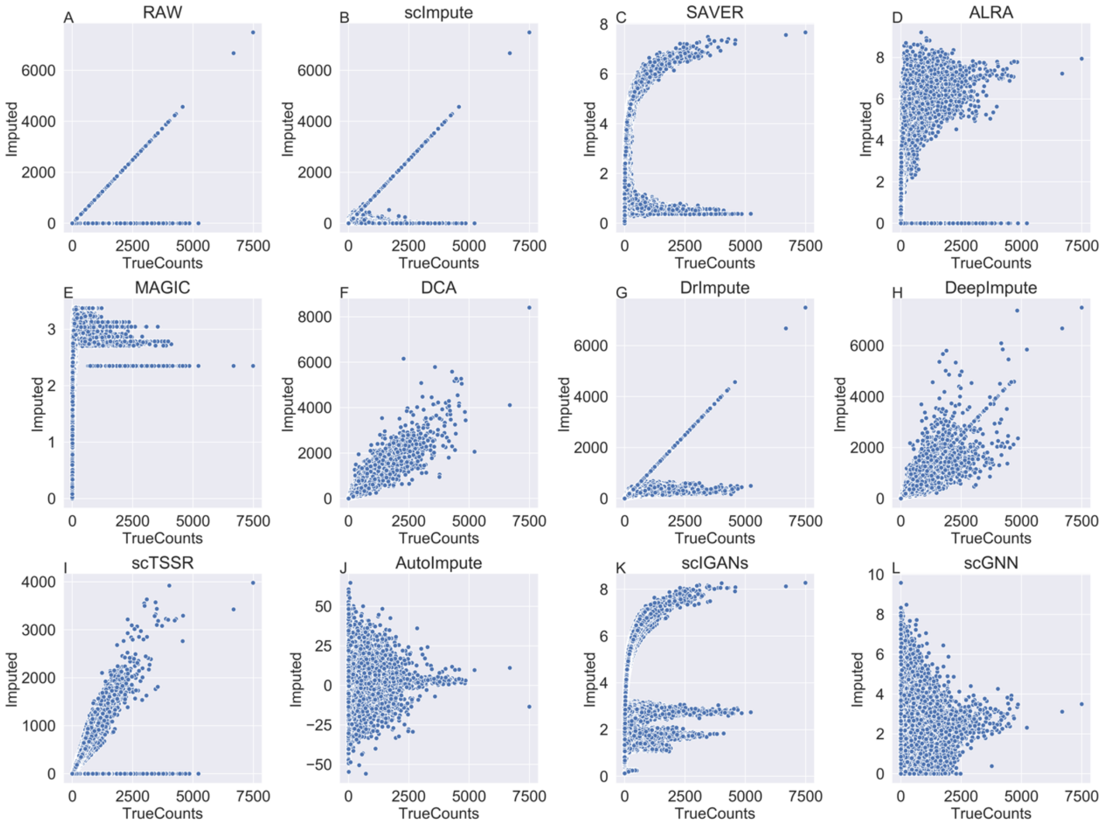

Figure S49. Scatter plots for the true gene expression values and imputed gene expression values for dataset with zero expression rate of 0.42. (A-L) Results from RAW data, and imputed data by scImpute, SAVER, ALRA, MAGIC, DCA, DrImpute, DeepImpute, scTSSR, AutoImpute, scIGANs, and scGNN. The *x-axis* represents true gene expression value, and the *y-axis* represents imputed gene expression value.

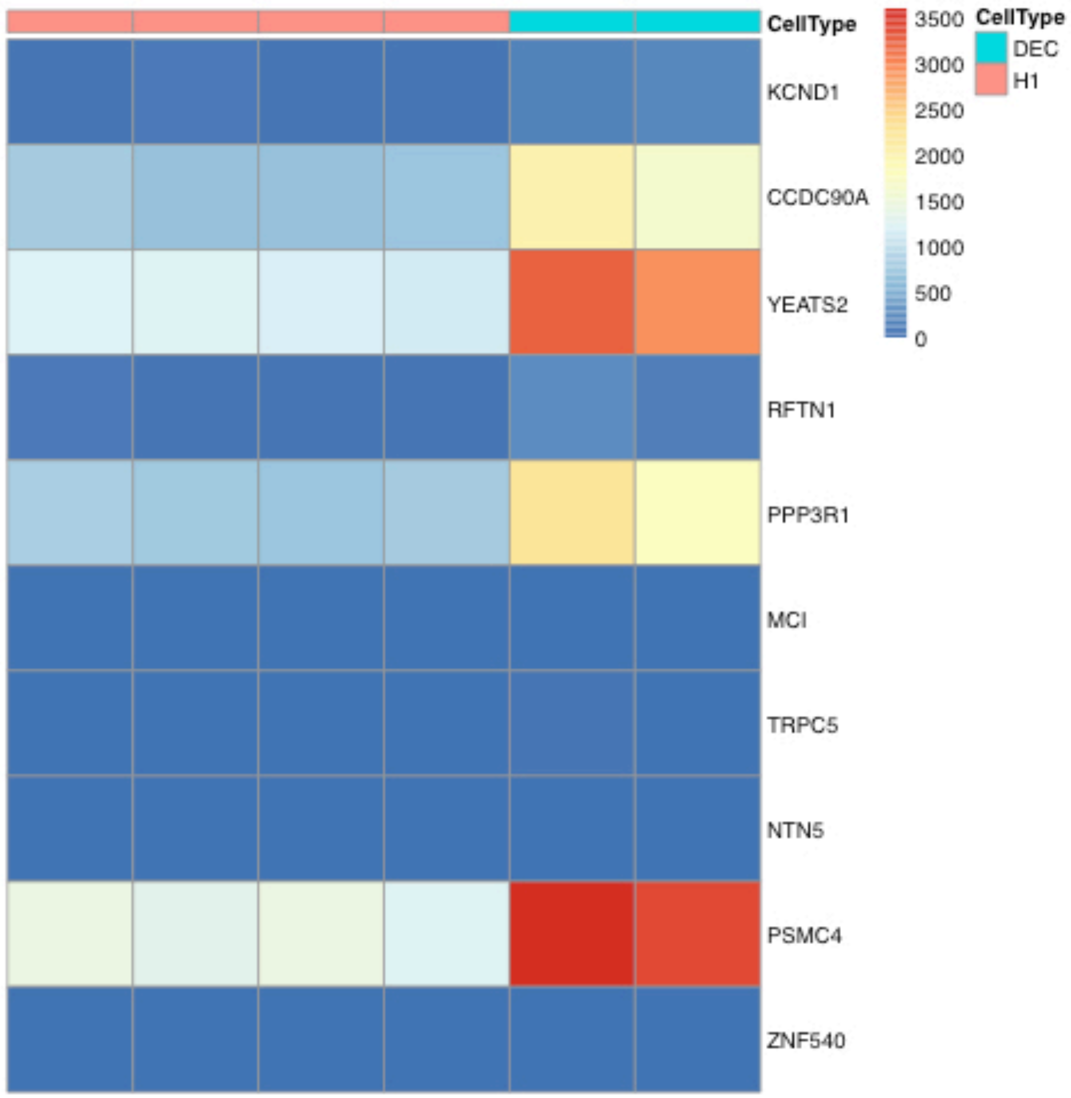

Figure S50. Heat map of top 10 significant genes in bulk RNA sequencing data. We extracted the top 10 genes with highest *P-Value* in bulk samples as reference. The cells are divided into two groups: H1 and DEC, which was shown in different colors (blue and pink).

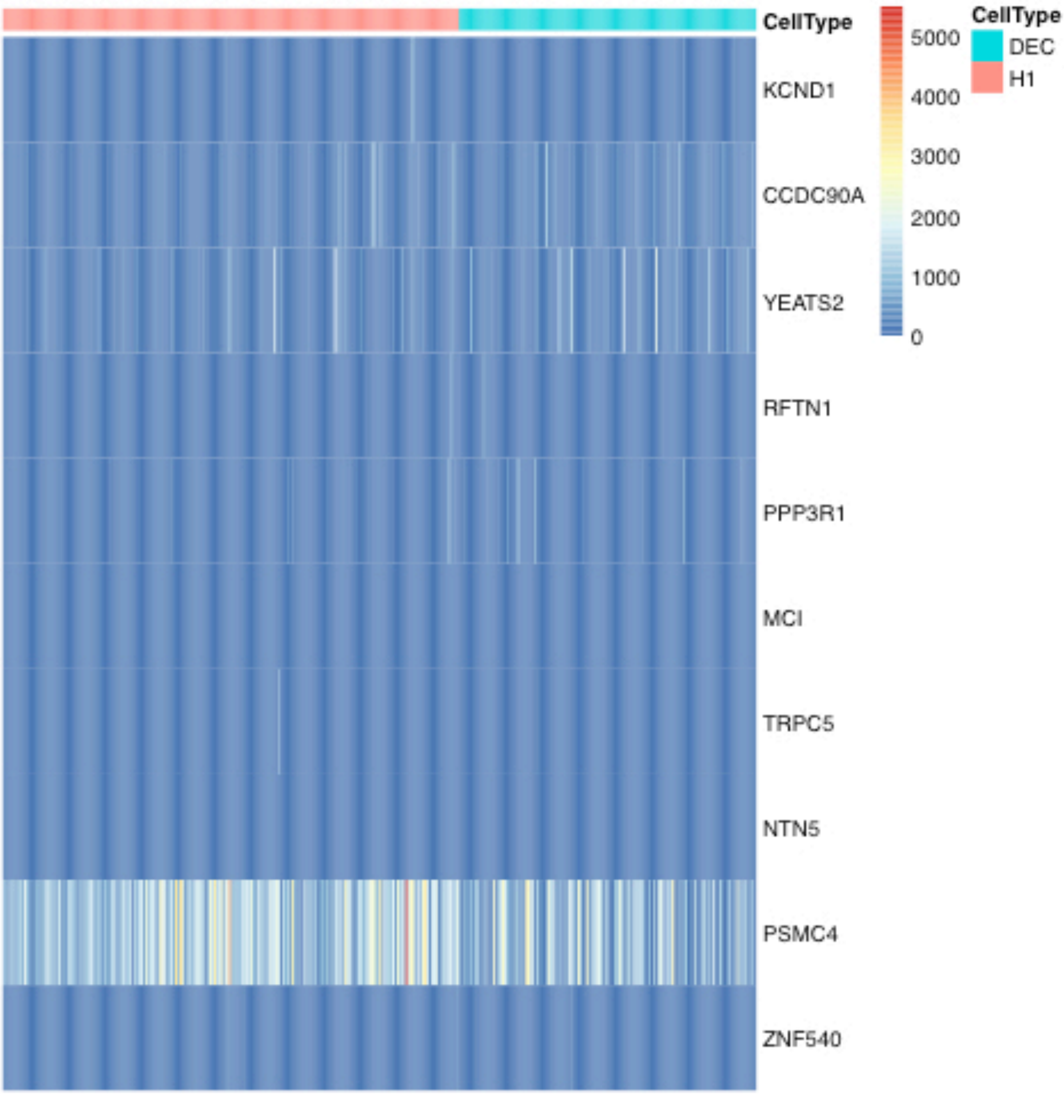

Figure S51. Heat map of top 10 significant genes in raw scRNA sequencing data. We extracted the top 10 genes with highest *P-Value* in bulk samples as reference. The cells are divided into two groups: H1 and DEC, which was shown in different colors (blue and pink).

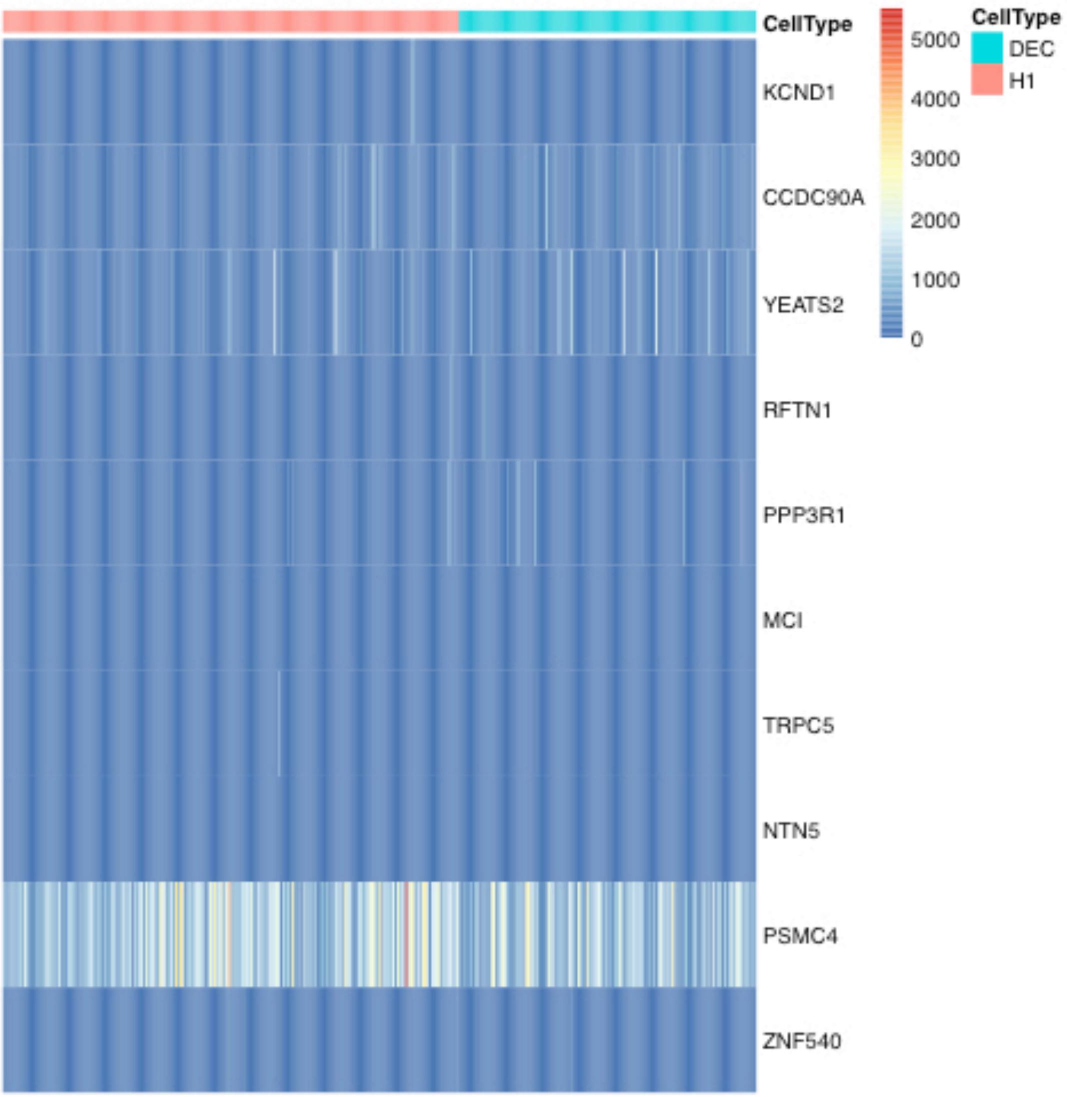

Figure S52. Heat map of top 10 significant genes data in imputed data from scImpute. We extracted the top 10 genes with highest *P-Value* in bulk samples as reference. The cells are divided into two groups: H1 and DEC, which was shown in different colors (blue and pink).

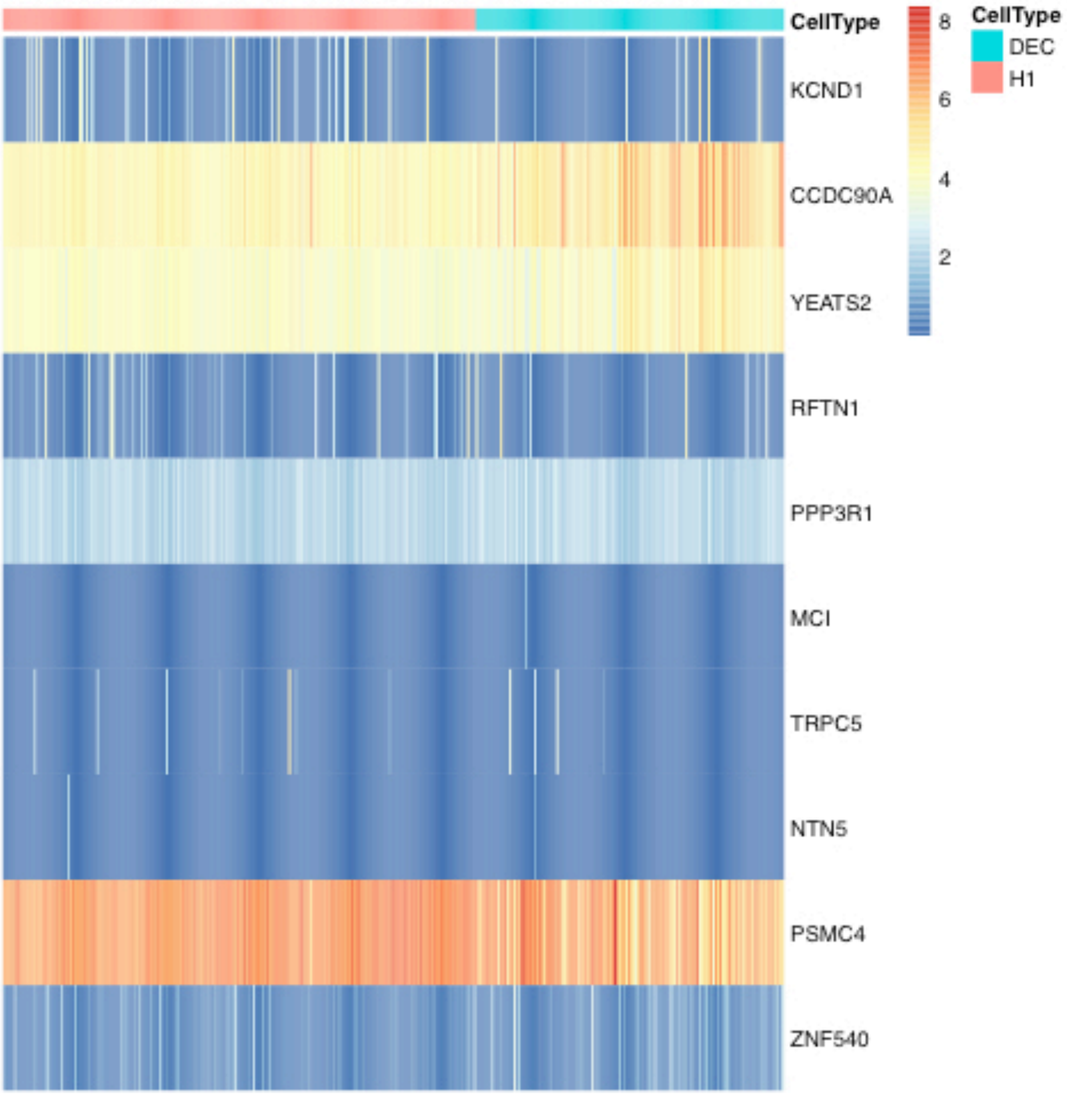

Figure S53. Heat map of top 10 significant genes data in imputed data from SAVER. We extracted the top 10 genes with highest *P-Value* in bulk samples as reference. The cells are divided into two groups: H1 and DEC, which was shown in different colors (blue and pink).

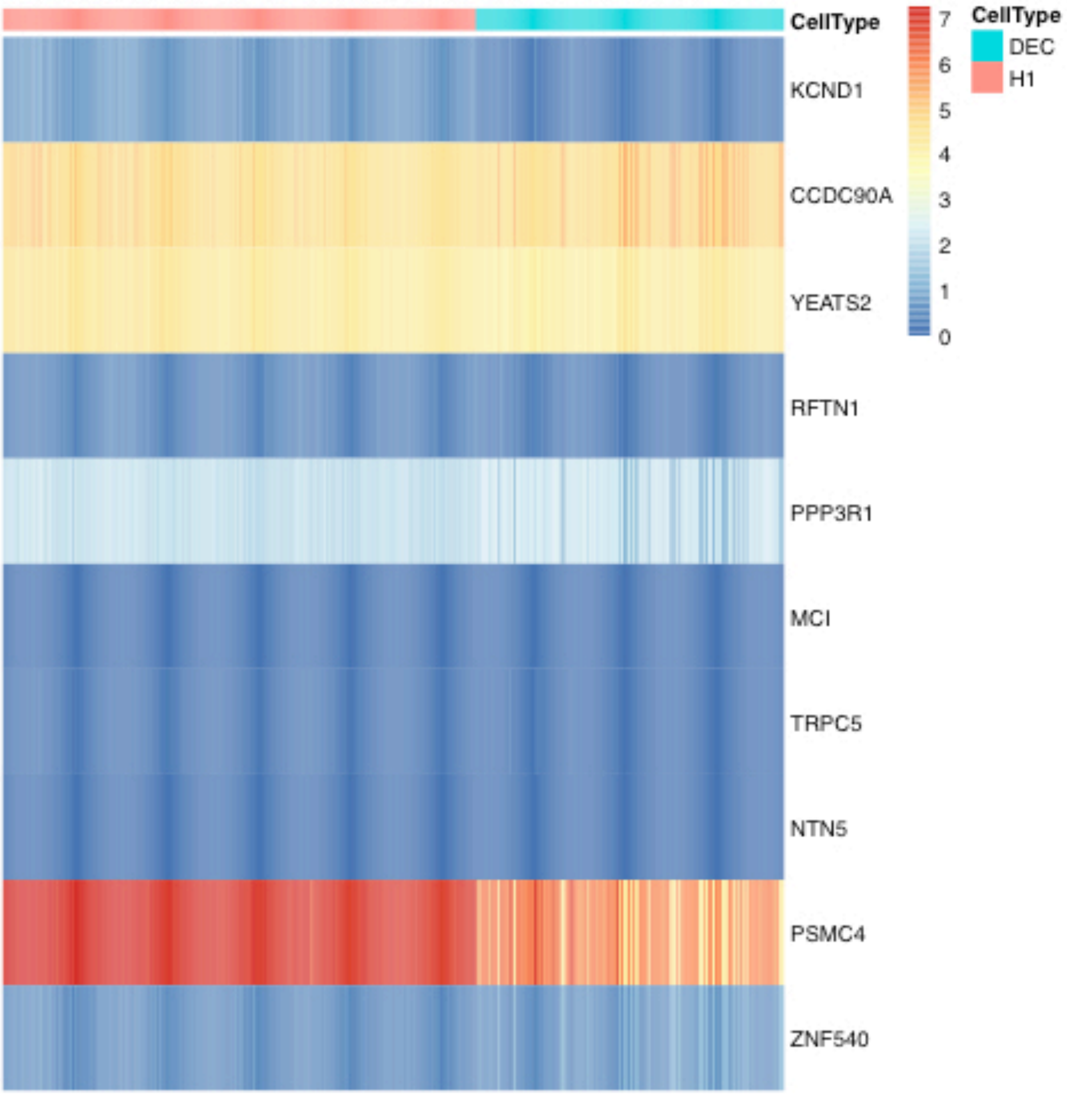

Figure S54. Heat map of top 10 significant genes data in imputed data from MAGIC. We extracted the top 10 genes with highest *P-Value* in bulk samples as reference. The cells are divided into two groups: H1 and DEC, which was shown in different colors (blue and pink).

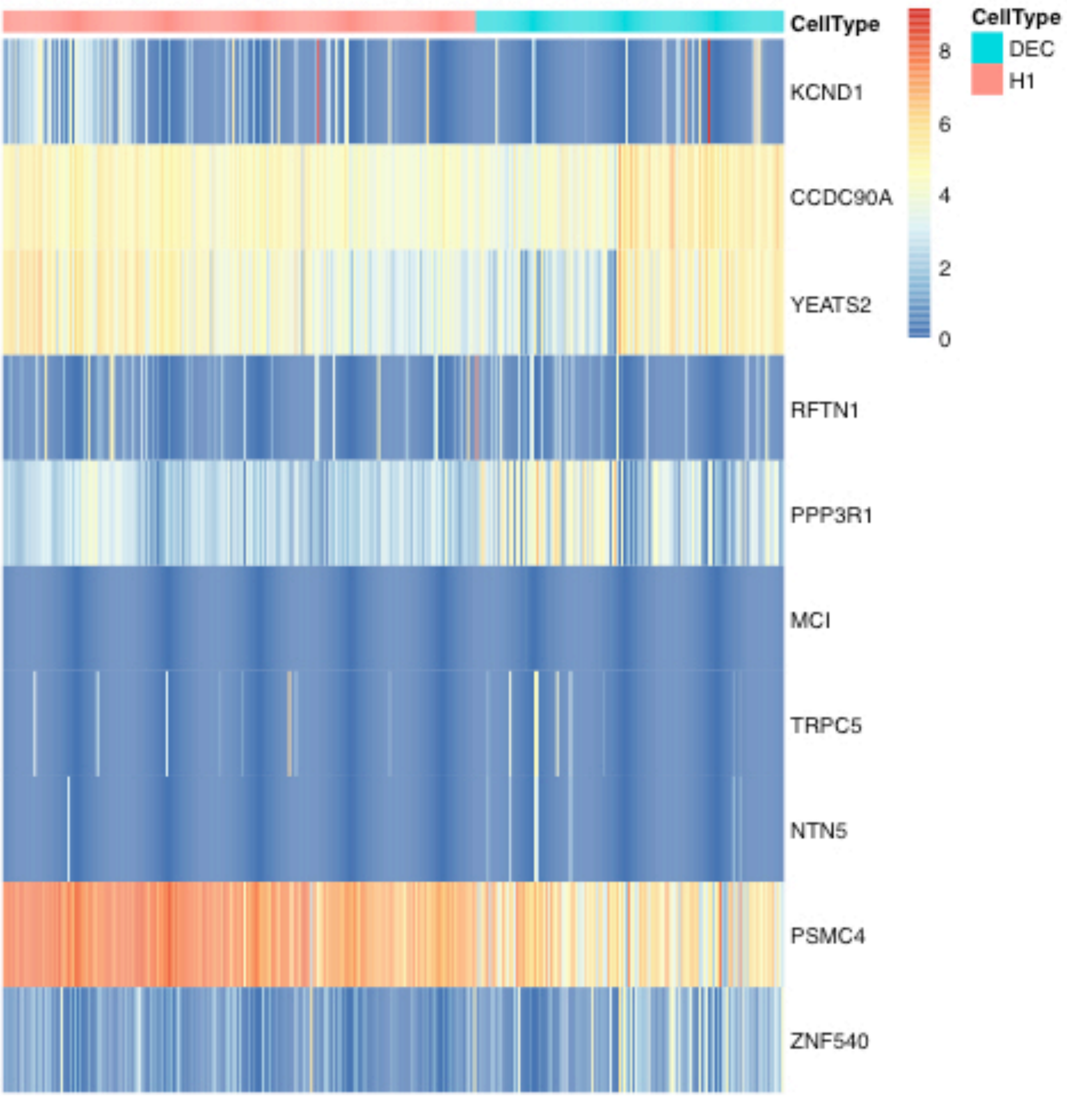

Figure S55. Heat map of top 10 significant genes data in imputed data from ALRA. We extracted the top 10 genes with highest *P-Value* in bulk samples as reference. The cells are divided into two groups: H1 and DEC, which was shown in different colors (blue and pink).

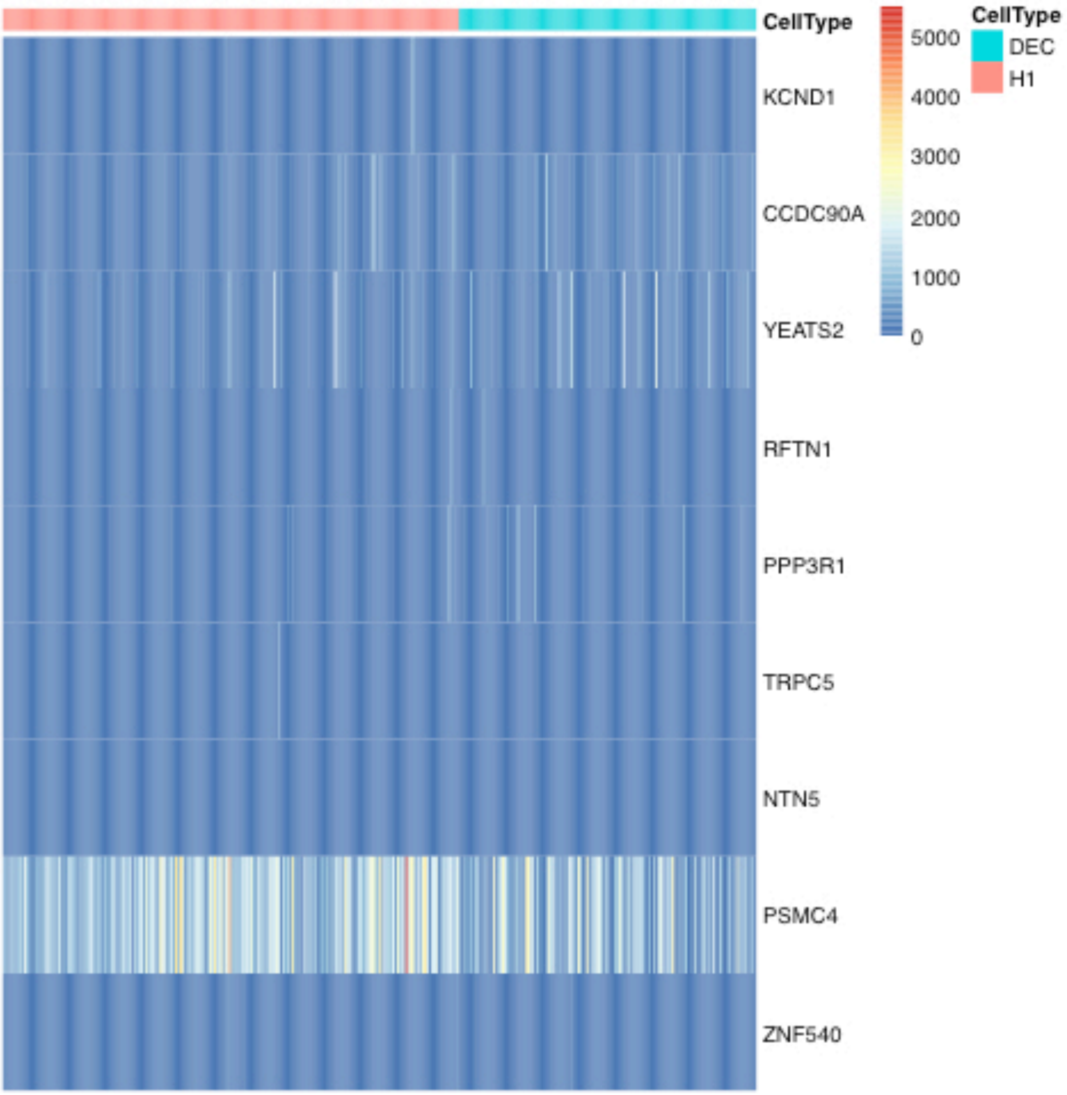

Figure S56. Heat map of top 10 significant genes data in imputed data from DrImpute. We extracted the top 10 genes with highest *P-Value* in bulk samples as reference. The cells are divided into two groups: H1 and DEC, which was shown in different colors (blue and pink).

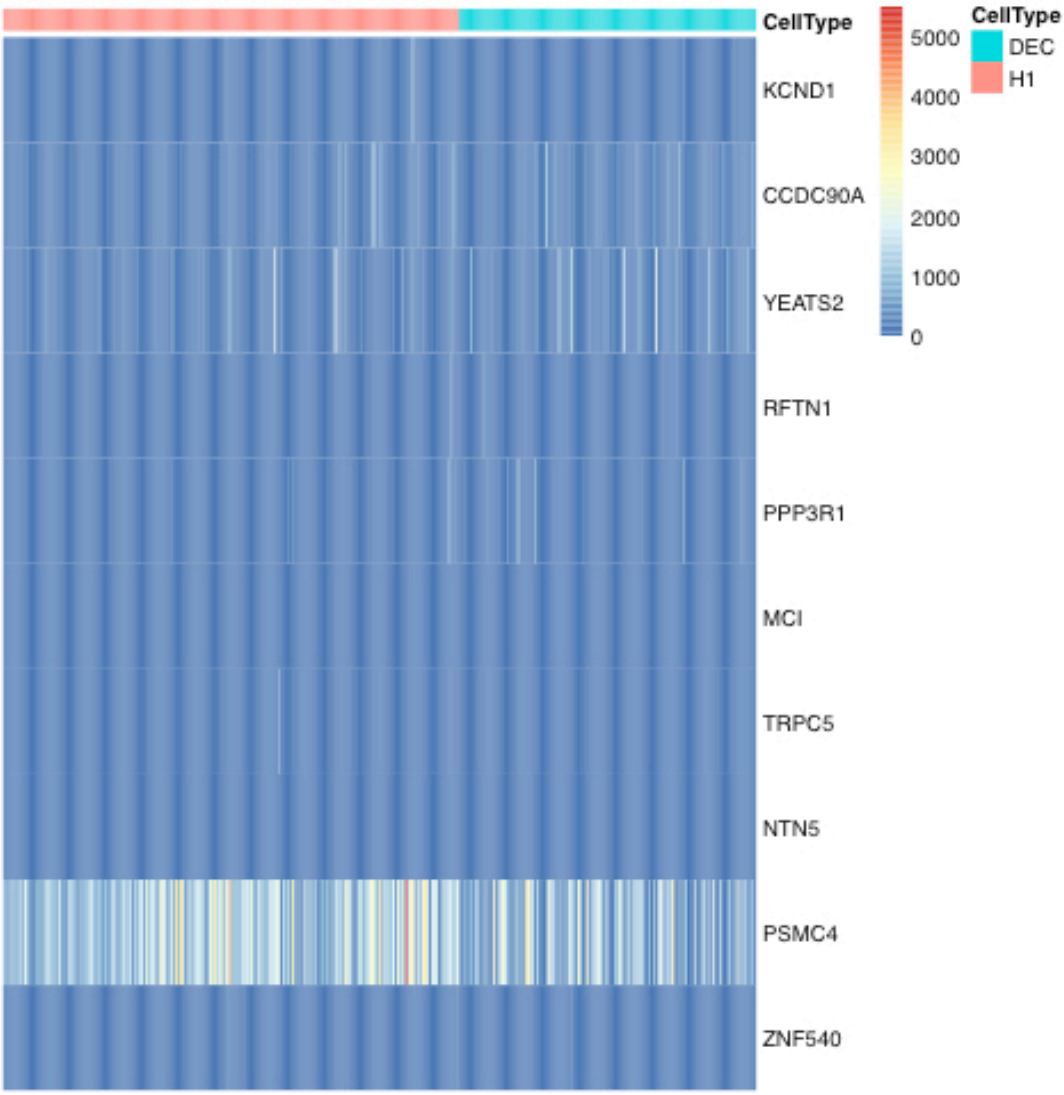

Figure S57. Heat map of top 10 significant genes data in imputed data from DeepImpute. We extracted the top 10 genes with highest *P-Value* in bulk samples as reference. The cells are divided into two groups: H1 and DEC, which was shown in different colors (blue and pink).

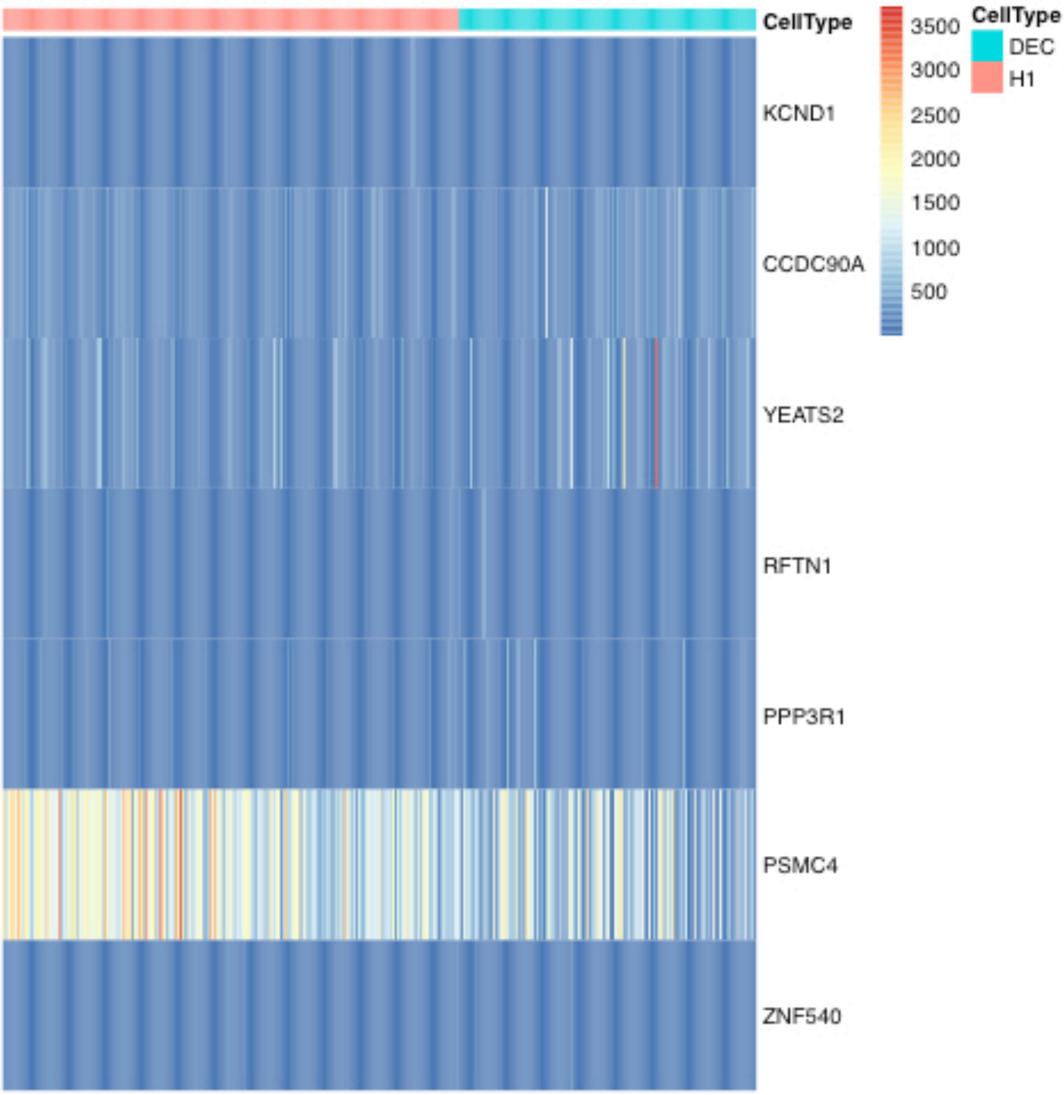

Figure S58. Heat map of top 10 significant genes data in imputed data from scTSSR. We extracted the top 10 genes with highest *P-Value* in bulk samples as reference. The cells are divided into two groups: H1 and DEC, which was shown in different colors (blue and pink).

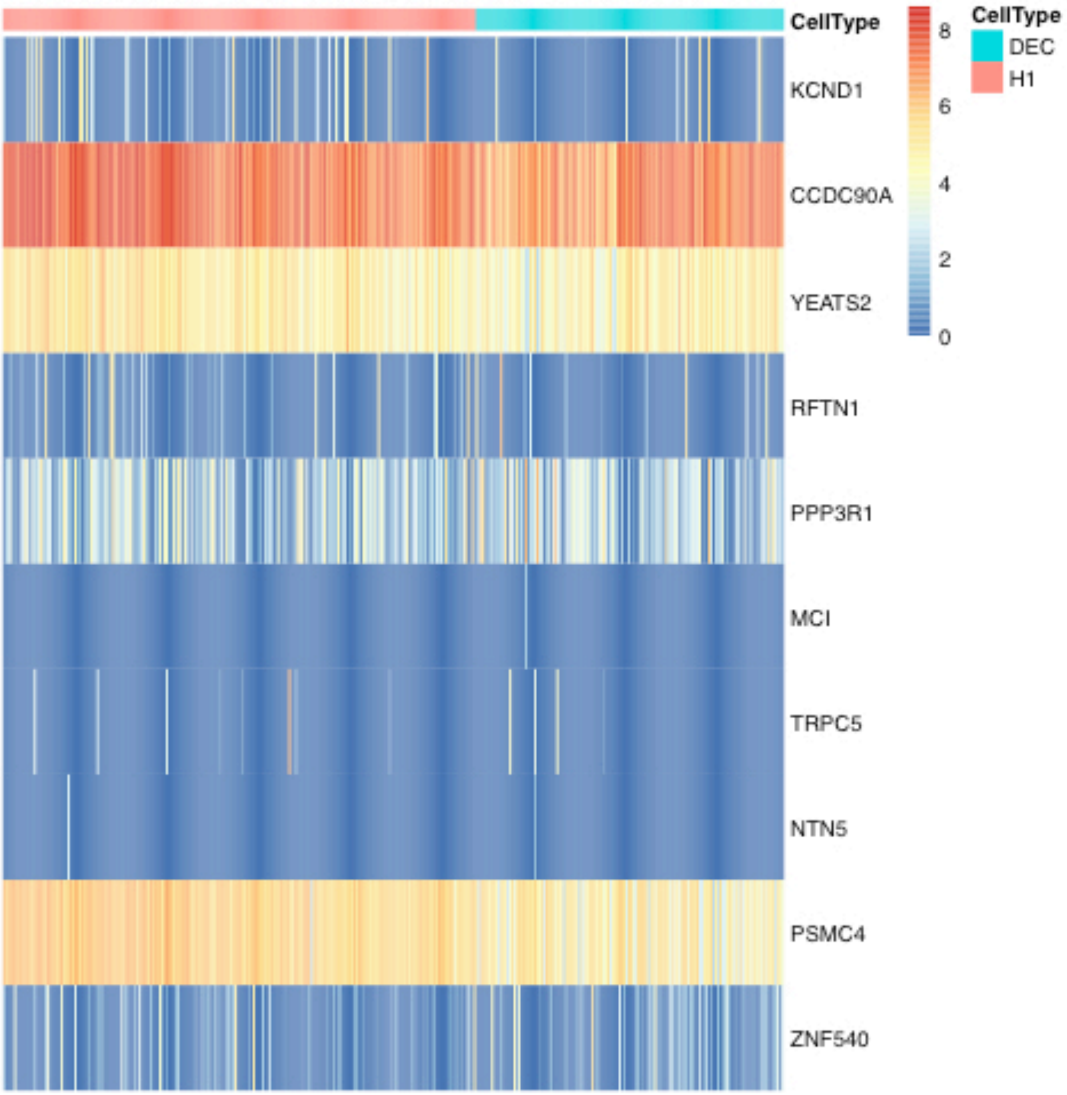

Figure S59. Heat map of top 10 significant genes data in imputed data from scNPF. We extracted the top 10 genes with highest *P-Value* in bulk samples as reference. The cells are divided into two groups: H1 and DEC, which was shown in different colors (blue and pink).

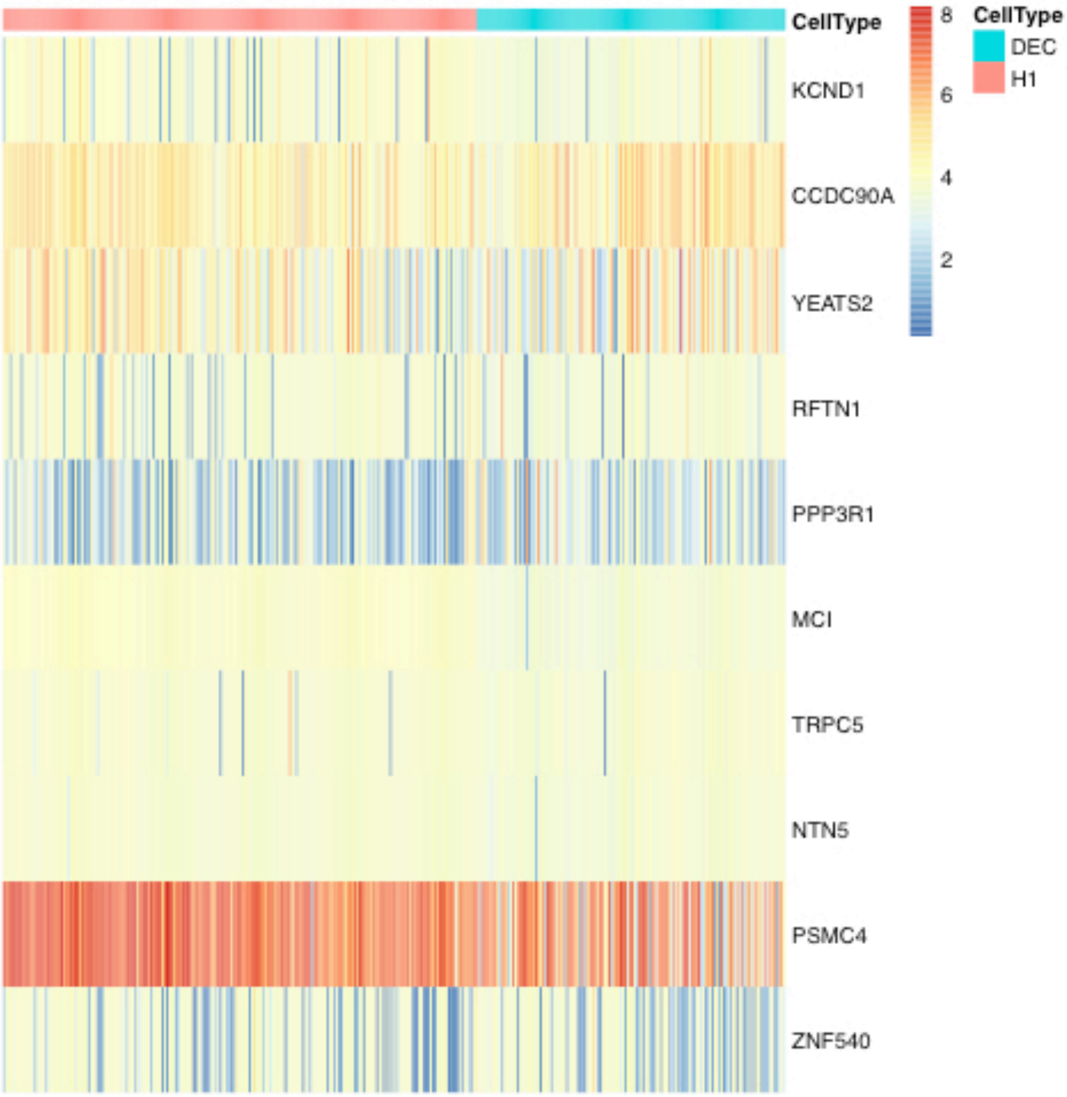

Figure S60. Heat map of top 10 significant genes data in imputed data from scIGANs. We extracted the top 10 genes with highest *P-Value* in bulk samples as reference. The cells are divided into two groups: H1 and DEC, which was shown in different colors (blue and pink).

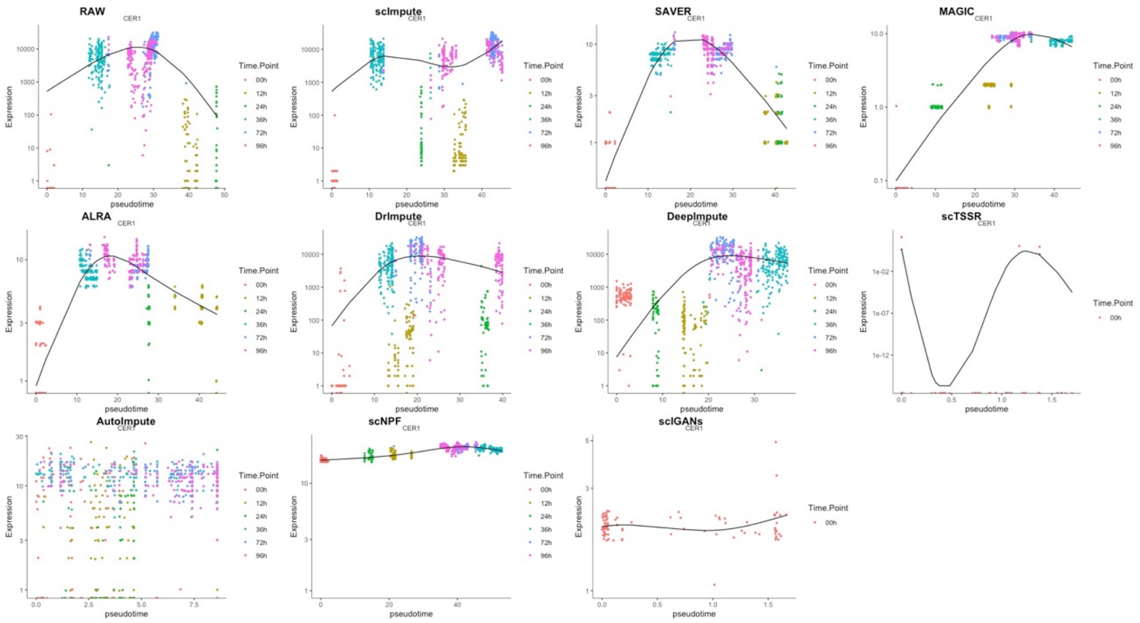

Figure S61. The cellular trajectories reconstructed by Monocle3 of DEC signature gene CER1.

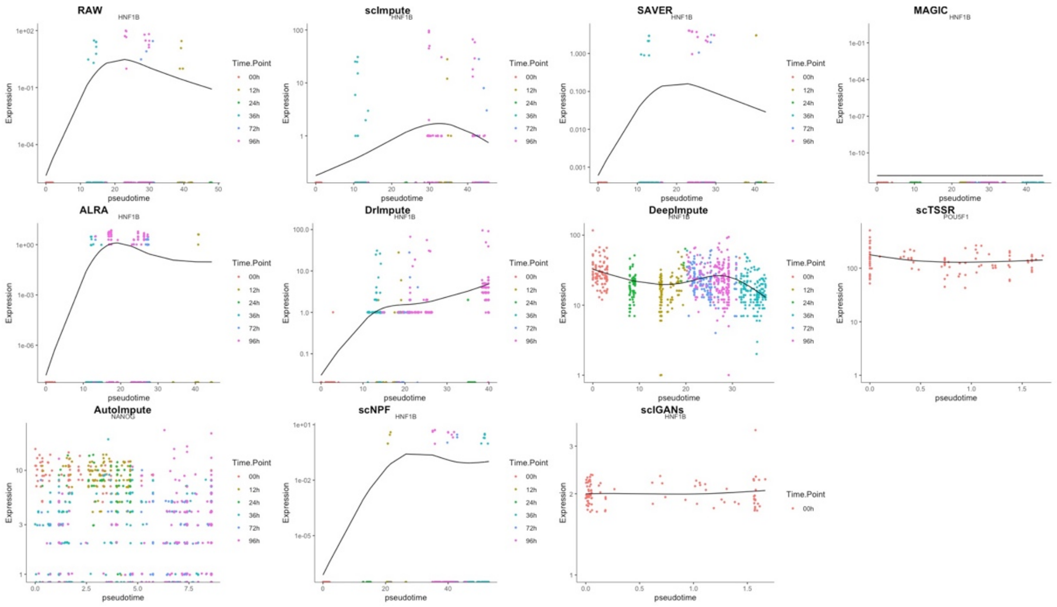

Figure S62. The cellular trajectories reconstructed by Monocle3 of DEC signature gene HNF1B.
